# Phylogeny and biogeography of Asthenopodinae with a revision of *Asthenopus*, reinstatement of *Asthenopodes*, and the description of the new genera *Hubbardipes* and *Priasthenopus* (Ephemeroptera, Polymitarcyidae)

**DOI:** 10.3897/zookeys.478.8057

**Published:** 2015-01-28

**Authors:** Carlos Molineri, Frederico F. Salles, Janice G. Peters

**Affiliations:** 1 Instituto de Biodiversidad Neotropical, CONICET (Argentine Council of Science & Technology), Universidad Nacional de Tucumán, M. Lillo 205, San Miguel de Tucumán, 4000, Tucumán, Argentina, UNASUR; 2 Laboratório de Sistemática e Ecologia de Insetos, Depto. de Ciências Agrárias e Biológicas, Universidade Federal do Espírito Santo, CEP 29.933-415, São Mateus, ES, Brazil; 3 Florida A&M University, Tallahassee, Florida, USA

**Keywords:** Ephemeroptera, Ephemeroidea, Fossoriae, vicariance, evolution, Neotropics, Campsurinae, *Campsurus*, *Tortopus*, *Tortopsis*, *Povilla*, *Languidipes*

## Abstract

The Neotropical species of Asthenopodinae are revised in a formal phylogenetic context. The five known species of *Asthenopus* Eaton, 1871, together with other five new species were included in a cladistic analysis using morphological characters (continuous and discretes). Representatives of the Afro-Oriental group of the subfamily (*Povilla* Navás, 1912 and *Languidipes* Hubbard, 1984) were also included to test the monophyletic hypothesis traditionally accepted for the group. Additional taxa representing the other subfamilies of Polymitarcyidae were incorparated: *Ephoron* Williamson, 1802 (Polymitarcyinae) and *Campsurus* Eaton, 1868, *Tortopus* Needham & Murphy, 1924 and *Tortopsis* Molineri, 2010 (Campsurinae). A matrix of 17 taxa and 72 characters was analyzed under parsimony resulting in a single tree supporting the monophyly of the subfamily Asthenopodinae. Other results include the monophyly of the Afro-Oriental taxa (*Povilla* and *Languidipes*), the paraphyletic nature of Neotropical Asthenopodinae, and the recognition of four South American genera: *Asthenopus* (including *Asthenopus curtus* (Hagen), 1861, *Asthenopus angelae* de Souza & Molineri, 2012, *Asthenopus magnus* **sp. n.**, *Asthenopus hubbardi* **sp. n.**, *Asthenopus guarani* **sp. n.**), *Asthenopodes* Ulmer, 1924, **stat. n.** (including *Asthenopus picteti* Hubbard, 1975, **stat. n.**, *Asthenopodes traverae* **sp. n.**, *Asthenopodes chumuco* **sp. n.**), *Priasthenopus* **gen. n.** (including *Priasthenopus gilliesi* (Domínguez), 1988, **comb. n.**), and *Hubbardipes* **gen. n.** (including *Hubbardipes crenulatus* (Molineri et al.), 2011, **comb. n.**). Descriptions, diagnoses, illustrations and keys are presented for all Neotropical taxa of Asthenopodinae (adults of both sexes, eggs and nymphs). Additionally a key to the subfamilies and genera of Polymitarcyidae is included. A quantitative biogeographic analysis of vicariance is presented and discussed through the study of the “taxon history” of the group.

## Abstract (es)

Se revisan las especies neotropicales de Asthenopodinae en un contexto filogenético. Las cinco especies de *Asthenopus* Eaton, 1871, junto con otras cinco nuevas especies, son analizadas cladísticamente a partir de caracteres morfológicos externos (continuos y discretos). También se incluyen representantes del grupo Afro-Oriental de la subfamilia (*Povilla* Navás, 1912 y *Languidipes* Hubbard, 1984) para comprobar la hipótesis de monofilia tradicionalmente aceptada para el grupo. Se incorporaron taxones adicionales representando las otras subfamilias de Polymitarcyidae: *Ephoron* Williamson, 1802 (Polymitarcyinae) y *Campsurus* Eaton, 1868, *Tortopus* Needham & Murphy, 1924 y *Tortopsis* Molineri, 2010 (Campsurinae). Se analizó bajo parsimonia una matriz de 17 taxa y 72 caracteres resultando un sólo árbol que apoya la monofilia de Asthenopodinae. Otros resultados incluyen la monofilia del grupo Afro-Oriental (*Povilla* y *Languidipes*), la naturaleza parafilética de los Asthenopodinae neotropicales, y el reconocimiento de cuatro géneros sudamericanos: *Asthenopus* (incluyendo a *Asthenopus curtus* (Hagen), 1861, *Asthenopus angelae* de Souza & Molineri, 2012, *Asthenopus magnus* **sp. n.**, *Asthenopus hubbardi* **sp. n.**, *Asthenopus guarani* **sp. n.**), *Asthenopodes* Ulmer, 1924, **stat. n.** (*Asthenopus picteti* Hubbard, 1975, **stat. n.**, *Asthenopodes traverae* **sp. n.**, *Asthenopodes chumuco* **sp. n.**), *Priasthenopus* **gen. n.** (*Priasthenopus gilliesi* (Domínguez), 1988, **comb. n.**), y *Hubbardipes* **gen. n.** (*Hubbardipes crenulatus* (Molineri et al.), 2011, **comb. n.**). Se presentan descripciones, diagnosis, ilustraciones y claves para todos los taxones neotropicales de Asthenopodinae (adultos de ambos sexos, huevos y ninfas). Adicionalmente se incluye una clave para subfamilias y géneros de Polymitarcyidae. Se presenta un análisis biogeográfico cuantitativo de vicarianza y se discute la historia particular del grupo.

## Introduction

Polymitarcyidae Banks (Ephemeroptera) have long attracted the attention of freshwater biologist because their nymphs burrow tunnels in submersed wood, live in aquatic plants and sponges, and some inorganic sediment as clay, mud or sand (Arndt 1938, Hartland-Rowe 1958, Sattler 1967). Vejhabongse (1937) reported the damage caused by larvae of *Povilla* Navás, 1912 (Asthenopodinae) in woody structures. Leal et al. (2003) studied the capacity of *Campsurus* Eaton, 1868, larvae as bioturbators in soft mud bottom of Amazonian lakes; and *Tortopsis* Molineri, 2010, nymphs play an important role in the erosion of river clay banks (Molineri, unpubl.).

The family Polymitarcyidae is composed of three subfamilies (Polymitarcyinae, Asthenopodinae, and Campsurinae, Edmunds and Traver 1954, Bae and McCafferty 1995), and shows a broad distribution in the Holartic, Paleartic, Oriental and Neotropical regions (McCafferty 2004). Ogden et al. (2009) included one representative of each of these subfamilies in a combined molecular and morphological study of the entire order Ephemeroptera – in their work the traditionally known relationships were recovered with Polymitarcyinae spliting first and then Campsurinae as sister to Asthenopodinae. The monophyly of the submafilies and the relationships inside Asthenopodinae were not formally tested yet, except for some groups in Campsurinae (*Tortopus* and *Tortopsis*, Molineri 2010; *Campsurus*, Molineri and Salles 2013). The Neotropical genera of the family include *Asthenopus* Eaton, 1871 (Asthenopodinae), *Campsurus*, *Tortopus* Needham & Murphy, 1924, and *Tortopsis* (Campsurinae). All Campsurinae genera are represented in the Nearctic by at least one species, but the majority of them are known from tropical and subtropical South America, as is the case with all the species of *Asthenopus* (Domínguez et al. 2006).

*Asthenopus* is presently classified in Asthenopodinae, together with the Afro-Oriental *Povilla* Navás and *Languidipes* Hubbard, 1984 (Edmunds and Traver 1954, Baumgardner et al. 2012) but a formal phylogenetic analysis supporting this subfamily as monophyletic is wanting. Some of their shared features (e.g., ring-like prothorax, parallel ICu veins in forewings) may prove to be plesiomorphies, others (e.g., straight CuA, median remnant of styliger plate) are absent in *Languidipes* (Baumgardner et al. 2012), and at least one putative synapomorphic state, the loss of basal segment of the forceps in *Asthenopus* and Campsurinae indicate a probable sister relation among all the South American genera (Kluge 2004). Additionally, Kluge (2004) treated *Povilla* and *Asthenopus* as synonyms.

*Asthenopus* is presently known from five South American species: *Asthenopus curtus* (Hagen), 1861, *Asthenopus crenulatus* Molineri, Cruz & Emmerich, 2011, *Asthenopus gilliesi* Domínguez, 1988, *Asthenopus picteti* (Hubbard), 1975, and *Asthenopus angelae* de Souza & Molineri, 2012. All of them are known at least from male adults, but only two (*Asthenopus curtus* and *Asthenopus angelae*) are known also from the nymphs. Nevertheless, the nymphal stage of *Asthenopus curtus* is redescribed here because it has been described from missidentified specimens (Sattler 1967, Berner 1978, Domínguez 1989). *Asthenopodes* Ulmer, was described from males of a single species (Ulmer 1924), presently *Asthenopus picteti* (Hubbard). *Asthenopodes* was proposed as a junior subjective synonym of *Asthenopus* by Hubbard and Domínguez (1988) based on the discovery of *Asthenopus gilliesi* Domínguez (1988) that showed intermediate characters between both genera.

The main objective of the present paper is to evaluate in a phylogenetic context the validity of the generic groups of Asthenopodinae (e.g., *Asthenopus*, *Asthenopodes*, *Povilla* and *Languidipes*). As a result we propose two new genera, revalidate *Asthenopodes* at the generic rank, and describe five new Neotropical species.

Additionally we describe and illustrate some unknown stages of previously known species, propose a key to the subfamilies and genera of Polymitarcyidae, and to the species of Neotropical Asthenopodinae. New country records are given and biogeographical aspects of the group are discussed.

## Material and methods

### Material deposition

Material is deposited in the following Institutions: CUIC (Cornell University Insect Collection, Ithaca, NY), FAMU (Florida A&M University, Tallahassee, FL), IFML
(Instituto-Fundación Miguel Lillo, Tucumán), IBN (Instituto de Biodiversidad Neotropical, Tucumán), MACN (Museo Argentino de Ciencias Naturales, Buenos Aires), MECN (Museo Ecuatoriano de Ciencias Naturales, Quito), MUSENUV (Museo de la Universidad del Valle, Cali), RBINS (Royal Belgian Institute of Natural Sciences, Brussels), FCE-Ep (Facultad de Ciencias, Entomología, Montevideo, Uruguay), CZNC (Coleçao Zoológica Norte Capixaba, São Mateus, Espírito Santo), INPA (Instituto Nacional de Pesquisas da Amazônia, Manaus), and ZMH (Zoologisches Museum Hamburg).

### Morphological characters

Characters are scored from external morphological features of adults (male imago unless otherwise indicated), nymphs and eggs. Dissected parts of the nymphs and adults were mounted on microscope slides using Canada Balsam, except wings that were mounted dried. All the material is preserved in ethyl alcohol 96%. Photographs were taken using a NIKON SMZ-10 stereomicroscope or a microscope, with a Nikon D5000 digital camera; some pictures were modified with Combine ZP (Hadley 2010). Line drawings were done using a camera lucida attached to a microscope. Structures used for SEM study were dehydrated in a graded ethanol series, dried by the critical point method, sputter coated with gold and observed with a JEOL 35 CF scanning electron microscope.

The measures and ratios used in some characters are explained and illustrated to permit repeatability. To score variation in shape of genitalic structures (chars. 12–17) some measures were defined (see Appendix 1). Ratios between some of them were used instead of the original data to avoid the spurious differences occasioned by the great variation in size common in the group. Characters 0 to 11 also are ratios or counts to represent characteristics commonly used in the taxonomy of the group, for example the length of forelegs of male and its relation to other structures. Fore and hind wings are abbreviated FW and HW respectively throughout the text. A complete list of the characters, their definitions and character states is given in Appendix 1.

### Taxa

A matrix of 17 taxa and 72 characters was constructed (Appendix 3). Trees were rooted in *Ephoron* Williamson, 1802 (Polymitarcyinae). Four representatives of Campsurinae were also included as additional outgroups (*Campsurus violaceus* Needham & Murphy, 1924, *Campsurus vulturorum* Emmerich & Molineri, 2011, *Tortopus harrisi* Traver, 1950, and *Tortopsis sarae* Domínguez, 1985). The African and Oriental Asthenopodinae were represented in the analysis by *Povilla adusta* Navás, 1912 and *Languidipes corporaali* (Lestage), 1922. All species of Neotropical Asthenopodinae were scored, including the five species of *Asthenopus* and five new species described here. The formerly described species include: *Asthenopus curtus* (Hagen), *Asthenopus picteti* (Hubbard), *Asthenopus gilliesi* Domínguez, *Asthenopus crenulatus* Molineri et al., and *Asthenopus angelae* de Souza & Molineri. The material studied includes type and fresh material of all species, detailed under each specific section.

Outgroups were scored either from fresh material (*Ephoron album* Say, 1823: 2 nymphs and 1 male imago from USA, Alabama, Perry County, Cahaba River, 27-vi-1968, Peters et al. cols.; *Povilla adusta*: 1 nymph from Afrique, Mali, Bas. Sénégal, Riv. Falémé, 13-xi-1984, ORSTOM col., 16 male imagos from Afrique, Mali, Bas. Niger, Riv. Niger, Gao, 7-ix-1987, ORSTOM col.; 3 male and 2 female imagos from Afrique, Guinée, Bas Senegal, Riv. Bafing Loc., Timbo-Dabola (route), 31.01.1987, ORSTOM col.) or from figures and descriptions (*Ephoron* spp from Ishiwata 1996; *Languidipes corporaali*, Hubbard 1984, Baumgardner et al. 2012). *Campsurus paraquarius* Navás (1920), treated here as nomen nudum in Asthenopodinae, was not included in the matrix because of lack of data (only a figure of the Cu field of male is known, figure 1 in Navás 1920).

### Cladistic analysis

Searches were conducted in TNT (Goloboff et al. 2008) under implied weights (k = 3), and using the “implicit enumeration” command (under “Analyse”). Implied weighting was suggested to ameliorate the problems of scaling in continuous characters (Goloboff et al. 2006). By the implicit enumeration, the complete universe of possible trees for the matrix is calculated, then picking the shortest ones. All characters were treated as non-additive except for continuous characters (chars. 0 to 26). Absolute and relative Bremer supports were calculated with 2000 suboptimal trees (up to 8 steps longer than shortest tree), with the commands “suboptimal” (under “Analyse”) and “Bremer Supports” (under “Trees”). Frequency difference (GC, Goloboff et al. 2003), using 400 replications of symmetric jacknifing, was also calculated as a measure of group support. The optimizations of individual continuous characters given in Figs 21 and 22 were obtained in TNT using the commands “Optimize: Characters: Character Mapping”.

### Biogeographical methods

All the available geographic records (448 records for 17 taxa) of the species or genera included in the phylogeny were compiled. Concerning *Asthenopus* species, only records from specimens (or photographs) revised by us were included, since much confusion exists in the literature in relation to *Asthenopus curtus* and similar species. All records are exact points of occurrence except some for *Ephoron* species from North America and Europe, which were only roughly approximated from the maps in McCafferty (1975) and de Jong (2012), respectively. Some of the records (ca. 100) for *Tortopsis*, *Ephoron* and *Povilla* were downloaded from Global Biodiversity Information Facilities (http://www.gbif.org/, last accesed February 10th 2014).

The biogeographical analysis was performed through spatial analysis of vicariance (Arias et al. 2011), a method that reconstructs taxon biogeographic history by looking at disjoint sister pairs in a given phylogeny. Besides a cladogram, it uses as input the distributional records of the terminals; and is implemented in the software VIP (Vicariance Inference Program) available at http://www.zmuc.dk/public/phylogeny/vip (Arias 2010). A grid of 1° × 1° was used (maximum fill = 0) in a world vegetation map (obtained in http://neo.sci.gsfc.nasa.gov) to represent distributions as absence/presence data in each cell. The “OR reconstruction” was used under the default settings except that cost of distribution removal was set to 1.5, and cost of partial removal to 0.75. Reconstructions using different grid size (5° × 5°) and costs were also conducted for comparative purposes, but results were similar.

## Results

### Phylogeny and taxonomic status of genera and species

**Figure 1.**
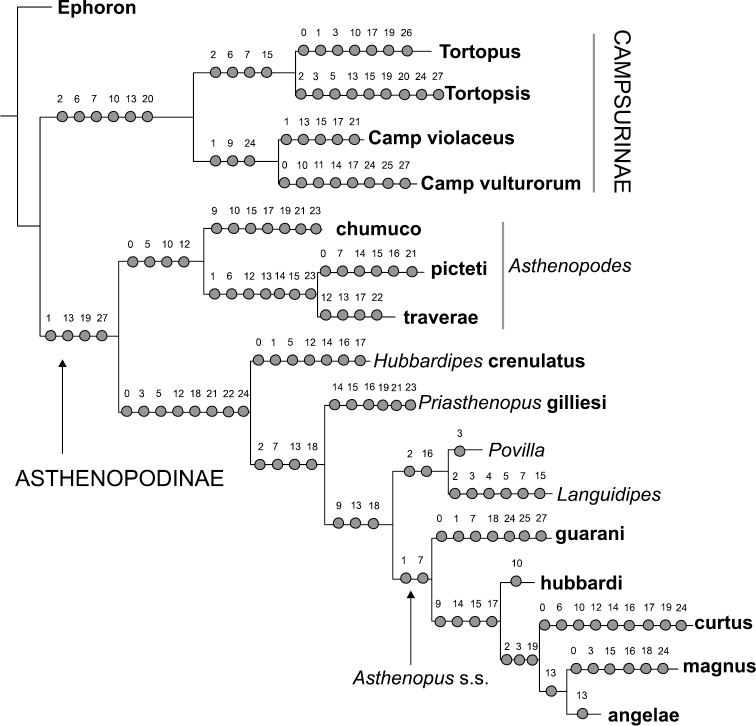
Most parsimonious tree with changes on continuous characters (number above circles are character numbers, see Appendix 1).

**Figure 2.**
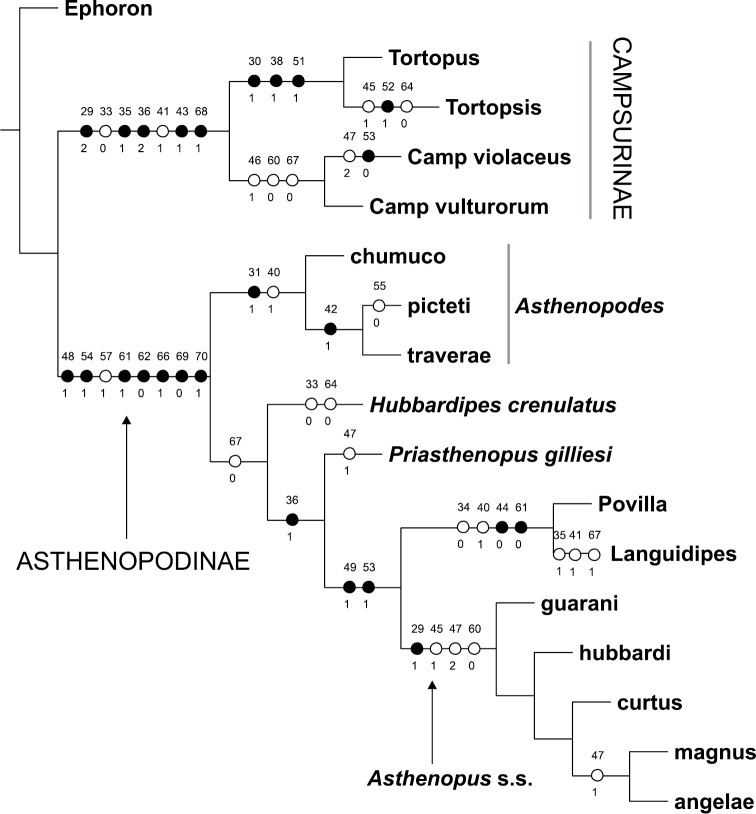
Most parsimonious tree (same for Fig. 1) showing changes on discrete characters (number above and below circles are character and state number, respectively, see Appendix 1). Black circles indicate unique apomorphies.

Parsimony under implied weights (IW) resulted in a single tree (Figs 1–2), with an adjusted homoplasy of 14.64 and a total fit of 54.36. Neotropical Asthenopodinae was not recovered as a monophyletic group: *Asthenopus* s.s. is sister to the Afro-Oriental clade (*Povilla*-*Languidipes*). *Asthenopodes* is revalidated as a distinct genus since its type species (*Asthenopus picteti* Hubbard) was recovered in a group with other two new species. *Asthenopodes* stat. n. presents defining characters in all stages (see below). The species described from adults by Molineri et al. (2011) as *Asthenopus crenulatus* is now known in all the stages (see below) and a new genus *Hubbardipes* is proposed for it. *Hubbardipes* gen. n. is supported as sister to the remaining Asthenopodinae (i.e., excluding *Asthenopodes*). Another new monotypic genus, *Priasthenopus* gen. n. is proposed for *Asthenopus gilliesi*, only known from adults and eggs. *Priasthenopus* is sister to the clade *Povilla* + *Asthenopus*.

**Figure 3.**
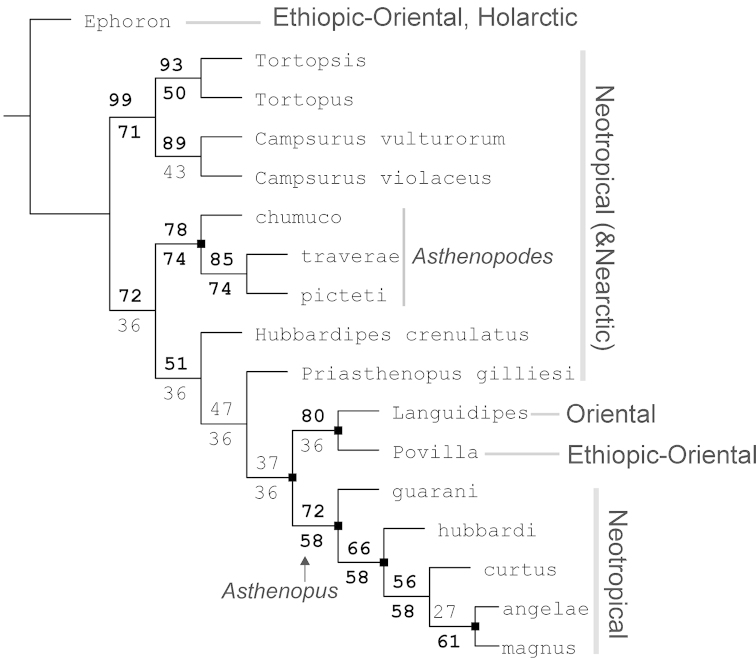
Group support and vicariant nodes. Numbers above branches indicate GC values (400 replicates, Symmetric Resampling), below branches Relative Bremer supports (from 2000 trees, up to 8 steps longer) are indicated. Support values higher than 50 are bolded. Black squares indicate nodes with allopatric descendants (see text for explanation).

The type species of *Asthenopus* (*Asthenopus curtus*) forms a clade with *Asthenopus angelae* and three new species. Synapomorphies of each clade are numerous; changes on continuous and discrete characters are presented separately in Figs 1 and 2, respectively. For details on changes see generic diagnosis and Appendix 2. Group support is strong for most clades (Fig. 3).

Summarizing, the taxonomic changes needed to adjust formal classification with the phylogeny include: 1) revalidation of *Asthenopodes* as a distinct genus and the corresponding new combination for its type species (*Asthenopodes picteti*), 2) the erection of *Hubbardipes* gen. n. and the new combination *Hubbardipes crenulatus* (Molineri et al.) for its single species, and 3) the erection of *Priasthenopus* gen. n. and the new combination *Priasthenopus gilliesi* (Domínguez). This phylogeny also was the framework used to describe the five new species in the corresponding genus (see “Descriptions”): *Asthenopodes traverae*, *Asthenopodes chumuco*, *Asthenopus hubbardi*, *Asthenopus magnus*, and *Asthenopus guarani*.

An additional taxonomic change is proposed for *Campsurus paraquarius* Navás (1920) that undoubtedly pertains to Asthenopodinae as recognized by Lestage (1923) and Kluge (2004), most likely it is related with *Priasthenopus gilliesi* (Domínguez). *Campsurus paraquarius* is here considered a nomen nudum (see discussion under *Priasthenopus* below).

### Asthenopodinae

The subfamily Asthenopodinae is composed in our analysis by four main groups, three Neotropical (*Asthenopodes*, *Hubbardipes*, and *Priasthenopus*) and one vicariant (Neotropical *Asthenopus* vs Ethiopic-Oriental *Povilla* + *Lanquidipes*) (Fig. 3). *Asthenopodes* is sister to the remaining groups, then *Hubbardipes* and *Priasthenopus* splits consecutively and finally *Asthenopus* is sister to *Povilla* + *Languidipes*.

Asthenopodinae is defined by numerous derived character states (see Appendix 2 for details) including: shorter male foretibia, wider forceps, shorter female cerci, female sternum VIII with anteromedian keel; eggs with large and small disk-like chorionic structures; nymphs with wider mandibular tusks, tusk with two or three apical denticles (right and left tusks respectively) and with a small basal tubercle on outer dorsal surface, occipital region strongly expanded and convex, and foretarsal claws with marginal row of denticles.

It is interesting to note some characters that simultaneously change in opposite “directions”, showing a certain tendency in *Asthenopodes* but the contrary in it sister group (remaining Asthenopodinae). For example the following features define *Asthenopodes* and its sister group: the male foretibia is thinner distally (vs wider in the remaining Asthenopodinae, Figs 20, 22A), median remnant of styliger plate shorter (vs longer), larger HW (vs smaller, Fig. 22B). Other synapomorphic changes associated to Asthenopodinae excluding *Asthenopodes* are given in Appendix 2.

The sister relation between *Priasthenopus* and the clade *Povilla* + *Languidipes* + *Asthenopus* is supported by (see Appendix 2, node 23): additional shortening of foreleg, shorter and less anastomosed cross veins in the margin of FW, additional increase in forceps width, even wider and shorter female prothorax.

The clade formed by *Asthenopus* together with the Afro-Oriental genera share (Appendix 2): even wider forceps, shorter and less anastomosed intercalary veins in both wings, slightly shorter female pronotum, female sternum VIII with long and slender anteromedian keel, sockets small, and nymphal mandibular tusks with large basal tubercle in inner margin (this last state may be present also in *Priasthenopus gilliesi*, but nymphs remain unknown).

*Povilla* and *Languidipes* form a monophyletic group because both show shorter forelegs in male, blade-like penes relatively straight not curved inward, MP_2_ shorter than IMP in FW (non-unique, also in Campsurinae), IMP connected to MP_1_, forceps two-segmented, pedestals much narrower basally, and nymphal left mandibular tusk with an additional apical denticle.

### Campsurinae

In spite of Campsurinae being only marginally represented in the matrix, we consider useful to note the derived character states defining the subfamily and genera (see Appendix 2 for details): much shorter forelegs in male, slender forceps, ICu_1_ joins hind margin of FW close to tornus while ICu_2_ joins it on basitornal margin, intercalary marginal veins absent or if present not anastomosed, median remnant of styliger plate absent, double penial arm articulation (sternum IX and base of pedestal), nymph with dorsum of head with dense patches of short setae on frons. *Campsurus* is very sparsely represented in the matrix (only 2 spp from more than 40 known) so only the character states defining *Tortopus* + *Tortopsis* may have some interest: slender penes, male foreleg slightly shorter, foretarsal segment 5 apically trilobed, intercalary marginal veins rarely present in male FW, HW anal area with many crossveins forming a network, forceps two-segmented.

### Evolution of selected features

Variation in the relative length of male foreleg segments has been repeatedly used by different authors in the taxonomy of the family. One of our characters concerning this variation (char. 1, ratio length tarsal segment 2/tibia) presented many changes: most important are those defining the genus *Asthenopus* and the pair *Asthenopodes traverae* – *Asthenopus picteti*, indicating opposite changes in these nodes (encircled arrows in Fig. 21A).

In the nymphs, the robustness of the mandibular tusks is an important feature distinguishing different subfamilies. The optimization of character 27 (ratio length/width of tusk, Fig. 21B) shows an apomorphic change in the base of Asthenopodinae, indicating that the slender tusks of Campsurinae are plesiomorphic.

Trends in some charecteristics are markedly manifested, for example the width of male foretibia (char. 0 ratio width tibia/tarsal segment 2, Fig. 22A) decreases at the base of *Asthenopodes* and become thinner again later in *Asthenopodes picteti*. On the contrary, it becomes more robust in the base of the sister clade to *Asthenopodes* (the remainning Asthenopodinae) and wider again in *Hubbardipes crenulatus* (Fig. 22A).

The length of male foreleg is another character commonly used to define groups (here represented as the ratio length of FW/foreleg, Fig. 22C): a paulatine shortening of male foreleg in two lineages (Fig. 22C) and female pronotum (in just one of them, Fig. 22D) were obtained in the phylogeny. Both trends are only coupled (i.e., show simultaneous variation in the same node) in the clade including *Priasthenopus gilliesi*, *Povilla* and *Asthenopus* s.s. It is interesting to note that a character commonly used to define the subfamily Asthenopodinae (ring-like prothorax in the male) did not present changes in our phylogeny because of large intraspecific variation. Another character used as defining the subfamily (parallel ICu veins in forewings) is a plesiomorphy (the apomorphic state is present in Campsurinae).

General shape of the forceps in male adults shows opposing tendencies, becoming slender in Campsurinae but stouter in Asthenopodinae (Fig. 21C). The reduction in the number of segments from three in *Ephoron*, to one in the base of Campsurinae + Asthenopodinae is independently reversed twice (in the ancesters of *Tortopus* + *Tortopsis*, and *Povilla* + *Languidipes*). Independent losses involve also to *Languidipes* and Campsurinae in relation to median remnant of styliger plate. A reduction in the length and anastomosis of marginal intercalary veins (reduction of a marginal archedictyon) is present independently in both subfamilies (Campsurinae and Asthenopodinae).

### Geographical comments

Polymitarcyidae is widely distributed (Fig. 23), including tropical and temperate areas, with the exception of Australia and New Zealand (Kluge 2004, McCafferty 2004). Polymitarcyinae (*Ephoron* and *Eopolymitarcys*) is widely distributed in the Holarctic, Ethiopian and Oriental regions, sharing southern portions of its range with selected species of the other subfamilies (Asthenopodinae in Africa, and Campsurinae in North America, Fig. 23). Neotropical Asthenopodinae is not a monophyletic group, because *Asthenopus* s.s. is sister to the Ethiopian-Oriental clade (*Povilla* and *Languidipes*). Campsurinae is mainly a Neotropical group, with only 6% of its species reaching the Nearctic (squares in Fig. 23).

The spatial analysis of vicariance found 6 disjoint sister pairs (Fig. 3): 1) the Neotropical *Asthenopus* (Amazonas and Paraná basins) vs *Povilla* + *Languidipes* (Ethiopian-Oriental) (Fig. 23, barrier 1); 2) *Povilla* in Africa and SE Asia vs *Languidipes* in the Malayan peninsula and the Island of Java (Fig. 23, barrier 2); 3) *Asthenopus guarani* known only from the Parana Basin vs its sister clade (*Asthenopus hubbardi* (*Asthenopus curtus* (*Asthenopus magnus* + *Asthenopus angelae*))) known from the Amazonas and Parana basins (Fig. 24, barrier 3); 4) *Asthenopus hubbardi* in Colombian Amazonas vs (*Asthenopus curtus* (*Asthenopus magnus* + *Asthenopus angelae*)) more widely distributed (Fig. 24, barrier 4); 5) *Asthenopus magnus* in the Ecuadorean Napo vs *Asthenopus angelae* more to the East in the Amazonas and Parana basins (Fig. 24, barrier 5); and 6) *Asthenopodes chumuco* known from three localities in Amazonas lowlands, Guyana and Eastern-Central Brazil (Espirito Santo) vs *Asthenopodes picteti* + *Asthenopodes traverae* known from the Parana basin (Fig. 25, barrier 6).

### Key to the subfamilies and genera of Polymitarcyidae

A key to the subfamilies of Polymitarcyidae is presented because new species described here show some of the characteristics (e.g., many crossveins, numerous anastomosed marginal intercalaries, etc.) previously used to diagnose other subfamilies. A key to the genera is proposed to include new or recently described genera (*Tortopsis*, *Hubbardipes* and *Priasthenopus*) or those raised to generic status (*Languidipes*, in Baumgardner et al. 2012, and *Asthenopodes*, here). The triangullar cell in Cu sector mentioned by Baumgardner et al. (2012) as diagnostic for *Languidipes* is not used in the key because it is also present in some *Asthenopus* females.

Key to the species of *Asthenopodes* and *Asthenopus* are given after each generic section (see below). Please note that in male genitalia, the pedestals are considered part of the styliger plate (Kluge 2004), thus number of forceps segments does not include them.

### Key to adults (imagos and subimagos, except measures and ratios aplicabble only to imagos) and eggs of Polymitarcyidae

**Table:** 

1	FW with vein Sc ending before the tip of the wing, its apical portion not curved posteriorly; cubital area of FW broadly expanded, usually with 3–5 intercalaries and many cross veins and marginal intercalaries; FW with MA fork 1/3 or more distance from wing base (i.e., a long MA stem is present); HW with convex intercalary between R_1_ and Rs field (x.i. in Fig. 16 L); forceps with long basal and 2 slender apical segments; eggs with polar cap of type II (formed by tubular-shaped accumulations of attachment threads)	**Polymitarcyinae (Holarctic, Ethiopian, Oriental)**
–	FW (e.g., Figs 11, 16) with vein Sc ending at the tip of the wing or beyond, its apical portion strongly curved posteriorly; cubital field narrow with two ICus present, MA fork near base of the wing (i.e., MA stem very short); HW of male without convex intercalary between R_1_ and Rs; forceps without short apical segments (e.g., Figs 12, 17); eggs with polar cap absent, single or double, of type III when present (formed by loosely coiled threads)	**2**
2	Pronotum ring-like (ratio width/length ca. 1/3); FW with CuA relatively straight, ICus subparallel (e.g., Figs 11, 16); median remnant of styliger plate present (e.g., Fig. 12E–F), except in *Languidipes*; all female legs present and complete but reduced in size; eggs ovoid, without a concave side, polar cap present at both poles (e.g., Fig. 13A–C) or dettached from the egg (Fig. 18I–K), chorion smooth or with disk-like structures	Asthenopodinae... **3**
–	Pronotum longer (width similar to length), in male with triangular anterior portion; CuA sigmoid, ICus apically diverging; median remnant of styliger plate absent; female forelegs generally absent in imago (may be present in subimago), if complete much reduced in size and twisted; eggs C-shaped, resembling a sphere with one side pushed in, polar cap single if present, chorion punctuated at least in the concave face	Campsurinae (Neotropical, Nearctic)... **8**
3	Eyes of male enlarged, separated on meson of head by a distance subequal to width of lateral ocellus	***Languidipes* Hubbard (Oriental)**
–	Eyes of male normal, similar to female, separated on meson of head by a distance greater than 2 times the width of lateral ocellus	**4**
4	Penes blade-like (Fig. 19D, G); female sternum 8 with anteromedian keel basally swollen (Fig. 19C, E–F); egg without polar caps, chorion mainly smooth but few disk like structures may be present (Fig. 19K)	***Povilla* Navás (6 spp, Ethiopean, Oriental)**
–	Penes variable but commonly cylindrical (Figs 7A, 8A, 12, 17); if an anterior keel is present in female sternum 8, it is not swollen basally (Figs 18F–H); eggs with two polar caps, chorion covered with disk-like structures (Figs 7D, 8K, 13A–C, 18A–E)	Neotropical Asthenopodinae... **5**
5	Penes with many spines on outer subapical margin (Fig. 7A); male foretarsal segment 1 partially fused to tarsal segment 2 (Fig. 20D); female sternum VIII with a protruding subcircular anteromedian structure (figures 12–13 in Molineri et al. 2011); eggs with the space between large plates completely covered by smaller plates (Fig. 7D–E)	***Hubbardipes* gen. n.**
–	Penes with smooth outer margin (Figs 8A–C, 12, 17); male foretarsal segment 1 distinct (Fig. 20E–J) or completely fused with tibia (Fig. 20A–C); female sternum VIII with an anteromedian keel (Figs 13D, 18F–H) or a flat oval structure (Fig. 8J); eggs with the space between plates smooth, if small plates are present, also smooth chorion is present around them (Figs 8K–L, 13A–C, 18A–E)	**6**
6	Foreleg (FL) much shorter in length than FW (ratio FW/FL length = 1.4–2.0), foretarsal segment 1 distinct (Fig. 20E–J), apex of claws not strongly expanded; 0–2 (rarely with a third, weak) crossveins between R and M, basal to R fork (Figs 8D–E, 16); female sternum VIII with a long anteromedian keel (Fig. 18F–H) or with a pair of medium sized sockets (Fig. 8J); polar caps about as wide or wider than egg (Figs 8K, 18C, 18H)	**7**
–	FL subequal in length to FW (ratio FW/FL length: 1.0–1.2); 3–5 crossveins between R and M, basal to R fork; foretarsal segment completely fused with tibia (Fig. 20A–C), apex of claws strongly expanded (Figs 13E–F); female sternum VIII with a short anteromedian keel (Fig. 13D); polar caps usually smaller, not as wide as egg (Fig. 13B–C), except in *picteti*, with a cap subequal in width to egg (Fig. 13A)	***Asthenopodes*** (3 spp, Neotropical)
7	Penes with a short apical spine-like projection (arrow in Fig. 17C, D, F); female sternum VIII with a long and thin anteromedian keel (Fig. 18F–H) and very reduced sockets; eggs with polar caps formed by 3–8 tightly twisted threads (Fig. 18A–E)	***Asthenopus*** (5 spp, Neotropical)
–	Penes apically rounded or slightly pointed but never with a spine (Fig. 8A–C); female sternum VIII with a short blunt keel with larger sockets (Fig. 8J); eggs with polar caps formed by 14–16 tightly twisted threads (Fig. 8K)	***Priasthenopus*** (only *Priasthenopus gilliesi*, Neotropical)
8	Legs II and III flap-like without tibia and tarsi, in both sexes; forceps 1-segmented; eggs with one polar cap (some species show the cap on the convex face)	***Campsurus* Eaton** (45 spp, Neotropical, except 1 Nearctic species)
–	Legs II and III weak and twisted, but complete; forceps 2-segmented (a weak line separates a short basal segment); eggs without polar caps (a long thread coiled around the egg is present in one species, Molineri 2010)	**9**
9	Male sternum IX not divided medially along its length; penes fused basally; pedestal with relatively short parastylus; female FW with IRs complete; sockets on sternum VIII small and submedian	***Tortopus* Needham & Murphy** (7 spp, Neotropical, 1 reaches southern Texas)
–	Male sternum IX divided medially along its length; penes completely separated; pedestal with long and curved parastylus; female FW with IRs incomplete (2 IR veins are wanting); sockets on sternum VIII larger and sublateral	***Tortopsis* Molineri** (8 Neotropical and 2 Nearctic species)

### Nymphs (modified from Molineri 2010)

**Table:** 

1	Outer edge of mandibular tusks with many large tubercles; tarsus and tibia of foreleg separated	**Polymitarcyinae**
–	Outer edge of tusks without large tubercles (Figs 5B, 9C–F, 14A–C) (*Povilla* presents a median indentation, Fig. 14D, H) but small blunt tubercles may be present; tarsus and tibia of foreleg fused (a fusion line with a row of setae may be distinguishable or not, Figs 6A, 10A, 15A)	**2**
2	Dorsum of head mostly glabrous, without large tufts of tightly grouped short setae (Figs 5A, 9A–B, 14I–J); occiput roundly convex; apex of left mandibular tusk with 3–4 pointed processes (Figs 5B, 9C–F, 14A–C)	Asthenopodinae... **3**
–	Dorsum of head with dense patches of short setae, mainly anteriorly to lateral ocelli; occiput flat, subquadrate in dorsal view; apex of left mandibular tusk with 1 pointed process	Campsurinae... **7**
3	Mandibular tusks relatively long and slender, without tubercles or large spines on inner margin (Figs 5B, D)	***Hubbardipes***
–	Mandibular tusks robust, stout (Figs 9C–F, 14A–C), with one or more tubercles on inner margin (arrow in Figs 9D and 14F)	**4**
4	Mandibular tusks without large subbasal tubercle on inner margin; foretarsal claw with double row of denticles (Fig. 10B)	***Asthenopodes***
–	Mandibular tusks with a large subbasal tubercle on inner margin (arrows in Fig. 14G); foretarsal claw with single row of denticles (Fig. 15G)	**5**
5	Apex of left mandibular tusk with 4 pointed processes (Ethiopian and Oriental)	**6**
–	Apex of left mandibular tusk with 3 pointed processes (Fig. 14A–C) (Neotropical)	***Asthenopus*** (3 spp known as nymphs)
6	Outer margin of mandible with a tooth-like indentation (Fig. 14H); abdominal gill I bifid	***Povilla***
–	Outer margin of mandible smooth; abdominal gill I uniramous	***Languidipes***
7	Mandibular tusks with prominent basal or sub-basal tubercle on median margin (rarely tubercle absent), from some to many apical crenulations, numerous setae on outer margin of mandibles; abdominal gill I bifurcated	***Campsurus***
–	Mandibular tusks with 1 or 2 prominent tubercles on distal third of median margin, few long setae on outer margin of mandibles; abdominal gill I single	**8**
8	Mandibular tusks with 2 tubercles (submedian and subapical) on median margin; distal projection of foretibia-tarsus 2/5 the length of claw	***Tortopus***
–	Mandibular tusks with a single subapical tubercle on median margin; distal projection of foretibia-tarsus 2/3 the length of claw	***Tortopsis***

## Discussion

### Taxonomy and phylogeny

Lestage (1922) reported a concise summary of the meandering story of the early taxonomic stages of *Asthenopus*: *Asthenopus* was erected by Eaton (1871) for the species *Asthenopus curtus* (Hagen) and also for *Asthenopus dorsalis* (Burmeister). Later Eaton (1883), without mentioning *Asthenopus*, treated these species at *Campsurus*. *Asthenopus* is not mentioned in the systematic literature until Ulmer (1921) reinstated it as a valid name for *Asthenopus albicans* Pictet (nec Percheron), *Asthenopus curtus* (Hagen) and *Asthenopus amazonicus* (Hagen 1888), but treating *Asthenopus dorsalis* as a *Campsurus*.

Shortly after, Ulmer (1924) erected *Asthenopodes* for *Asthenopus albicans*. Ulmer’s consideration of *Asthenopus albicans* as a species authored by Pictet was an error (Hubbard 1975), and it was renamed by this last author as *Asthenopodes picteti* Hubbard. In 1978 Berner synonymyzed *Asthenopus curtus* with *Asthenopus amazonicus*. Thus, Neotropical Asthenopodinae was at that moment known by two species: *Asthenopus curtus* (Hagen) and *Asthenopodes picteti* Hubbard. Domínguez (1988) described *Asthenopus gilliesi*, a species showing some characters shared by *Asthenopus* and *Asthenopodes* and based on this Hubbard and Domínguez (1988) combined both genera. More recently, two species were added to *Asthenopus*: *Asthenopus crenulatus* Molineri, Cruz & Emmerich (2011) and *Asthenopus angelae* de Souza & Molineri (2012).

Ulmer (1942) discussed many characters to differentiate *Asthenopus* from *Campsurus*, most of them measures and ratios of different structures (prothorax, forewing veins, legs, forceps). In contrast, Ulmer (1942) found only two characters to distinguish *Asthenopus* from *Povilla*, an African Asthenopodinae described by Navás (1912): 1) ICu_2_ free at base or joined by crossveins to nearby veins in *Asthenopus*, but ICu_2_ springing from CuP in *Povilla*; and 2) penis lobes curved and cylindrical in *Asthenopus* but straight and rod-shaped in *Povilla*. The first character is present in *Povilla adusta* but not in the other species of *Povilla* (including *Languidipes corporaali*), so it is not useful at the generic level. The second, may still be used to distinguish both genera, but it is not useful to separate *Hubbardipes* gen. n. from *Povilla* (both showing blade-like penes).

Domínguez (1988) presented a more detailed description concerning these genera, and reported that *Povilla* differs from *Asthenopus* because the former shows: 1) forceps 2-segmented (1-segmented in *Asthenopus*, note that here the genitalia was reinterpreted following Kluge 2004 and the basal segment or pedestal is considered part of the styliger plate), 2) penes bladelike (cylindrical in *Asthenopus*), 3) bifurcation Rs from base to margin: 1/10 (2–2.5/10 in *Asthenopus*), and 4) MP_2_ shorter than IMP (subequal in *Asthenopus*).

Kluge (2004) did not differentiate both genera, apparently treating them as synonyms. But he summarized the features traditionally associated with the subfamily Asthenopodinae (at that time = *Asthenopus* + *Povilla*), distinguishing two “synapomorphies” in the nymph: mandibular tusks specialized in biting (not long, very thick and stout, with serrate inner margin), and foretarsal claw with a row of denticles. Kluge (2004) also listed the following “plesiomorphies” shared by this group: 1) FW with CuA not so strongly curved as in Campsurinae, thus both ICu veins go nearly parallel to basitornal margin and terminate near or anteriad to tornus; 2) genitals retain small median remnant of styliger which is articulated to posterior margin of sternite IX and bears immobile pedestals (narrow basally, and with muscles that move forceps); 3) penial arms retain lateral articulations with postero-lateral angles of tergite IX; 4) imaginal moult is present in both sexes (not in female Polymitarcyinae); 5) egg ellipsoid as usual, two polar caps or none; 6) nymphal gill I bilamellate. Note that a formal quantitative phylogeny was not presented by Kluge (2004), so his use of “synapomorphy” and “plesiomorphy” are *ad hoc* hypotheses.

The inclusion of many of these characters in a formal cladistic analysis permitted us to recognize apomorphic from plesiomorphic states, showing that some of the hypothesized synapomorphies for Asthenopodinae (or particular genera) were not homogeneous in the corresponding clade (e.g., some members of Asthenopodinae may show Campsurinae features, etc.). For this reason, a list of the synapomorphies for each genus is given either in the descriptions and taxon discussion (for *Hubbardipes*, *Priasthenopus*, *Asthenopus* and *Asthenopodes*) or in Appendix 2 (for Asthenopodinae, and the other genera).

The revalidation of *Asthenopodes* is in general coincident with the observations of previous authors (i.e., the character changes defining the group are those previously reported by Ulmer 1924, Traver 1956 and Hubbard 1975), and new characters are added to its diagnosis due to the knowledge of the egg and nymph. The generic status of *Hubbardipes* and *Priasthenopus* are supported by many autapomorphic character states and its position in the phylogeny is intermediate between *Asthenopodes* and *Asthenopus*. That the Neotropical species do not form a monophyletic group is a novel hypothesis obtained in our study.

### Biogeography

Polymitarcyidae probably originated in Pangea and with the break up of the supercontinent in Laurasia and Gondwana, the first division of the family occurred. The subfamily Polymitarcyinae diferentiated as a Laurasian group (but this would require the ad hoc hypothesis of a later expansion to Africa) (triangles in Fig. 23), indicating a vicariant pattern with the ancestor of Asthenopodinae + Campsurinae, a Gondwanic group. This Gondwanic group probably was always restricted to temperate or warm climates because it is absent from Southern South America, Australia and New Zealand. Asthenopodinae still shows this pattern but Campsurinae extended its range (originally Neotropical) to the Nearctic region but marginally (only 4 of the 62 species, Fig. 23). The ancestor of Campsurinae and Asthenopodinae was most probably distributed in tropical-subtropical Gondwana, where it originated at least five groups: 1) Campsurinae, 2) *Asthenopodes*, 3) *Hubbardipes*, 4) *Priasthenopus*, and 5) the ancestor of *Asthenopus* + *Povilla* + *Languidipes*. Subsequently, the formation of the Atlantic Ocean separated the South American *Asthenopus* from the Ethiopean-Oriental group (ancestor of *Povilla* + *Languidipes*).

Two fossils may falsify these hypotheses. *Mesopalingea* Whalley & Jarzembowski (a Campsurinae sensu McCafferty 2004) is known in the nymphal stage from the late Jurasic-early Cretaceus of Spain. Kluge (2004) classifies it as Fossoria insertae sedis (a group including almost all the burrowing families), thus not recognizing this genus as a Polymitarcyidae. The tusks of *Mesopalingea* strongly resemble those of *Campsurus*, but we suspect that this similarity is only superficial because at least two features would place the fossil outside Campsurinae: a somewhat prominent frontal projection and a very short ring-like prothorax.

The second fossil with contradictory information, *Asthenopodichnium* Thenius (1979) is known from 2 trace species (tubular marks in wood and bones) from the Miocene of Vienna. These tubular marks were atributed to Polymitarcyinae by Thenius (1979, 1988) or Asthenopodinae by McCafferty (2004). There is no evidence that they were produced by a mayfly; actually other animal taxa produce similar traces, so we agree with Kluge’s (2004) treatment of *Asthenopodichnium* as Animalia insertae sedis.

Five groups (Campsurinae, *Asthenopodes*, *Hubbardipes*, *Priasthenopus* and *Asthenopus*) constitute independent sources to study biogeographic patterns inside the Neotropical region. Vicariant patterns in Campsurinae are only known for *Campsurus* (Molineri & Salles, 2013), which can be compared to those here indicated by *Asthenopodes* and *Asthenopus* (*Hubbardipes* and *Priasthenopus* are monotipic). *Asthenopodes* shows a single disjoint sister pair (*Asthenopodes chumuco* in the Amazonas subregion vs *Asthenopodes traverae* + *Asthenopus picteti* in the Parana subregion, barrier 6 in Fig. 25), coincident with the disjunction between *Asthenopus guarani* vs the remaining species of *Asthenopus* (barrier 3 in Fig. 24). This pattern resembles that of *Campsurus amapaensis* vs *Campsurus argentinus* + *Campsurus major* (Molineri & Salles, 2013) and was probably initiated by the appearance of drier areas (Chacoan biogeographic subregion) separating Amazonas subregion to the North from the Paranaense subregion in the SE (Morrone 2001). The two more recent vicariant events involve species in *Asthenopus*: barrier 4 separating *Asthenopus hubbardi* from *Asthenopus curtus* + *Asthenopus angelae* + *Asthenopus magnus* (detail in Fig. 24) and barrier 5 with *Asthenopus magnus* in the Napo Region vs *Asthenopus angelae* more to the East, Fig. 24). Without a molecular dating of these clades we can only speculate possible explanations related with pleistocene refugia (Hooghiemstra and van der Hammen 1998).

But perhaps the most interesting biogeographic pattern found in the present work is the paralogy in tropical South American areas caused by the paraphyletic nature of the Neotropical Asthenopodinae. The accepted hypothesis of vicariance between South American and Afro-Oriental Asthenopodinae was the formation of the Atlantic Ocean during the breakup of Gondwana (Lomolino et al. 2006). But as this event was found here to be more recent than those involving the more basal clades (*Asthenopodes*, *Hubbardipes* and *Priasthenopus*), a more complex taxon history of the group is suggested. As these events (generic and suprageneric divergences) are deeper than the divergence of sister species in *Asthenopus* mentioned above, marine transgressions (>10 millions years, Ortiz-Jaureguizar and Cladera 2006) become possible scenarios.

## Descriptions

### Hubbardipes gen. n.

Taxon classificationAnimaliaEphemeropteraPolymitarcyidae

http://zoobank.org/7A588525-40FB-41B1-976F-1E915BAF813F

**Figure 4.**
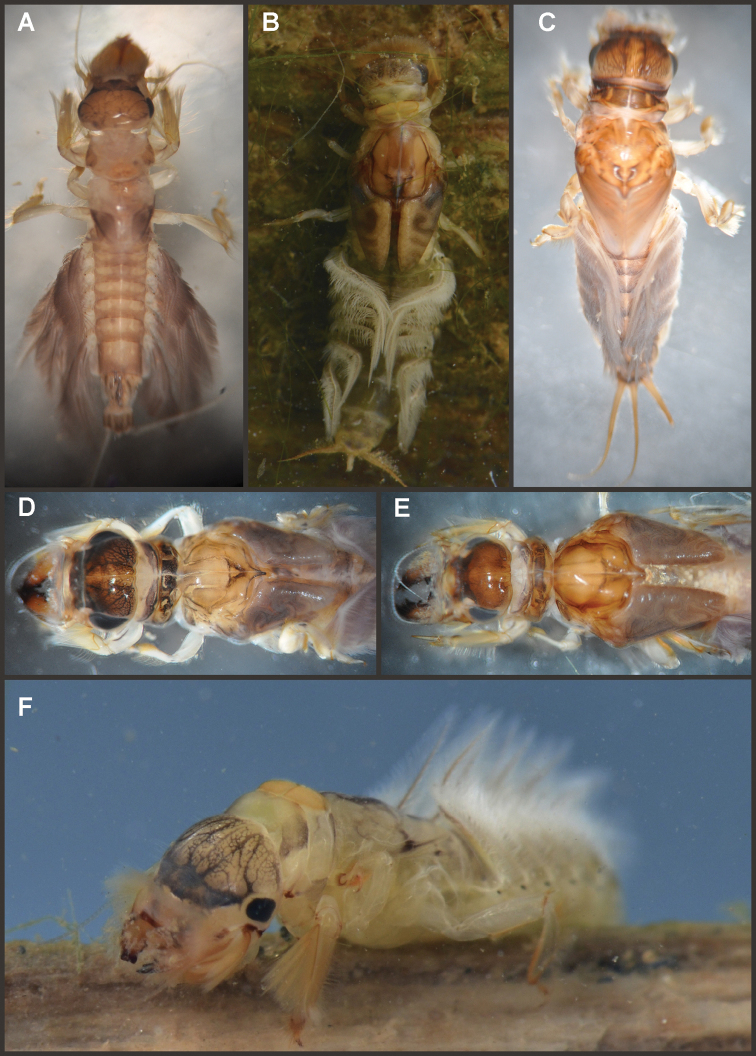
Asthenopodinae nymphs: **A** *Hubbardipes crenulatus* **B** *Asthenopodes chumuco* **C** *Asthenopus magnus* **D** *Asthenopus angelae* **E** *Asthenopus curtus* **F** *Asthenopodes chumuco*.

**Figure 5.**
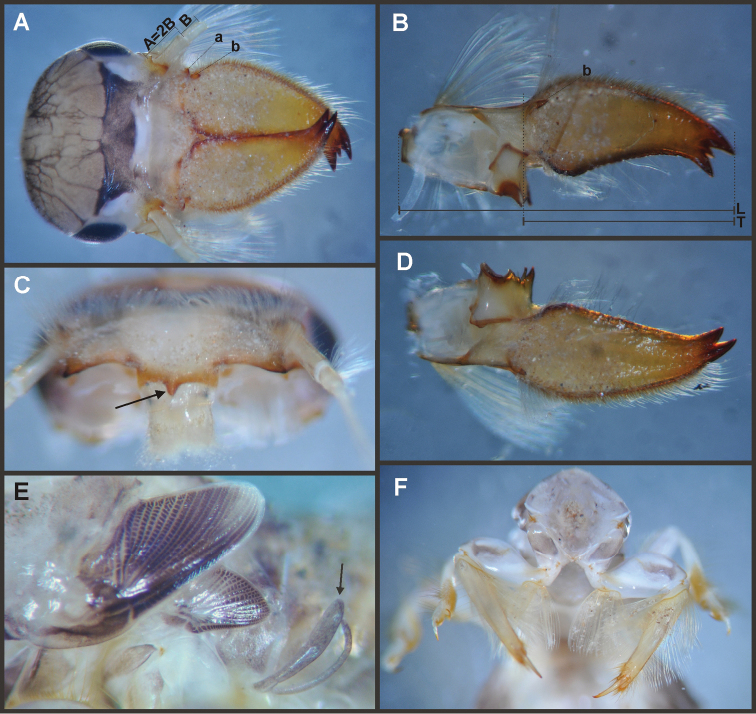
*Hubbardipes crenulatus*, nymph. **A** head, d.v. **B** left mandible, d.v. **C** head, frontal view (arrow indicates frontal anteromedian projection) **D** right mandible, d.v. **E** wingbuds and gill I **F** frontal view of thorax (head removed).

**Figure 6.**
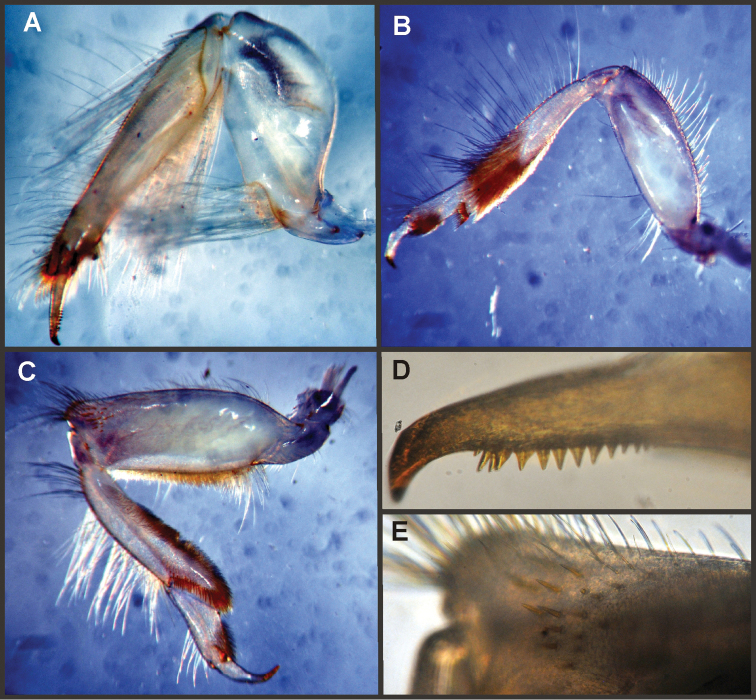
*Hubbardipes crenulatus*, nymph. **A** foreleg, d.v. (functionally frontal) **B** middle leg, d.v. **C** hind leg, v.v. (functionally dorsal) **D** foretarsal claw, detail **E** detail of apical spines on hind femur.

**Figure 7.**
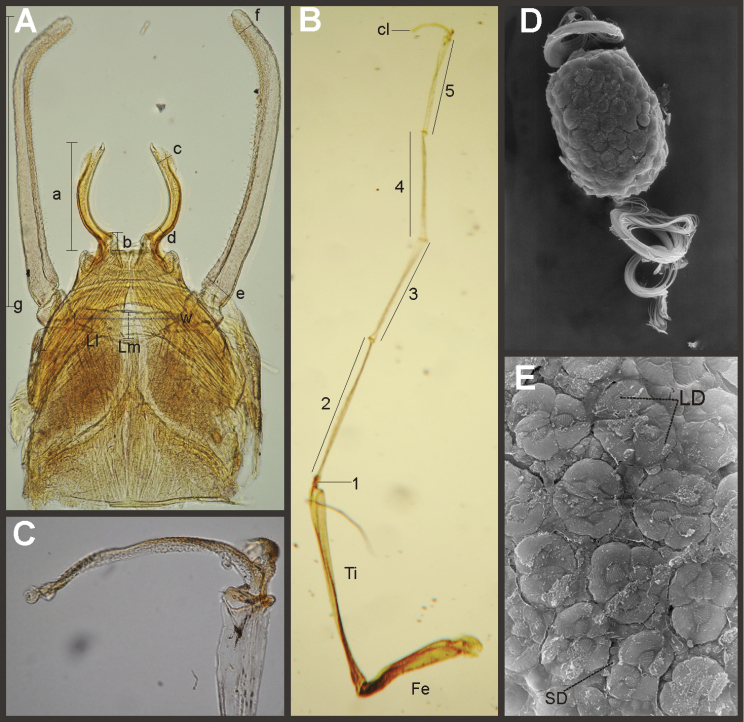
*Hubbardipes crenulatus*, male imago and egg: **A** male genitalia, v.v. **B** male foreleg **C** detail of foretarsal claw **D** egg, general view **E** detail of chorion (LD = large disks).

Figs 4A , 5 , 6 , 7 , 20D

Asthenopus (partim) Molineri et al. 2011: 34.

#### Type species.

*Asthenopus crenulatus* Molineri, Cruz & Emmerich, 2011 (original designation).

#### Species included.

*Hubbardipes crenulatus* (Molineri, Cruz & Emmerich) comb. n.

#### Diagnosis.

Eight autapomorphies define the genus *Hubbardipes* in our cladistic analysis (Appendix 2), some of them are: 1) male foretibia very wide apically (Fig. 20D), first tarsal segment partially fused with second tarsal segment (Fig. 7B); 2) thumb (inner basal projection) of penes reduced, indistinguishable (Fig. 7A); 3) apex of penis lobe wider than base and with many marginal spines (Fig. 7A); and 4) nymphal mandibular tusk with smooth to slightly crenulated inner margin, without submedian tubercles (Figs 5B, D). Additionally, the following combination of characters is useful to distinguish *Hubbardipes* from other genera in Polymitarcyidae: 1) ratio length male FW/foreleg = 1.1; 2) male foreleg with large and apically widening tibia, tarsal segment 1 small and partially fused to tarsal segment 2, tarsal claws slender slightly wider at apex; 3) pronotum width/length ratio: 1.5 (male), 2.2 (female); 4) marginal intercalary veins present on the entire margin of fore and hind wings, generally shorter than distance between longitudinal veins in male, but longer and anastomosed in female; 5) in both sexes FW with 3–4 crossveins between R and M, basally to R fork; 6) basal relation of FW veins IMP-MP_1_ variable (IMP joined to MP_1_, or basally free), MP_2_ curved toward CuA and fused to CuA and MP_1_ by cross veins (forming a characteristic oblique Y); 7) median remnant of styliger plate subquadrate and small, pedestals short also subquadrate and relatively small; 8) forceps slender, ratio length/basal width = 8.5–9.0 (Fig. 7A); 9) penes relatively thin, with many spines near the apex on outer edge, slightly curved inward (Fig. 7A); 10) female abdominal sternum VIII with anteromedian paired sockets on a protruding subcircular structure; 11) eggs with relatively large polar caps (almost as wide as egg, ratio width egg/cap 1.2–1.4), each cap formed by 4–7 threads, chorion completely covered by large disk-like plates and smaller irregular plates (Figs 7D–E); 12) nymphal head with a small median projection on the frons (arrow in Fig. 5C); 13) nymphs with long robust tusks, without inner tubercles, with 2 or 3-pointed apex (right and left mandible respectively) (Figs 5A–B, D); 14) nymphal foretarsal claw with single row of about 14 denticles, denticles are small basally, and larger medially, 3 denticles are closer together near the apex, and the last one is much smaller (Fig. 6D); 15) nymphal dorsal apex of hind femur with ca. 20 stout spines (Fig. 6E).

Male imago. Length (mm): body, 7.0–7.8; FW, 7.5–8.6; HW, 3.1–3.7; foreleg, 6.2–6.9; cerci, 21.6. Antennae: scape slightly longer than pedicel; flagellum bristle-like. Thorax. Pronotum width/length: 1.5–2.5. Legs. Forelegs relatively long, ratio length FW/foreleg = 1.1; tarsal segment 1 fused to tarsal segment 2 (Fig. 20D), longest segment is tibia, ratio length tarsal segment 2/tibia = 0.8; tarsal segments long decreasing in length from 2>3>4>5 (Fig. 7B); claws different in size, one long the other short, not strongly widened distally (Fig. 7C). Wings. FW with 14 marginal intercalaries along hind margin, also present along entire hind margin of HW; these intercalaries present relatively numerous connections with other cross and longitudinal veins but they are not very anastomosed; 3–4 crossveins between R and M sectors basally to R fork; Rs stem length/Rs from fork to margin = 0.24; ratio MA from fork to margin/stem length = 9–12; IMP fused basally to MP_1_; MP_2_ fused to CuA. Genitalia (Fig. 7A): median remnant of styliger plate present, and with pedestals short and subquadrate with inner apical corner slightly protruding distally; forceps relatively long and slender, ratio length/basal-width = 9. Terminal filament reduced, cerci long (ratio length FW/cercus = 0.35).

Female adult. Length (mm): body, 10.2–10.8; FW, 11.1; HW, 4.3. Thorax. Pronotum width/length = 1.5–2.3. Wings with more crossveins and intercalaries than male. Abdominal sternum VIII with paired anteromedian sockets on an oval and ventrally protruding structure, sockets small, shallow and contiguous. Terminal filament reduced, shorter than tergum VIII, with few thin annuli; cercus 0.5–0.6 times the length of abdomen.

Eggs (Fig. 7D–E). Length, 221–266 μ; width, 143–152 μ. Oval (ratio maximum length / maximum width = 1.5–1.7), with two medium sized polar caps on apices (ratio maximum with of egg/maximum width of coiled polar cap = 1.2–1.4), each cap formed by 4–7 very long filaments. Chorionic surface completely covered by plates: large disk-like structures frequently 3-partited with a fine microsculpture forming a dashed pattern, and many small (and irregular in shape) plates covering completely the spaces between the large plates.

Nymphs (Fig. 4A). Length (mm): body, 11.0–14.5; cerci, 4.5–5.5; terminal filament, 6.0. Head (Figs 5A, C) subquadrate in dorsal view, smooth (without pilose area), antennae 1.7–2.0 times length of head. Occipital region well developed, convex (Fig. 5A). Head capsule dorsally projected at bases of antennae. Frontal ridge marked only by a dense transversal row of setae; frons acutely projected medially (Fig. 5C); clypeus and labrum membranous and small, labrum densely covered with long setae on dorsum. Mandibular tusks (Figs 5A, B, D) relatively long and slender, similar in length to head capsule, dorso-ventrally flattened, left tusk apically with 3 tubercles (the median is reduced in length), right tusk with 2 tubercles; dorsal surface of tusks wide, with crenulated inner margin bearing long setae; outer margin with a small dorsal tubercle near base (“b” in Figs 5A–B) and densely covered with stout setae along entire margin; the small basal tubercle (“b”) forms an additional articulation between mandible and head capsule (“a” in Fig. 5A). Body of mandible: molae and canines present but small, margin between them sharp-edged (acutely protruding in right mandible, Fig. 5D); with basal U-row of long filtering setae in both mandibles. Thorax. Pronotum with short anterior ring (collar), 1/3 the length of posterior ring (length taken at the medio-longitudinal line), anterolateral corners projected, spine-like. Legs (Fig. 6). Leg I (Fig. 6A): femora very wide, well developed, with a double ventro-basal row of long filtering setae; tibio-tarsus (fused) with 3 rows of filtering setae (2 on anterior face and 1 on inner margin), roundly projected apically; tarsal claw long and slender with a row of marginal denticles (Fig. 6D). Leg II (Fig. 6B): smaller, with thinner femora, with scattered long setae, mostly basally and along hind margin; tibia and tarsi with row of long setae on outer (dorsal) margin, ventrally with many stout spines on apical half, with a distal brush of thick setae; tarsal claw weaker, without denticles. Leg III (Fig. 6C): as leg II except larger and with anterior margin of femur densely covered with thick setae, femur distally with a group of acute stout spines (Fig. 6E), tibia without distal brush. Coxae I and II directed ventrally, coxae III directed laterally. Abdomen. Sternite I stronger and partially fused with metasternum. Gill I reduced in size, dark gray, double, both portions of a similar length, but the dorsal is wider (arrow in Fig. 5E). Gills II–VII well developed, ventral portion smaller than dorsal portion. Tergum X with short and blunt, poorly developed posterolateral spine. Cerci slightly shorter than terminal filament, with long setae at joinings.

#### Etymology.

*Hubbardipes* from “Hubbard” and “pes”, Latin, masculine, meaning “foot”. We dedicate the genus to Mike Hubbard, mayfly specialist, who devoted many of his works to the Polymitarcyids.

#### Distribution.

Amazonas subregion (Amazonas river in Colombia and Brazil).

#### Discussion.

*Hubbardipes* was recovered as sister to a larger clade containing *Priasthenopus*, *Asthenopus*, *Povilla* and *Languidipes* (see synapomorphies in Appendix 2), not related with *Asthenopodes*, as previously thought (Molineri et al. 2011). *Hubbardipes* shows many differences in the adult stage and, more markedly in the nymph and egg here described for the first time. Male genitalia is unique in form and structure (Molineri et al. 2011), nymphal tusks and microsculpture on disk-like structures of the egg are exceptional as well.

### Hubbardipes crenulatus

Taxon classificationAnimaliaEphemeropteraPolymitarcyidae

(Molineri, Cruz & Emmerich) comb. n.

Asthenopus crenulatus Molineri, Cruz & Emmerich, 2011: 34.

#### Material.

Listed in Molineri et al. (2011), from Brazil (Amazonas, Presidente Figueiredo) and Colombia (Amazonas, Leticia, Reserva Natural Palmarí). Additional material: 2 male subimagos and 3 nymphs from BRAZIL: Amazonas, Tefé, São João do Catuaí, Igarapé Jutaí (A07), S 3°41'52.8" - W 64°9'18", 12.ix.2003, luz UV1, 067 FCM (CZNC); 7 nymphs from COLOMBIA, Dpto. Amazonas, rio Yavari junction Orejon, S 4°7'12" - W 69°55'43", E. Domínguez & N. Torres col. (IBN).

#### Diagnosis.

*Hubbardipes crenulatus* (Molineri et al., 2011) comb. n. is known from adults of both sexes, eggs and nymphs, and for the moment it is the only known species in the genus. The characters useful to distinguish it from other Asthenopodinae are listed in the generic diagnosis.

Nymphs (Fig. 4A). Length (mm): body, 11.0–14.5; cerci, 4.5–5.5; terminal filament, 6.0. General coloration whitish light brown. Head with a gray band between lateral ocelli and fine netting pattern on occiput (Fig. 5A). Antennae: scape bare, long and slender, pedicel shorter with many dorsal setae, flagellum bare with numerous annuli increasing in length distally. Thorax. Pronotum shaded black on anterior ring and more slightly shaded witn brownish-gray on posterior ring except on a pair of submedian longitudinal pale lines. Meso- and metanotum shaded widely with gray, with dark gray wingbuds, developing veins paler (Fig. 5E). Legs (Fig. 6). All coxae and trochanters shaded with gray, femur I shaded with gray, remaining segments and legs II and III whitish-yellow; foretarsal claw with ca. 20 denticles increasing in size distally. Abdomen. Terga more or less uniformly shaded brownish-gray, except on pale transverse dashes laterally, and pale subcircular submedian marks; tergum X with two submedian pale bands; sterna paler than terga, shaded with gray very slightly, somewhat darker on sterna IX–X. Gill I dark gray, gills II–VII brownish gray, ventral portion paler than dorsal portion. Caudal filaments whitish.

#### Distribution.

Amazonas river, from Leticia (Colombia) to Manaus (Brazil).

#### Discussion.

*Hubbardipes crenulatus* (Molineri et al., 2011) comb. n. was recently described from male and female adults in the genus *Asthenopus*, with the knowledge of the nymphs and based on the results of the phylogenetic analysis, it became evident that this species pertain to a distinct group, that we propose here as a new genus.

### Priasthenopus gen. n.

Taxon classificationAnimaliaEphemeropteraPolymitarcyidae

http://zoobank.org/6EDB4C4E-8A74-47F6-9B8A-E7955DFADA2E

Figs 8 , 20E

Asthenopus (partim) Domínguez 1988: 21; Hubbard and Domínguez 1988: 207.

#### Type species.

*Asthenopus gilliesi* Dominguez, 1988 (original designation).

#### Species included.

*Priasthenopus gilliesi* (Dominguez), 1988 comb. n.

#### Diagnosis.

**Figure 8.**
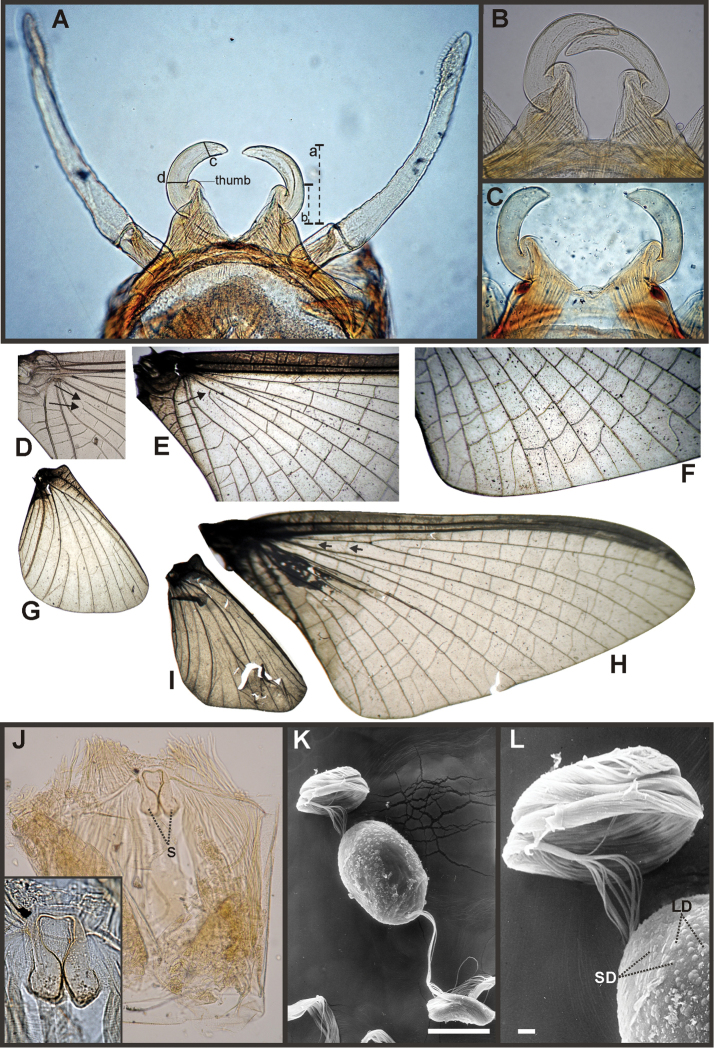
*Priasthenopus gilliesi*, adults and egg. Male imago: **A** genitalia, v.v. (Colombian specimen) **B** penes (Bolivian specimen) **C** penes (Uruguayan paratype) **D–E** FW detail of base (arrows indicate veins IMP and MP2) **F** detail of FW, posterior margin **G** hind wing. Female subimago: **H** forewing **I** hind wing **J** sternum VIII and detail of sockets (s). Egg (SEM): **K** general view **L** detail of cap and chorion, LD = large disks, SD = small disks.

**Figure 9.**
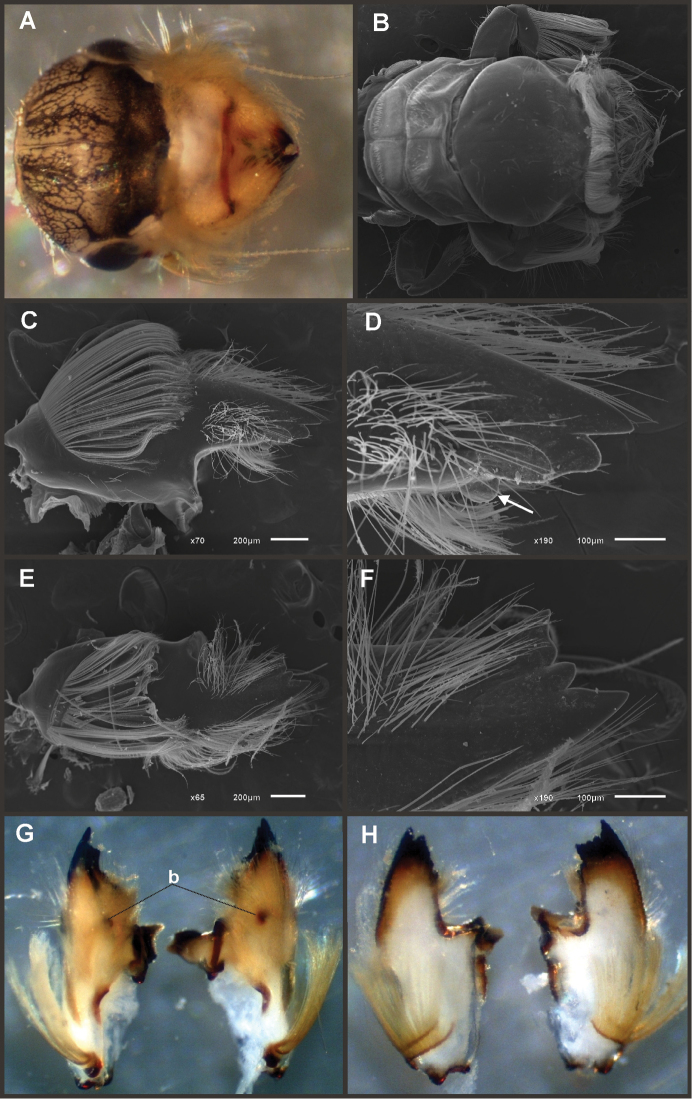
*Asthenopodes* nymph. *Asthenopodes chumuco*: **A** head, dorsal view **B** head and pronotum, d.v. **C–D** right mandible and detail of apex, v.v. (arrow = subdistal tubercle) **E–F** left mandible and detail of apex, v.v. **G** mandibles, d.v. (b = dorsal tubercle) **H** same, v.v. **A, G, H** stereomicroscope photographs **B–F** SEM.

**Figure 10.**
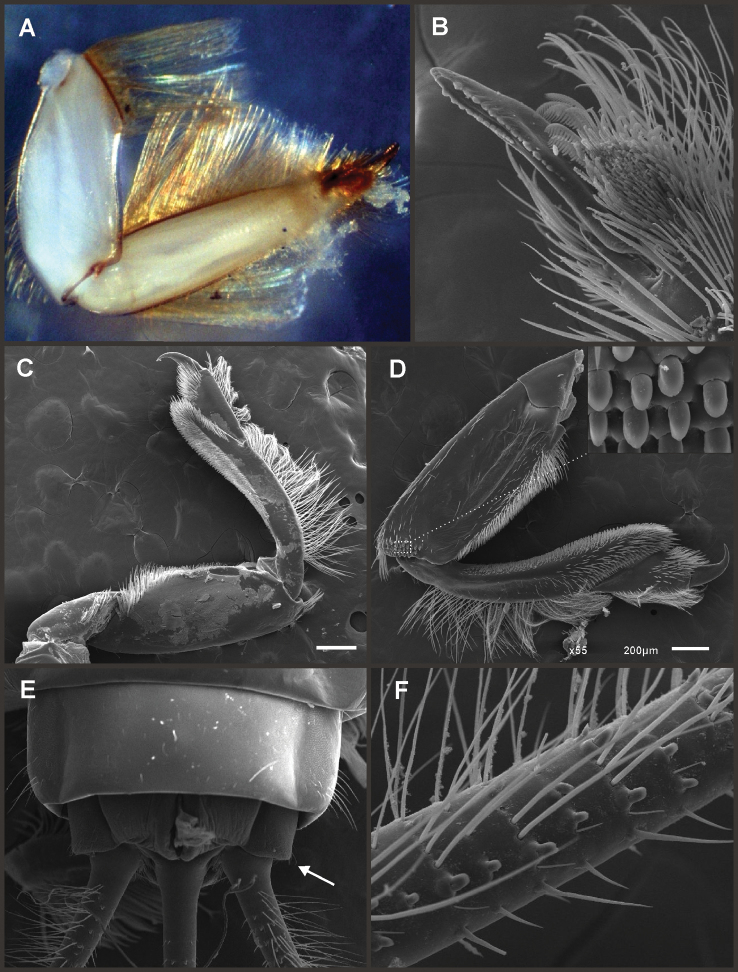
*Asthenopodes* nymph. *Asthenopodes chumuco*: **A** foreleg, v.v. **B** detail of foretarsal claw **C** middle leg, d.v. **D** hind leg, d.v. with detail of femoral apex **E** abdominal sterna IX–X (arrow indicate absence of spine on paraproct) **F** detail of cercus. All figs from SEM except A from stereomicroscope.

**Figure 11.**
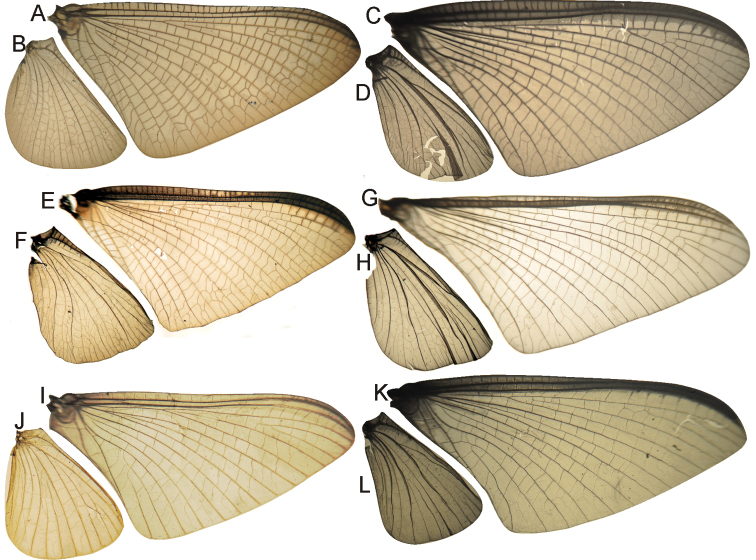
*Asthenopodes* wings, steromicroscope photographs. Males to the left, females to the right: **A–D** *Asthenopus picteti* **E–H** *Asthenopodes traverae* **I–L** *Asthenopodes chumuco*. Not to scale.

*Priasthenopus* gen. n. presents seven autapomorphies in our cladistic analysis (Appendix 2), six are variations in continuous characters (e.g., stoutter penis lobe and larger thumb) and the seventh is the presence of a short closed cleft between the aforementioned structures. This genus can be distinguished from the other by the following combination of characters: 1) ratio length male FW/foreleg = 1.6–2.0; 2) first tarsal segment subquadrate not fused with tibia (Fig. 20E); 3) pronotum width/length ratio: 2.1–2.5 (male), 2.6 (female); 4) 5–10 marginal intercalary veins present on the margin of FW (Figs 8F, H), about as long as the distance between longitudinal veins in male, slightly longer in female, HW without marginal intercalaries; 5) male FW with 1 cross vein between Rs and MA basal to Rs fork (2 in female); 6) vein MP_1_ basally free (types from Uruguay and specimens from Colombia, Fig. 8D) or tending to fuse, although not completely, with MP_2_ (in specimens from Bolivia, Fig. 8E), IMP basally free; 7) median remnant of styliger plate rectangular thin and convex posteriorly, pedestals rectangular (Fig. 8A); 8) ratio total length/basal width of forceps 6.3–7.3 (Fig. 8A); 9) penes relatively short with a similar width along their length, strongly curved, without apical spine or spine very slightly marked as a subapical indentation, thumb rounded (Figs 8A–C); 10) female sternum VIII with well distinguishable anteromedian sockets (Figs 8J); 11) eggs with relatively large polar caps formed by 14–16 long threads (Fig. 8K), chorion loosely covered by medium-sized and small circular plates (Fig. 8L).

Male imago. Length (mm): body, 5.0–8.0; forewing, 5.7–8.4; hind wing, 2.6–3.9; foreleg, 3.1–4.5; cerci, 19.0–25.0. Pronotum width/length: 2.1–2.5. Wings (Figs 8D–G). FW with 5–10 marginal intercalary veins (Fig. 8F), each imv is about as long as the distance between corresponding longitudinal veins; vein MP2 basally free (Fig. 8D) or base directed towards MP1 (but not completely fused with it, Fig. 8E); IMP basally free; 1 cross vein present between Rs and MA basal to Rs fork. HW without marginal intercalaries (Fig. 8G). Legs. Forelegs about half the length of FW, middle and hind legs reduced in length and poorly sclerotized but with all the segments present and distinguishable. Genitalia (Figs 8A–C): median remnant of styliger plate rectangular, thin and convex posteriorly, pedestals rectangular and relatively small (Fig. 8A); 1-segmented forceps, ratio total length/basal width 6.3–7.3 (Fig. 8A); penes (Figs 8A–C) relatively short with a similar width along their length, strongly curved medially, and without apical spine. Cerci long and well developed, terminal filament extremely reduced as common in the family.

Female adult. Length (mm): body, 6.5; FW, 8.9; HW, 3.3; cerci, 1.5. Pronotum ring-like. Wing venation (Figs 8H–I) similar to male except FW with 2 cross vein between Rs and MA basal to Rs fork (arrows in Fig. 8H). Abdominal sternum 8 with anteromedian pair of small sockets (Fig. 8J). Cerci short, about 0.2 the length of FW.

Eggs (Figs 8K–L). Length, 240–275 µ; width, 150–165 µ. Two large polar caps (maximum width, 140–185 µ), formed by 14–16 very long coiled threads. The caps are as wide as or wider than the egg. Chorionic surface with medium sized and small subcircular disks.

#### Etymology.

Arbitrary combination of letters.

#### Discussion.

Treating this sole species in a new genus, distinct from *Asthenopus* is justified by its phylogenetic position (sister to the clade *Povilla*-*Asthenopus*). The other possibility to fit taxonomy to phylogeny would be to synonymize the entire clade (including *Povilla* and *Languidipes* besides *gilliesi*) in *Asthenopus*. This is the scheme apparently presented by Kluge (2004) but this seems inadequate to us because of relatively large morphological gaps between the groups (including characters from eggs, nymphs and adults of both sexes). Furthermore, we tested *Priasthenopus gilliesi* position considering the hypotethical situation that its nymphs (still unknown) be identical to *Asthenopus* s.s., since male genitalia of both groups are similar. Even so, there were no changes in the resulting tree. The description of female adults and eggs is an original contribution of this work that gives additional diagnostic characters to genus level.

### Priasthenopus gilliesi

Taxon classificationAnimaliaEphemeropteraPolymitarcyidae

(Domínguez) comb. n.

Asthenopus gilliesi Domínguez 1988: 21; Hubbard and Domínguez 1988: 207; Domínguez et al. 2006: 562

#### Material.

Paratype male (IFML TEPH095, slide 041) from URUGUAY, Artigas, San Gregorio, orillas río Uruguay, 29.xi.1959, light trap, C.S. Carbonell col.; 5 male imagos (slide IBN141CM) and 1 male and 1 female (slide IBN471CM) subimagos (IBN) from COLOMBIA, Amazonas, P. N. Amacayacu, río Amacayacu, 93 m, S 3°48'28" − W 70°15'21", 3.ii.1999, light trap 18−20 h PM, E. Domínguez, M.C. Zúñiga & C. Molineri cols.; 5 male imagos and 1 female subimago (IBN) from BOLIVIA, Santa Cruz, near Once Por Ciento, río Blanco, 250 m, S 15°21'39.7" − W 63°17'28.8", 14.vi.2000, light trap, E. Domínguez col.; 1 female adult (CZNC) from BRAZIL, Rio N. Aripuanã, Rio Juma, Ig. Campineiro Gde., 8−9.ix.2004, Pennsylvãnia light trap; and 2 male imagos (CZNC) from Amazonas, Barcelos, rio Demene, ´boca´barco, 8−9.viii.2009, Pennsylvania light trap.

#### Diagnosis.

*Priasthenopus gilliesi* is known from adults of both sexes and eggs, and is the only species known in the genus. The characters useful to distinguish it from other Asthenopodinae are listed in the generic diagnosis.

Male imago. See generic section above and original description in Domínguez (1988).

Female subimago. Length (mm): body, 6.5; FW, 8.9; HW, 3.3; cerci, 1.5. General coloration yellowish white with gray markings. Head cream extensively shaded gray dorsally, the shading is uniform anteriorly but in the form of a fine netted pattern posteriorly to lateral ocelli, occiput with a pair of submedian pale anterior spots and a pair of submedian dark posterior spots; venter of head whitish. Antennae yellowish white shaded with gray on scape and pedicel. Thorax cream. Anterior ring of prothorax very thin, less than 1/4 the dorsal length of posterior ring; ratio width/length: 2.6; pronotum shaded blackish on median area except pale medial line, presternum paler, shaded gray before coxa. Mesonotum shaded very diffusely with gray, darker on longitudinal carinae and between posteroscutal protuberances; mesosternum and pleurae paler, shaded gray on anterior corner of katepisternum. Metanotum shaded gray on posterior half, except on a pale median triangular mark, shaded darker posteriorly to this pale mark; metasternum whitish. Legs whitish except coxae yellowish shaded gray and apex of hind trochanter pointed and yellowish orange. Wings (Figs 8H–I). Membrane of fore and hind wings slightly shaded with light brown, veins translucent shaded with light brown, markedly on larger veins (C, Sc, Rs); marginal intercalaries relatively long (Fig. 8H), MP_1_ fused with MP_2_ at base; 2 crossveins present between M and R stems basally to Rs fork. Abdomen whitish, shaded with gray dorsally and darkening posteriorly, shading on terga interrupted on medial paler line, this line is wide on terga 1–4, narrows posteriorly on 5–7, thin on 8, and widens posteriorly on 9–10; sterna whitish translucent except gill sclerites yellowish white; sternum 8 with anteromedian pair of relatively large sockets (Fig. 8J). Cerci whitish, about 0.2 the length of FW.

Eggs. See generic description.

#### Distribution.

This species presents a wide geographic range that spans from the Amazon River in the North to the Uruguay River in the South, also extending towards the West in Bolivian Chiquitania.

#### Discussion.

*Priasthenopus gilliesi* male imagos were adequately described by Domínguez (1988), females and eggs are described here for the first time. There are no morphological differences between the male imagos examined from the different localities, except for the penes of the Colombian males are slightly stouter, and those from the Bolivian males are slightly slender than the penes of the Uruguayan types. In the forewings, vein MP_1_ is basally free except on Bolivian males where this vein tends to fuse with MP_2_, although not completely.

### Campsurus paraquarius

Taxon classificationAnimaliaEphemeropteraPolymitarcyidae

Navás nom. n.

Campsurus paraguarius [lapsus] Navás 1920: 53; Lestage 1923: 122; Traver 1947b: 371; Hubbard 1982a: 271; Kluge 2004: 267.
Campsurus paraquarius ; Navás 1924a: 359.

#### Material examined.

None.

#### Discussion.

In the forewings of *Priasthenopus gilliesi*, vein MP_1_ is basally free except on Bolivian males where this vein tends to fuse with MP_2_, although not completely. This last arrangement of the MP sector is also present in *Campsurus paraquarius* Navas (1920) and, also coincident, are the length and arrangement of the two imv (intercalary marginal veinlets) figured by Navás, the relatively short ICu veins and the vein AA basally curved to CuP; also the small size of the male described by Navás coincide with *Priasthenopus* size range. Navás described the color of legs without saying that middle and hind legs are reduced (as *Campsurus*), so probably they were present and complete as in all Asthenopodinae. Finally Navás stated that the pronotum is wider than long (transverse), feature also present in *Priasthenopus* and related genera (*Asthenopus*, *Povilla*) but not in *Campsurus* males. Other species of Neotropical Asthenopodinae show shorter intercalary marginal veins (*Asthenopus* s.s.) or longer ICu veins (*Asthenopodes*). Lestage (1923) noted the similiraty of Navás species with the genus *Asthenopus* and Kluge (2004) treated this species in *Asthenopus* because of the arrangement of veins in the Cu sector of FW. We here coincide with these authors and because of the bad original description by Navás, and the apparent loss of type material, we treat the name *paraquarius* (Navás 1920) as a NOMEN NUDUM.

### Asthenopodes stat. n.

Taxon classificationAnimaliaEphemeropteraPolymitarcyidae

**Figure 12.**
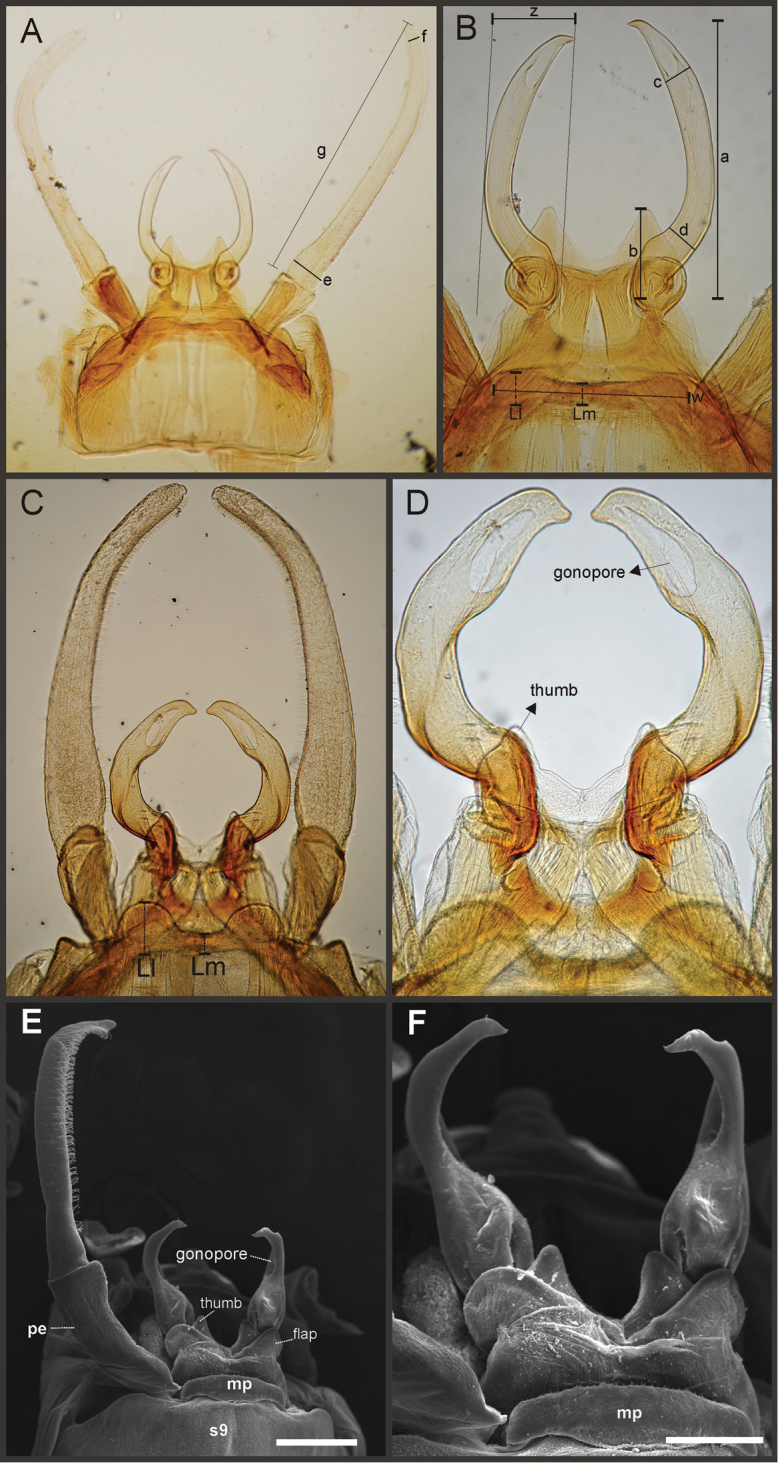
*Asthenopodes*, male genitalia: general view to the left, detail of penes to the right. **A–B** *Asthenopus picteti* **C–D** *Asthenopodes traverae* **E–F** *Asthenopodes chumuco*. Scale bar = 200 µ in **E** 100 µ in **F.** Abbreviations: mp = median plate of stryliger; pe = pedestal; s9 = ninth sternum; see Appendix 2 for explanation of measures (letters a to g, and z).

**Figure 13.**
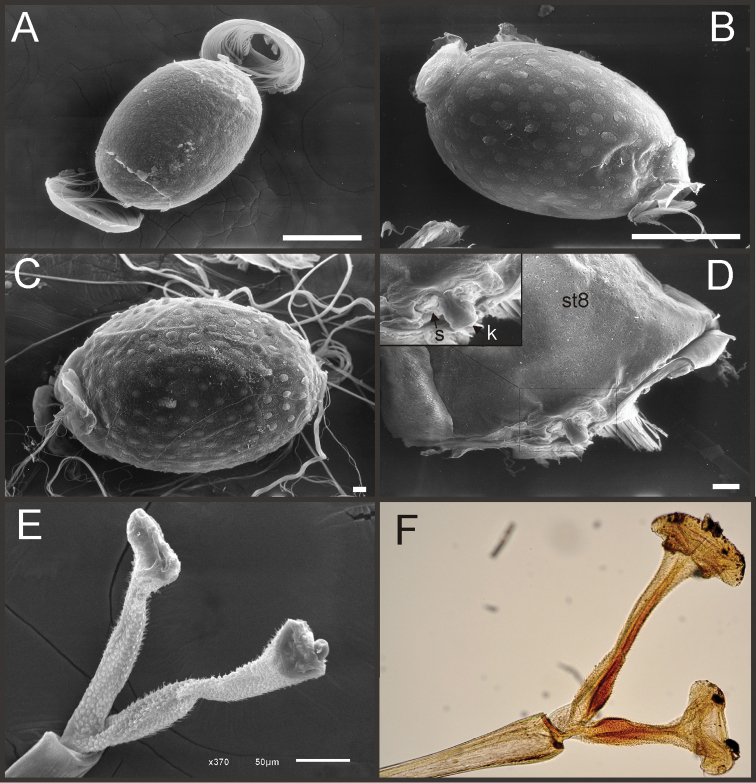
*Asthenopodes*, egg and adults. Eggs: **A** *Asthenopus picteti* **B** *Asthenopodes traverae* **C** *Asthenopodes chumuco*. Female imago: **D** *Asthenopodes traverae*, abdominal sternum VIII, and detail of keel (k) and sockets (s). Male imago foretarsal claw: **E** *Asthenopodes chumuco* **F** *Asthenopodes traverae*.

Figs 4B , 4F , 9 , 10 , 11 , 12 , 13 , 20A–C

Asthenopodes Ulmer 1924: 26; Traver 1950: 611; Traver 1956: 1; Hubbard 1975: 111; Hubbard and Domínguez 1988: 209; Domínguez 1988: 24.

#### Type species.

*Palingenia albicans* Pictet, original designation (= *Asthenopodes picteti* Hubbard)

#### Species included.

*Asthenopodes picteti* Hubbard, *Asthenopodes traverae* sp. n., *Asthenopodes chumuco* sp. n.

#### Diagnosis.

Seven autapomorphies define the genus *Asthenopodes* in our cladistic analysis (Appendix 2), some of them include: 1) Male foretarsal segment 1 fused with tibia (Figs 20A–C); 2) apex of male foretarsal claw strongly expanded (apex 3 times wider than stalk, Fig. 13E–F); and 3) pedestal relatively large, elongated, narrow at the base (Fig. 12). The following combination of characters representing the entire range of variation of the three included species, is useful to distinguish *Asthenopodes* from other genera in Polymitarcyidae: 1) ratio length male FW/foreleg = 1.0–1.6; 2) tarsal segment 1 indistinct (fused to tibia), tibia distally subequal in width to base of tarsal segment 2 (Figs 20A–C); 3) pronotum width/length ratio: 1.2–1.9 (male), 1.5–2.3 (female); 4) in both sexes FW marginal intercalary veins relatively long and anastomosed (from 9 to 22 in male FW, Fig. 11); 5) in both sexes FW with 3–6 (most commonly 4, but variable depending in size of specimen) crossveins between R and M, basally to R fork; 6) FW vein IMP slightly longer than MP_1_, both frequently free at base but may be joined to one another and to MP_2_ by crossveins (Fig. 11); 7) median remnant of styliger plate present in *Asthenopodes chumuco* (subrectangular as other Asthenopodinae) but medially very short and with a strongly marked lateral lobe in the sister pair *Asthenopus picteti*-*Asthenopodes traverae*; pedestals relatively large and thinner at the base; 8) forceps relatively slender, ratio length/basal-width = 4.7–9.5; 9) penes variable in form but curved medially rather than ventrally, basal thumb well separated from penial lobe, penial lobe apically acute (Fig. 12); 10) female abdominal sternum VIII with anteromedian paired sockets reduced in size (Fig. 13D); 11) eggs (Fig. 13A–C) with relatively small polar caps subequal to much thinner than egg, formed by 5–16 threads, chorion covered with medium-sized circular plates, surrounded by many smaller plates; 12) nymphal head dorsally strongly convex on occiput, frons not projecting medially (Figs 9A–B); 13) nymphs with very short and robust tusks (Figs 9C–H), without large submedian inner tubercle, with 2 or 3-pointed apex (asymmetric); 14) nymphal foretarsal claw with 2 rows of marginal denticles, a long row of 15 denticle and one shorter row of 12 denticles (Fig. 10B); 15) apex of femur dorsally with ca. 30 strong and rounded spines (Fig. 10D).

Male imago. Length (mm): body, 7.3–13.5; FW, 7.0–14.5; HW, 3.7–7.3; foreleg, 5.0–14; cerci, 20.0–38.5. Antennae: scape subequal to pedicel; flagellum bristle-like. Thorax. Pronotum width/length: 1.2–1.9. Legs. Forelegs relatively long, ratio length FW/foreleg = 1.0–1.6; tarsal segment 1 fused to tibiae (Fig. 20A–C); longest segment is tarsal segment 2 or tibia (variable), ratio length tarsal segment 2/tibia = 0.6–1.5; tarsal segments long decreasing in length from 2>3>4>5; claws different in size, one long the other short, strongly widened distally (Fig. 13E–F). Wings (Fig. 11). FW with 9–22 marginal intercalaries along hind margin, also present along entire hind margin of HW; these intercalaries present numerous connections with other cross and longitudinal veins (i.e., very anastomosed); 3–6 crossveins between R and M sectors basally to R fork; Rs stem length/Rs from fork to margin = 0.2–0.4; ratio MA from fork to margin/stem length = 9–15; IMP fused basally to MP_1_ or free; MP_2_ fused to IMP. Genitalia (Fig. 12): median remnant of styliger plate with posterolateral corners roundly projecting, pedestal long to very long; forceps relatively long and slender, ratio length/basal-width = 4.7–6.7. Terminal filament reduced, cerci long (ratio length FW/cercus = 0.32–0.44).

Female length (mm): body, 7.2–19.0; FW, 12.2–22.5; HW, 5.3–11.5; cerci 1.2–4.0. Thorax. Pronotum width/length = 1.5–2.3. Wings with crossveins and intercalaries more numerous than in male. Abdominal sternum VIII (Fig. 13D) with reduced paired anteromedian sockets, sockets small, shallow and contiguous located at the base of a blunt subquadrate projection. Terminal filament reduced, shorter than tergum VIII, with few thin annuli; cercus very short, 0.1–0.2 times the length of FW.

Eggs (Fig. 13A–C). Length, 221–355 μ; width, 143–240 μ. Oval (ratio maximum length / maximum width = 1.3–1.7), with two relatively small polar caps on apices (ratio maximum with of egg/maximum width of uncoiled polar cap = 1.2–3.1), each formed by 5–16 long coiled filaments. Chorionic surface with fine granulated aspect or regularly spaced plates.

Nymphs, nearly mature (Fig. 4B, F). Length (mm): body, 7.8; cerci, 2.0–2.3; terminal filament, 3.1. Head subquadrate in dorsal view, smooth (without pilose areas), antennae subequal in length to head. Occipital region well developed, convex (Figs 9A–B). Head capsule dorsally projecting at bases of antennae. Frontal ridge marked only by a dense transversal row of setae; frons not projecting medially; clypeus and labrum membranous and small, labrum densely covered with long setae on dorsum. Mandibular tusks very short and robust, the part visible in dorsal view ca. 1/3 the length of head capsule; left tusk (Fig. 9E–H) apically with 3 teeth, the median reduced in length, and the innermost is strongly widened appearing as a ridge with two points (Fig. 9F); right tusk (Fig. 9C–D, G–H) with 2 distal teeth; inner surface with a small tubercles located distally (in relation to other Asthenopodinae), dorsal surface with a small tubercle (“b” in Fig. 9G) that forms an additional articulation between mandible and head capsule; tusk densely covered with long setae, except at apex. Body of mandible: molae strongly protruded medially, canines present but small, margin between them sharp-edged (acutely protruding in right mandible); with basal U-row of long filtering setae in both mandibles. Thorax. Pronotum with anterior ring (collar) subequal in length to posterior ring (length taken at the medio-longitudinal line), anterolateral corners projecting, spine-like, posterior ring with dense patches of short setae medially (Figs 4F, 9B). Legs (Fig. 10A–D). Leg I (Fig. 10A): femora robust, relatively slender, with a U-shaped ventro-basal row of long filtering setae, distal points of the U almost touching each other; tibio-tarsus (fused) with 2 U-rows of filtering setae: 1 on anterior face (each branch well separated in the base) and 1 on inner margin, with the branches near each other, apex of tibio-tarsus relatively pointed; tarsal claw with 2 rows of marginal denticles (Fig. 10B). Leg II (Fig. 10C): smaller, with thinner femora, with scattered long setae basally and a row of long and short setae along outer margin; tibia and tarsi with row of long setae on outer (dorsal) margin, ventrally with many stout spines on apical half, anterior face of tibia with a distal row of thick setae (at base of tarsus) and with a crown of thick setae at apex; tarsal claw weaker, without denticles. Leg III (Fig. 10D): intermediate in size, outer margin of femur with row of short setae, longer at apex, distal corner of femur densely covered with thick, blunt setae; inner margin of femur, tibia and tarsus densely covered with short setae; margin between tibia and tarsus with row of thick setae; outer margin of tibia and tarsus with row of long pectinated setae. Coxae I and II directed ventrally, coxa III directed postero-laterally. Abdomen. Sternite I longer than the others and partially fused with metasternum. Gill I reduced in size, whitish, single and lanceolate. Gills II–VII well developed, ventral portion smaller than dorsal portion. Tergum X without posterolateral spine (Fig. 10E). Cerci slightly shorter than terminal filament, with long setae at joinings, basal 1/4 with thick blunt setae ventraly (Fig. 10F).

#### Distribution.

Amazonas and Parana subregions (Argentina, Brazil, Colombia, Guyana, Uruguay).

#### Discussion.

*Asthenopodes* and *Asthenopus* have been treated as synonyms (Hubbard and Domínguez 1988) after the discovery of *Priasthenopus gilliesi* (Domínguez, 1988), that somewhat blurred the distinction between both genera. Additionally, Hubbard and Domínguez (1988) based their synonymic proposal in the fact that all known nymphs of Asthenopodinae from South America where indistinguishable and could be classified in a single genus. As the knowledge of this group had largely improved in last years we are proposing here a new rearrengement of supraspecific taxa.

The characteristics traditionally associated with *Asthenopodes* (summarized in Domínguez 1988) are: 1) ratio foreleg/forewing of male: 7/8; 2) male foretarsus 3.5 times longer than foretibia; 3) foretarsal segment 2 very long (almost as long as tarsal segments 3 and 4 combined, and 1.5 times the length of tibia); 4) Rs fork base to margin: 2.5/10; 5) cubital intercalaries parallel, ICu_2_ arising basally from ICu_1_, or basally free but connected to ICu_1_ and CuP by cross veins, 6) ICu_2_ ending at hind margin, 7) long marginal intercalary veins present; 8) forceps ratio width/length: 1/10; 9) penes thin from the base; 10) MA fork base to margin 7/100; 11) IMP–MP_1_ fused; 12) MP_2_-IMP similar in length, fused; 13) foretarsal claws of male greatly expanded apically. The discovery of *Priasthenopus gilliesi* (Domínguez) put in doubt the value of characters 5, 7, 8, 9 and 10, for generic diagnoses, because this species showed some intermediary states between *Asthenopodes* and *Asthenopus* (Domínguez 1988, Hubbard and Domínguez 1988). In our phylogenetic analysis the intermediate position of *Priasthenopus gilliesi* is mantained, but it is clearly located outside both genera.

The revalidation of the genus *Asthenopodes* Ulmer is based not only in the clade that its type species (*Asthenopodes picteti*) forms with other two new species, but also on the fact that the nymph shows characters considered important at the generic level in the family, mainly the shape of nymphal mandibular tusks, legs and gill I.

#### Key to the species of *Asthenopodes*

**Male imagos**

**Table:** 

1	Large species (FW > 14 mm); wings smoky yellowish (Fig. 11E–F); penes markedly twisted and curved inwards (Fig. 12C–D).	***Asthenopodes traverae* sp. n.**
–	Smaller species (FW < 12 mm); wings hyaline, yellowish areas sometimes present along hind margin; penes much less curved and not twisted (Figs 12A–B, E–F)	**2**
2	FW 11.4–11.9 mm; genitalia as in Fig. 12A–B	***Asthenopus picteti* Hubbard**
–	Smaller species, FW 7.0–8.8 mm; genitalia as in Fig. 12E–F	***Asthenopodes chumuco* sp. n.**

**Female and eggs**

**Table:** 

1	FW with single imv in most spaces (Figs 11G,K); body blackish brown; eggs with small polar caps, ratio maximum width of egg/maximum width of PC = 2.3–3.1 (Figs 13B–C)	**2**
–	FW with double or triple imv (Fig. 11C); body yellowish light brown; polar caps wider, ratio maximum width of egg/maximum width of PC = 1.2–1.5 (Fig. 13A)	***Asthenopodes picteti***
2	Wings (Fig. 11G–H) translucent yellowish (transmitted light), large size (FW 15.5–22.5 mm); polar cap of eggs formed by 5–6 long coiled threads (Fig. 13B)	***Asthenopodes traverae***
–	Wings hyaline (Fig. 11K–L), smaller size (FW 12.2–14.8 mm); polar cap of eggs formed by 14–16 long coiled threads (Fig. 13C)	***Asthenopodes chumuco***

### Asthenopodes picteti

Taxon classificationAnimaliaEphemeropteraPolymitarcyidae

Hubbard stat. n.

Figs 11A–D , 12A–B , 13A , 20A

Palingenia albicans Pictet 1843: 149 (misidentification)
Campsurus albicans , Eaton 1871: 58.
Asthenopus albicans , Ulmer 1920c: 107, Ulmer 1921: 239, Lestage 1924c: 39.
Asthenopodes albicans , Ulmer 1924: 26, Traver 1956: 2.
Asthenopodes picteti , Hubbard 1975: 111, Domínguez 1988a: 24.
Asthenopus picteti , Hubbard and Domínguez 1988: 207, Domínguez et al. 2006: 562.

#### Type material.

Type material was not studied; it consists of the holotype male imago, damaged, with many parts missing including the genitalia. It is a pinned specimen deposited at Naturistorisches Museum Wien, Hubbard and Domínguez (1988) presented a figure of the holotype forewing.

**Additional material.** 3 male subimagos from ARGENTINA, Misiones, Parque Provincial Urugua-í, Arroyo Yacutinga, S 25°44'51" − W 54°03'37", 355 m, 30.xi.2001, Domínguez et al. cols.; 8 female and 2 male imagos same data except Arroyo Uruzú, S 25°51'29" − W 54°10'10", 322 m, 25.xi−2.xii.2001; 1 female and 1 male imagos (slides IBN3–93 and 3–96) from URUGUAY, Maldonado, Arroyo de la Quinta, 4.i.1984, M. T. Gillies col. All the material deposited in IBN.

#### Diagnosis.

*Asthenopodes picteti*, type species of the genus *Asthenopodes* (Ulmer 1924, Traver 1956, Hubbard 1975) presents 7 autapomorphies (Appendix 2) including: thinner male foretibia and slender penes. This species can be distinguished from the other species of the genus by the following combination of characters: 1) general coloration yellowish white (male), darker in female (yellowish light brown); 2) male FW 11.4–11.9 mm, female FW 14.5–19.0 mm, membrane whitish hyaline tinged with yellowish near hind margin; 3) foreleg length 0.9 times the length of FW; 4) pronotum width/length ratio: 1.6–1.9 (male), 1.5–2.3 (female); 5) 14–17 marginal intercalary veins present on the entire margin of FW (Fig. 11A) and HW (Fig. 11B), miv generally longer than distance between longitudinal veins; 6) male FW with 4 to 6 cross veins between Rs and MA basal to Rs fork; 7) ratio total length/basal width of forceps 6.7 (Fig. 12A); 8) penes long and slender (Fig. 12A–B), male median remnant of styliger plate posterolaterally expanded forming a pair of rounded projections (“Ll” in Fig. 12B); 9) female sternum VIII, anteromedian sockets present but concolorous with sternum, not evident; 10) egg polar caps only slightly thinner than maximum width of the egg, formed by 6 filaments; medium-sized and small chorionic disks-like structures present (Fig. 13A).

Male imago. Length (mm): body, 9.3–11.2; FW, 11.4–11.9; HW, 5.8–6.2; foreleg, 10.0–10.3; cerci, 30.1. Described in Traver (1956) and Domínguez (1988a). Additions to these descriptions follow: Prothorax width/length: 1.6–1.9. Wings. Hind margin of FW (Fig. 11A) with 14–17 marginal intercalary veins, relatively long; 4–6 cross veins between R and M, basal to R stem; IMP basally free or fused to MP_1_ by a cross veins, MP_2_ fused to IMP. HW as in Fig. 11B. Genitalia (Fig. 12A–B): forceps length/width ratio: 6.7; median remnant of styliger plate with posterolateral corners roundly projecting.

Female adult. Length (mm): body, 11.5–12.6; FW, 14.5–19.0; HW, 6.3–9.4. General coloration yellowish light brown. Head dorsally blackish except on median zone, paler; venter of head yellowish white. Antennae light brown, shaded gray on scape. Thorax yellowish brown with blackish membranes, shaded with brownish gray on pronotum and with black on posteromedian marks on meso- and metanotum. Pronotum width/length: 1.5–2.3. Legs whitish yellow shaded brownish on dorsum of leg I and on apex of femur III. Wings (Fig. 11C–D), membrane tinged with light brown, veins yellowish brown. Abdomen. Terga brownish with a pale mediolongitudinal line and paler areas on lateral margins on terga I–VII; sterna whitish yellow; female sternum VIII with anteromedian sockets, small and almost not distinguishable. Terminal filament whitish, shorter than tergum VIII; cerci yellowish brown paler apically, 0.5–0.6 times the length of the abdomen (0.2 the length of FW).

Eggs (Fig. 13A). Length, 325−355 µ; width, 215−235 µ. Two large and flat polar caps (maximum width, 155−180 µ), formed by 6 very long coiled threads. Chorionic surface with a fine granulated aspect, with small disk like structures.

#### Distribution.

Uruguay, Argentina. *Asthenopus picteti* is here newly recorded from Argentina. The record from Guyana given by Domínguez et al. (2006, p.562) is no longer valid since this material is now considered a different species (*Asthenopodes chumuco* see below).

#### Discussion.

*Asthenopodes picteti* Hubbard was only partially known from the damaged holotype male from Brazil until Traver (1956) redescribed it (at that time as *Asthenopodes albicans*) based on a complete male from Uruguay. Later Hubbard (1975) gave a new name to this species (*Asthenopus picteti*). Domínguez (1988) and Hubbard and Domínguez (1988) presented additional discussion and illustrations of the male imago, from type and non-type material (also collected in Uruguay). The description of Traver (1956) coincides with the new material studied, except for the relation of the male foretibia and forefemur. Traver reported that tibia is 1.33 times the length of femur but we found that it is 1.7 times that length.

Domínguez (1988) reported 2 female imagos collected at the same time than the males he redescribed, but did not presented a formal description of them. Both females and additional ones from Argentina (see list of material) are here shortly described and figured. It is difficult from the material available to determine if the females are in subimaginal or imaginal stage, so they are here referred as “adults”. The females are similar to the sympatric *Asthenopodes traverae* but can be separated from this species because *Asthenopus picteti* females are lighter in color, hind femora only shaded black on apex, paired female sockets present but small and hard to distinguish, cerci light colored, abdominal gill sclerites smaller, FW wider with shorter marginal intercalaries joined to main veins and partially anastomosed. The eggs present wider polar caps, only slightly thinner than the egg. Males can be separated from those of *Asthenopodes traverae* because the hind femora are only shaded black on apex and the penes are much slender.

### Asthenopodes traverae sp. n.

Taxon classificationAnimaliaEphemeropteraPolymitarcyidae

http://zoobank.org/D3A80802-232F-4930-99FC-D7C0EDC862D1

Figs 11E–H , 12C–D , 13B, D, F , 20C

Asthenopodes sp. “females from Uruguay”, Traver 1956: 5.

#### Material.

Holotype (IBN) male and paratypes (IBN) 4 males and 22 females (slides IBN468–469CM) from ARGENTINA, Misiones, Parque Provincial Urugua-í, Arroyo Uruzú, 7−11.xii.1999, C. Molineri col.; 1 female imago (“*Asthenopodes* sp.” Traver det.) from URUGUAY, Artigas (Uruguay 19), 9.i.1952; and 27 female adults from URUGUAY, Artigas, Sepulturas (D-3), 18.xii.1952, Carbonell col. Paratypes from Uruguay deposited in FCE-Ep.

Non-type material: 1 female (wings on slide) from BRAZIL, Sao Paulo, Jacareí, rio Paraiba do Sul, 21.xi.1987, CG Froehlich et al. cols (deposited at MZSP).

#### Diagnosis.

Four autapomorphies characterize this species, all are small changes in continuous characters, except the marked shortening of the median remnant of styliger plate (at the middle, since laterally a tong-like projection is present, Fig. 12C). *Asthenopodes traverae* sp. n. is known in the alate and egg stages, all females are subimagos even those that apparently have laid the eggs (empty females). This species can be distinguished from the other species of the genus by the following combination of characters: 1) males yellowish white, females blackish; 2) FW length male 14.0–14.5 mm, female 15.5–22.5 mm, membrane yellowish hyaline; 3) male foreleg length 0.9–1.0 times the length of FW; 4) pronotum width/length ratio: 1.3–1.9 (male), 2.0 (female); 5) 16–22 marginal intercalary veins present on the entire margin of FW (also present in HW) generally longer than distance between longitudinal veins, fused to main veins, poorly anastomosed (Fig. 11E–H); 6) male FW with 4 to 5 cross veins between Rs and MA basal to Rs fork; 7) ratio total length/basal width of forceps 4.7–4.9 (Fig. 12C–D); 8) median remnant of styliger plate posterolaterally expanded forming a pair of rounded projections, penes large and robust, sclerotized, curved and twisted (Fig. 12C–D); 9) female sternum VIII with anteromedian sockets well developed, whitish surrounded by a large brownish area (Fig. 13D); 10) egg caps small, much thinner than maximum width of the egg, formed by 5–6 filaments; medium-sized and small chorionic plates present (Fig. 13B).

Male imago. Length (mm): body, 12.2–13.5; FW, 14.0–14.5; HW, 6.8–7.3; foreleg, 12.3–14.0; cerci, 35.5–38.5. General coloration yellowish white. Head shaded black dorsally almost entirely, with a pair of distinct submedian black marks anteriorly to median ocellus; occipital hind margin with pale median zone; head ventrally pale without markings. Antennae: scape and pedicel short, yellowish on venter of pedicel, both slightly shaded with gray; flagellum very thin, hyaline, similar in length to forefemur. Thorax. Pronotum translucent, shaded slightly with gray except on membranes separating anterior and posterior rings, darker laterally; pronotum width/length: 1.3–1.9. Meso- and metanotum yellowish white with gray markings mainly posteriorly but also on sutures. Thoracic pleurae and sterna yellowish white shaded gray only at base of coxae and wings. Legs. Forelegs: coxa dorsally whitish with a gray mark, ventrally yellowish; femur dorsally yellowish shaded gray on apical third, ventrally whitish; tibia whitish translucent shaded gray mainly on dorsum; tarsi translucent shaded slightly with gray; large claws, stalk with brown inner margin, rest whitish; articulations between femur-tibia and tibia-tarsus very sclerotized, brownish. Middle and hind legs yellowish, shaded with gray from half of femur to apex of leg. Wings (Fig. 11E–H). Membrane hyaline slightly tinged with gray on costal and subcostal sectors (membrane yellowish under transmitted light); all veins light gray, lighter toward hind margin; 5 cross veins between R stem and M sector; long marginal intercalaries on hind margin of both wings. Abdomen yellowish white widely shaded with gray, some darker marks as follows: submedian short anterior dashes and sublateral oblique stripes on terga III–VIII, lateral margins of terga VIII–IX, and median line of IX–X (thinner on X). Abdominal sterna whitish except gill sclerites yellowish white. Genitalia (Fig. 12C–D): lateral margins of sternum IX and pedestals yellowish; median remnant of styliger plate whitish with a gray strip on hind margin between the tongue-like projections (“Ll”Fig. in Fig. 12D); pedestals well separated from each other, relatively long and becoming wider distally; forceps whitish, long and wide; base of penes well developed, subquadrate, whitish; penes strongly sclerotized, orangeish basally but yellowish distally. Cerci whitish, terminal filament reduced to 7–8 thin annuli, straight, whitish.

Female subimago. Length (mm): body, 9.5–19.0; forewing, 15.5–22.5; hind wing, 7.5–11.5; cerci 3.5–4.0. General coloration dark brown shaded widely with black. Head black dorsally, yellowish white ventrally except on remnants of tusks, brownish. Antennae dark brownish except apical half of flagellum whitish. Thorax. Sclerites dark brown, membranes shaded black. Pronotum width/length: 2. Legs brownish except membranous portions, whitish. Wings (Fig. 11G–H) with yellowish brown membranes and veins, veins C and Sc darker. Abdomen. Terga dark brown completely shaded black; sterna paler, brownish laterally, yellowish medially, with two or more pairs of small pale dots; sternum VIII with two small anteromedian sockets (Fig. 13D). Caudal filaments brownish, terminal filament shorter than tergum VIII, cerci 0.3–0.4 times the length of the abdomen (0.2 the length of FW).

Eggs (Fig. 13B). Length, 305–355 µ; width, 195–225 µ. Two small polar caps (maximum width, 70–82.5 µ) formed by 5–6 long coiled threads. Chorionic surface smooth with regularly spaced chorionic plates. Chorionic plates (“disk-like” structures) relatively small and rounded, with irregular margins. Smaller plates, irregular in form scattered between the larger ones.

#### Etymology.

The species is dedicated to the great mayfly specialist Jay R Traver, who visited Uruguay and worked with Mr. C. S. Carbonell’s collections at the “Museo de la República” recognizing the females of this species as distinct from *Asthenopus picteti* (also unknown at that time).

#### Distribution.

Parana biogeographic subregion in Argentina, Uruguay and Brazil.

#### Biological remarks.

Females subimagos were collected (in Misiones Province) while swarming in compact groups at about 3 m above water in pool areas around sunset. The same behavior was reported by Traver (1956) for the Uruguayan females. Males were caught at light traps during the first hours of dark, so the male flight is unknown.

#### Discussion.

Traver (1956) described females of *Asthenopodes traverae* as distinct from *Asthenopus picteti* in spite of the fact that the female of that species was also unknown at that time. Nevertheless Traver realized that they differ from *Asthenopus picteti* males in color and size, and left them unnamed. With the collection of new material from both species (and sexes) in Misiones (Argentina), it became evident that they constitute a new species. *Asthenopodes traverae* females can be distinguished from *Asthenopus picteti* females by their black general coloration, somewhat slender forewings, femora and cerci widely shaded with black, and enlarged abdominal gill sclerites (remnants of nymphal gill muscles insertions), for other differences see discussion under *Asthenopus picteti*. Very similar in aspect but much smaller are the females of *Asthenopodes chumuco*, the allopatric sister species of *Asthenopodes traverae*.

### Asthenopodes chumuco sp. n.

Taxon classificationAnimaliaEphemeropteraPolymitarcyidae

http://zoobank.org/DEC97104-1A64-4492-B34A-719E784CE531

Figs 4B, F , 9 , 10 , 11I–L , 12E–F , 13C, E , 20B

Asthenopodes sp? Traver 1950: 611.
Asthenopus picteti Domínguez et al. 2006: 562.

#### Type material.

Holotype and 3 paratypes male imagos from Brazil, Amazonas, Barcelos, rio Demene, ´boca´barco, S 0°25'28.7" - W 62°54'20", 8−9.viii.2009, Pennsylvania. Holotype and 1 paratype in INPA, 2 paratypes in IBN.

**Additional material.** 3 male slides (CUIC) from British Guiana, Bartica District, Kartabo, 20.iv.1919, C.U. Expedition col. Five female imagos (1 in CZNC, 2 in IBN, IBN533CM, 2 in MUSENUV) from Colombia, Amazonas, Puerto Nariño, Loreto Yacu, S 3°44'26" − W 70°27'19", 5.ii.1999, luz 18−20 h, M. C. Zúñiga, E. Domínguez and C. Molineri cols.

#### Diagnosis.

*Asthenopodes chumuco* known from all the stages presents seven autapomorphies, all of them are changes in continuous characters (Appendix 2). This species can be distinguished from the other species of the genus by the following combination of characters: 1) general coloration yellowish white in male, dark brown in female; 2) male FW 7.0–9.0 mm, female FW 12.2–14.8 mm; 3) male, ratio FW/foreleg length 1.4–1.6; 4) pronotum width/length male 1.3–1.4, female 2.0–2.1; 5) 7–14 marginal intercalary veins present on the entire margin of FW (Fig. 11I, K), HW (Fig. 11J, L) with 3–7 marginal intercalary veins, generally longer than distance between longitudinal veins, fused to main veins, poorly to heavily anastomosed; 6) male FW (Fig. 11I) with 3–4 cross veins between Rs and MA basal to Rs fork; 7) ratio total length/basal width of forceps 7.8–9.5 (twisted CUIC slide 6.3–6.7) (Fig. 12E); 8) male median remnant of styliger plate subrectangular, slightly convex (not expanded forming a pair of rounded lateral projections), penes long and slender, acute distally (Fig. 12E–F), rectangular pedestals strongly enlarged, ½ the length of forceps; 9) female sternum VIII, with small reduced anteromedian female sockets at the base of a median keel; 10) egg caps small, much thinner than maximum width of the egg, formed by 14–16 filaments; large and small chorionic plates present (Fig. 13C).

Male imago. Length (mm): body, 7.3–8.0; forewing, 7.0–8.8; hind wing, 3.7–4.3; foreleg, 5.0–5.8; cerci, 20.0–23.0. General coloration yellowish white. Head shaded gray dorsally almost entirely, frons with black dot at base of antenna, with medial line and irregular black marks; occipital region with pale median zone; head ventrally pale without markings. Antennae: scape and pedicel short, subequal in length, whitish shaded with purplish; flagellum very thin, hyaline. Thorax. Pronotum whitish with anterior and posterior portion subequal in size, shaded gray in a transverse band between both portions, posterior portion black along hind and lateral margins; pronotum width/length: 1.2–1.3. Mesonotum yellowish white shaded with gray on posterior half of medial line, on area between posterolateral protuberances and on anterior margin of these structures. Metanotum yellowish slighlty shaded with gray medially. Thoracic pleurae and sterna yellowish white shaded gray dorsally and anteriorly to mid coxa. Legs. Forelegs: whitish completely shaded with gray; large claws, apically expanded (Fig. 13E). Middle and hind legs yellowish white, shaded with gray on coxae. Wings (Fig. 11I–J). Membrane hyaline shaded with gray on basal 1/3 of costal and subcostal sectors; all veins translucent, except costal cross veins grayish; 3–4 cross veins between R stem and M sector; long marginal intercalaries on hind margin of both wings. Abdomen whitish shaded with gray on terga, mainly on lateral margin, submedian black dot on terga III–VIII. Abdominal sterna pale. Genitalia (Fig. 12E–F): yellowish white, except for penis apically yellowish; pedestals well separated from each other, very long, ½ length of forceps; median remnant of styliger plate with slightly convex hind margin; base of penis rounded, projecting posterolaterally, lobe of penis long and sclerotized, curved ventromedially, constricted on median length, gonopore well developed. Cerci whitish; terminal filament reduced to 5–7 thin annuli, straight, whitish.

Note: Cornell male (slides, male imago). Length (mm): body missing; FW, 9.0; HW, 4.5. FW with 11 long marginal intercalary veins; 3 cross veins between R and M basad to R stem; IMP fused basally to MP_1_ or free; MP_2_ fused to IMP. HW with 6 long intercalary veins on hind margin. Genitalia: penes robust, twisted; median remnant of styliger plate without posterolateral projections; forceps long and slender, ratio length/basal width: 6.3–6.7.

Female imago. Length: body, 7.2 (shrunken, empty)–12.3; FW, 12.2–14.8; HW, 5.3–6.0; cerci, 1.2–1.3. General coloration dark brown. Head dorsally black except on clypeus, whitish with a pair of lateral brownish bands, ventraly much paler brownish white. Thorax brownish with blackish membranes and carinae. Pronotum with a pair of distinct black marks submedially; width/length ratio: 2. Legs pale, brownish white. Wings (Fig. 11K–L) with hyaline membrane, slightly whitish translucent; veins whitish tinged with brownish basally; 2 to 4 crossveins between M and R, basally to R stem; MP_2_ joined basally to IMP; marginal intercalaries relatively numerous and long, anastomosed. Abdomen. Terga brownish slightly paler on median band and pleural folds, sterna much paler brownish white. Cerci basally brownish turning whitish distally; very reduced in size, less than 0.1 the length of FW.

Eggs. Length 210–240 µ, width 180–200 µ. Subovate, yellowish, with two small whitish polar caps (maximum width, 75–85 µ), polar caps much thinner than the egg and formed by 14–16 threads. Under SEM the larger disk-like chorionic structures are surrounded by many smaller ones, which at their time are surrounded by smooth chorion (Fig. 13C).

Nymphs. Length (mm): body, 7.8; cerci, 2.0–2.3; terminal filament, 3.1. General coloration yellowish light gray (Fig. 4B, F). Head with a black band between lateral ocelli and fine netting pattern on occiput (Fig. 4F). Antennae: scape bare, slightly longer than pedicel, pedicel with many dorsal setae, flagellum bare with numerous annuli increasing in length distally. Thorax. Pronotum shaded black on sublateral area of anterior ring and laterally on posterior ring. Meso- and metanotum shaded widely with gray, with dark gray wingbuds, developing veins paler. Legs (Fig. 10A–D). Coxae and trochanters of mid and hind legs slightly shaded with gray, remainder of legs yellowish-white; foretarsal claw with double parallel rows of 15 and 12 denticles each (Fig. 10B). Abdomen. Terga more or less uniformly shaded brownish-gray, except on pale transverse dashes laterally, thin medial line on tergum I–IX becoming wider posteriorly and pale with subcircular submedian sigilla; sterna yellowish. Gill I whitish, gills II–VII purplish gray, ventral portion paler than dorsal portion. Caudal filaments yellowish.

#### Etymology.

“Chumuco” is one of the common names applied to river cormorans in some South American countries. The penis lobe of this new species resembles the neck and head of that bird.

#### Distribution.

Brazil (Amazonas, Espírito Santo), Colombia (Amazonas), Guyana.

#### Discussion.

The male imago of this species has been known since Traver (1950) found a group of slides containing some body parts of a missing specimen. She stated that it was surely not the type species of *Asthenopodes* mainly because its smaller size. Domínguez et al. 2006 (p. 562) treated this specimen in *Asthenopus picteti*, extending the distribution of this last species to British Guiana. With the discovery of new specimens (male and female adults) we gathered more morphological information and realized that a new species must be described. One of the results is that *Asthenopus picteti* is no longer considered to be in British Guiana. The Colombian females described here as *Asthenopodes chumuco* are associated with the males, because of the egg morphology, compared with those extracted from a pharate female from São Mateus. Adults show the smallest size of the genus, further differing from the other two species in many morphological aspects. The females are similar to *Asthenopus picteti* in coloration of hind femur and abdomen but wing venation and sockets on sternum VIII are different, also the female cerci of *Asthenopodes chumuco* are much less developed. The eggs are similar to those of *Asthenopodes traverae*, because of the small polar caps, nevertheless *Asthenopodes chumuco* presents much more threads forming each cap (between 14 and 16, but each thread is relatively very thin).

### Asthenopus

Taxon classificationAnimaliaEphemeropteraPolymitarcyidae

Eaton

**Figure 14.**
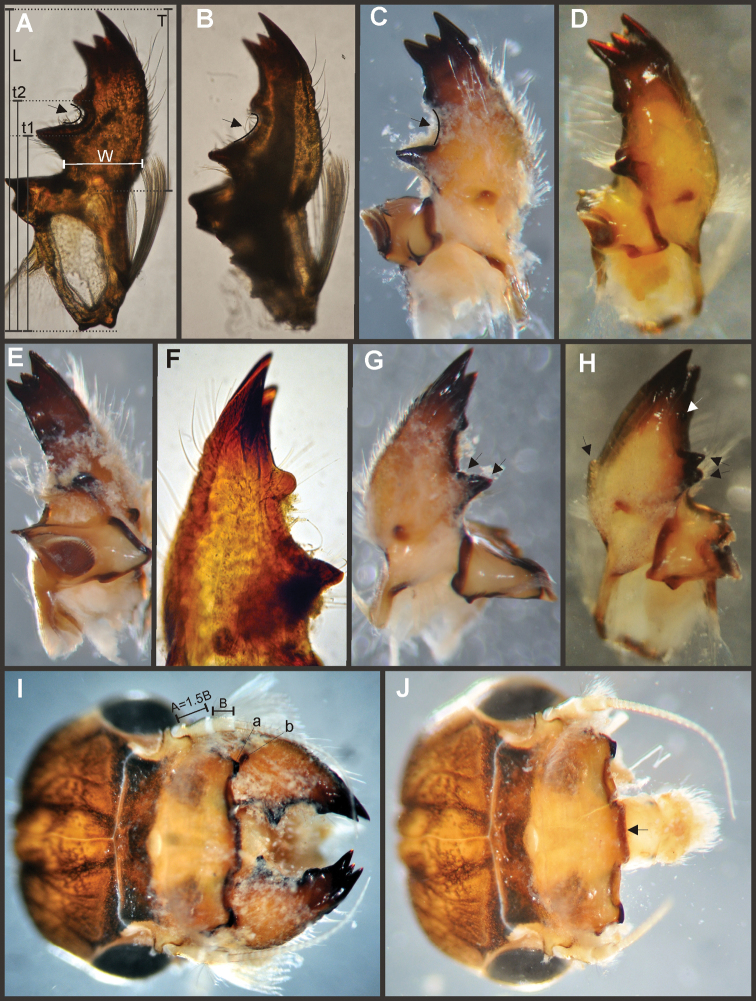
*Asthenopus* (and *Povilla*) nymphs, head and mandibles. **A–D** left mandibles, d.v.: **A** *Asthenopus curtus* **B** *Asthenopus angelae* **C** *Asthenopus magnus* **D** *Povilla adusta*. **E** left mandible dorso-oclusal view, *Asthenopus magnus*. Right mandibles, d.v.: **F** *Asthenopus angelae*, **G** *Asthenopus magnus* **H** *Povilla adusta*. **I–J**, *Asthenopus magnus* head capsule, d. v., with and without mandibles.

**Figure 15.**
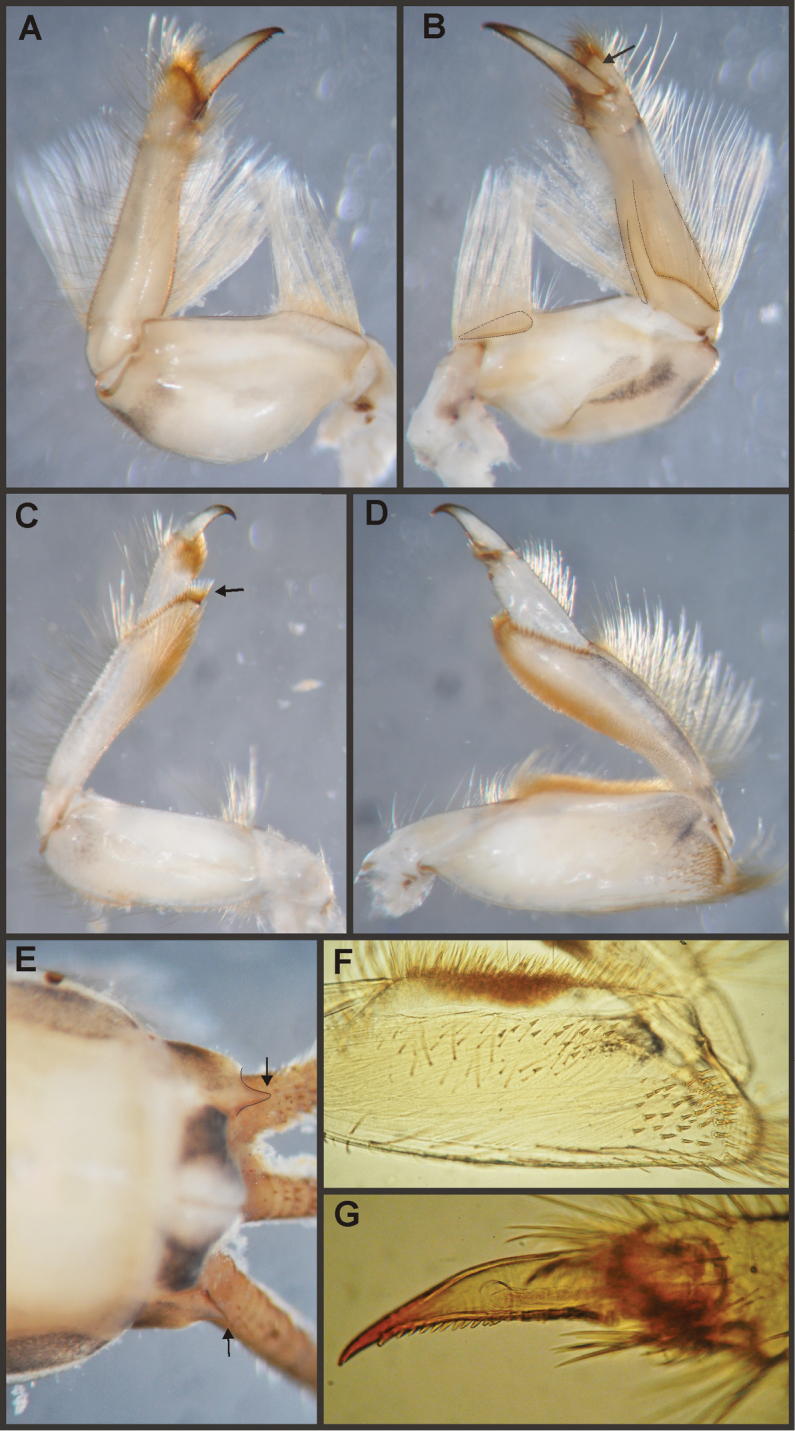
*Asthenopus* nymphs. *Asthenopus magnus*: **A** foreleg, d.v. **B** same, v.v. (arrow indicates apical projection of tibiotarsus) **C** middle leg, d.v. (arrow indicates distal brush on tibia) **D** hind leg **E** abdominal sterna **IX–X** (arrow indicates spine on paraproct). *Asthenopus angelae*: **F** hind femur, d.v. **G** foretarsal claw. **A–G** stereomicroscope photographs **F–G** light microscope photographs.

**Figure 16.**
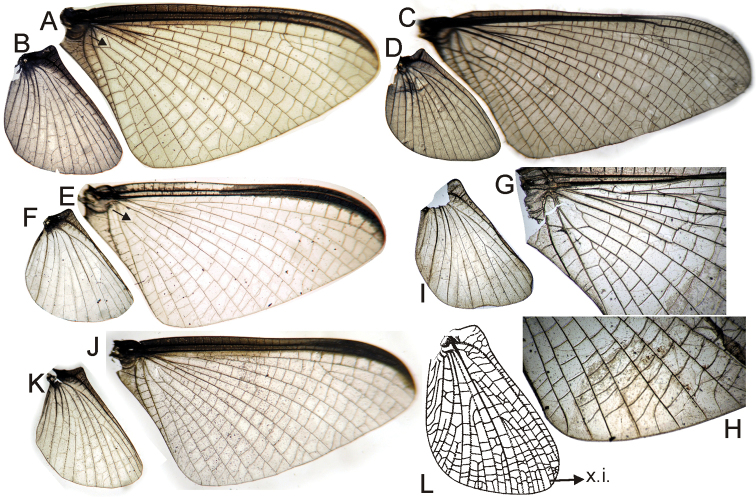
*Asthenopus* fore (FW) and hind wings (HW) of male imago. **A–B** *Asthenopus curtus* FW & HW **C–D** *Asthenopus magnus* FW & HW **E–F** *Asthenopus hubbardi* FW & HW **G–I** *Asthenopus guarani*, FW (details) & HW **J–K** *Asthenopus angelae* (from Argentina) FW & HW. *Ephoron* sp.: **L** male HW (x.i. = extra intercalary). Stereomicroscope photographs (except **L** line drawing).

**Figure 17.**
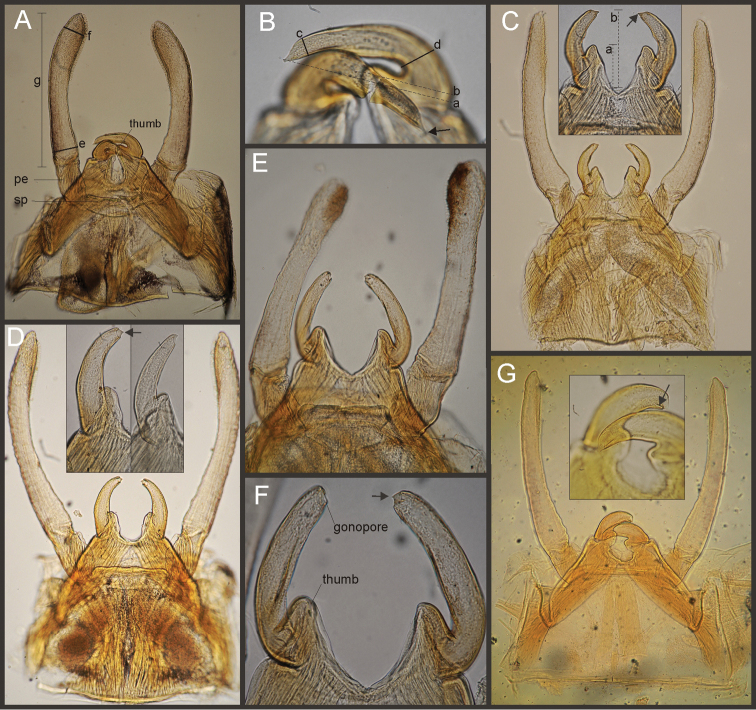
*Asthenopus*, general ventral view of male genitalia and detail of penes. **A–B** *Asthenopus curtus* **C** *Asthenopus hubbardi* **D** *Asthenopus angelae* **E–F** *Asthenopus guarani* **G** *Asthenopus magnus*. Light microscope photographs.

**Figure 18.**
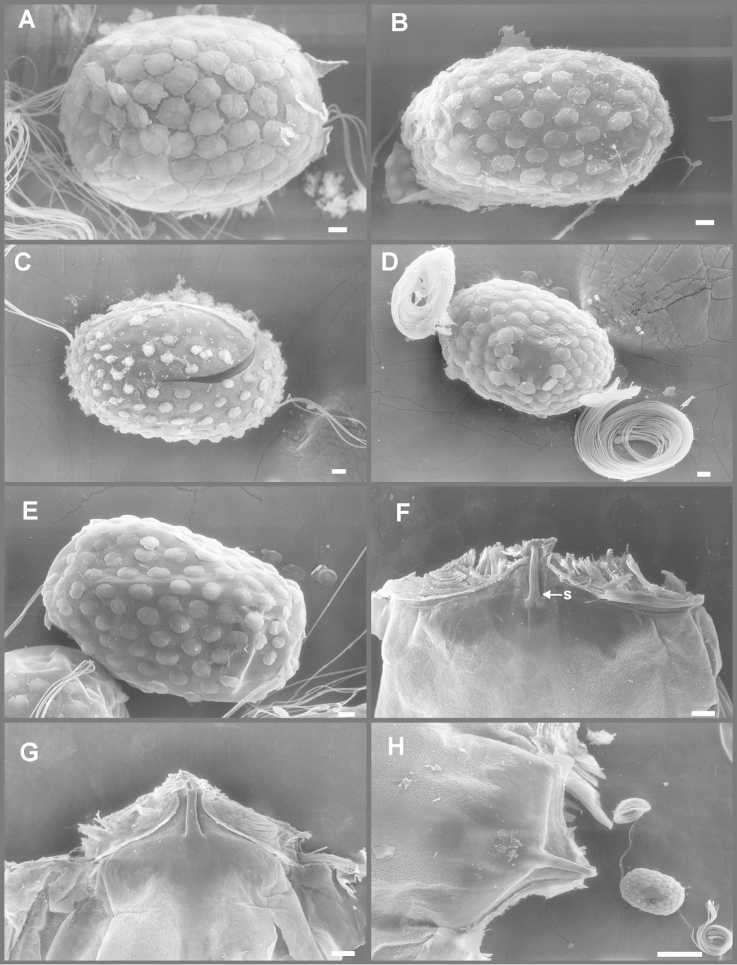
*Asthenopus*, SEM photographs. Eggs: **A** *Asthenopus curtus* (egg extracted from mature nymph), **B** *Asthenopus angelae* (egg extracted from mature nymph) **C** Asthenopus cf. guarani **D** *Asthenopus hubbardi*, **E** *Asthenopus magnus*. Female abdominal sternum VIII: **F** *Asthenopus magnus* **G** *Asthenopus curtus* **H** *Asthenopus hubbardi*.

Figs 4C–D , 14 , 15 , 16 , 17 , 18 , 20F–J

Asthenopus Eaton 1871: 59; Lestage 1922: 142; Traver 1950: 605; Traver 1956b: 7; Hubbard and Domínguez 1988: 209; Domínguez 1988a: 24.

#### Type-species.

*Palingenia curta* Hagen, original designation.

#### Species included.

*Asthenopus curtus*, *Asthenopus angelae*, *Asthenopus magnus* sp. n., *Asthenopus hubbardi* sp. n., *Asthenopus guarani* sp. n.

#### Diagnosis.

Five autapomorphies were recovered for *Asthenopus*: 1) character 1 (ratio length second foretarsal segment/foretibia) decreases from 0.645–0.652 to 0.584–0.587; 2) char. 7 (marginal connectivity = n°of connections among imv/n°of imv) decreases from 0.700–1.167 to 0.222–0.300; 3) male foretarsal segment 1 subrectangular; 4) penes with an apical spine; and 5) long and open cleft present between penial lobe and thumb. The following combination of characters is useful to distinguish *Asthenopus* from other genera in Polymitarcyidae: 1) ratio length male FW/foreleg = 1.4–1.8; 2) tarsal segment 1 distinct and subrectangular in form (not fused to tibia), ratio subapical width of foretibia/subbasal width of second tarsal segment 1.5–2.3 (Fig. 20F–J); 3) pronotum width/length ratio 1.7–2.4 (male, but frequently around 2.0), 2.0–3.0 (female); 4) in both sexes FW marginal intercalary veins short and not anastomosed (4–25 in number, Fig. 16); 5) in both sexes FW with 0–4 (most commonly 2, but variable depending on size of specimen) crossveins between R and M, basally to R fork; 6) FW vein IMP basally free, subequal in length to MP_2_, forming a characteristic oblique “Y” with associated cross veins (arrow Fig. 16A, E); 7) median remnant of styliger plate present, pedestals well developed (Fig. 17); 8) forceps relatively stout, ratio length/basal-width = 4.7–7.0; 9) penes relatively short and stout, curved ventrally (and medially), with a thumb attached at the base, and with the apex of penial lobe distinctly shaped as a spine; 10) female abdominal sternum VIII with anteromedian paired sockets, much reduced in size, and with a keel as in Figs 18F–H; 11) eggs with relatively large polar caps formed by 3–8 (commonly 5) long coiled threads, chorion covered with disk-like structures (Fig. 18A–E); 12) head dorsally strongly convex on occiput, frons not projecting medially (Fig. 14I–J); 13) nymphs with very short and robust tusks (Fig. 14A–C, E–G), with large submedian inner tubercle, with 2 or 3-pointed apex (asymmetric); 14) nymphal foretarsal claw with a single row of denticles (Fig. 15G); 15) apex of femur 3 with a group of ca. 50 stout pointed spines (Fig. 15F).

Male imago. Length: body, 6.5–10.5; FW, 7.0–10.1; HW, 2.9–4.8; foreleg, 5.1–7.5; cerci, 22.0–37.0. Antennae: scape slightly longer than pedicel, flagellum bristle-like. Thorax. Pronotum width/length: 1.7–2.4. Legs. Forelegs subequal to shorter than body, ratio length FW/foreleg 1.4–1.8; longest segment is tibia (ratio length tarsal segment 2/tibia = 0.4–0.7); tarsal segment 1 distinct, not fused, very short (Fig. 20F–J); remaining tarsal segments relatively short subequal in length except tarsal segment 2 slightly longer; claws differing in length, one long the other short, very slightly widened distally. Wings (Fig. 16). FW with 4–25 marginal intercalaries, sometimes present in HW but not in all spaces; intercalaries not strongly anastomosed; 0–4 cross veins between MA and R, basad to R stem; R stem length/Rs from fork to margin = 0.17–0.31. Ratio MA length from fork to margin/stem length = 7–15; IMP fused basally to MP_1_ or free; MP_2_ fused to IMP. Genitalia (Fig. 17): median remnant of styliger plate subrectangular to subovate; pedestals subrectangular to subovate, relatively small; forceps relatively wide to very wide, ratio length/basal width 4.7–7.0; penes tubular and robust, curved ventro-medially, and with well-developed basal thumb. Terminal filament reduced, cerci long (ratio length FW/cercus = 0.25–0.36).

Female adult. Length: body, 8.0–19.5; FW, 12.0–18.5; HW, 4.6–7.8; cerci, 3.0–7.0. Thorax. Pronotum width/length = 2–3. Wings with more crossveins and intercalaries than in male. Abdominal sternum VIII with anteromedian keel (Fig. 18F–H), at each side of keel´s base a very small “socket” is present (“s” in Fig. 18F). Terminal filament reduced, shorter than tergum VIII, with few thin annuli. Cercus short, 0.25–0.50 times length of FW.

Eggs (Fig. 18A–E). Length, 210–285 µ; width, 135–163 µ. Oval (ratio maximum length / maximum width = 1.4–1.8), with two relatively large polar caps (ratio maximum with of egg/maximum width of uncoiled polar cap = 1.1–1.5), formed by 3–8 very long coiled threads. Chorionic surface with large subcircular chorionic plates, sometimes each plate is divided in 2–3 portions.

Nymphs. Length (mm): body, 9.7–15.0 mm; cerci, 4.0–7.0; terminal filament, 5.0–5.1. Head suboval in dorsal view, smooth (without pilose area); occipital region well developed, strongly convex (Figs 4C–D, 14I–J). Head capsule with a dorsal spine-like projection at bases of antennae. Antennae 1.1–1.5 times length of head (length of head taken from hind margin to the apex of clypeus); pedicel with tuft of setae on dorsum, flagellum with minute scattered setae; length (mm): scape (0.5), pedicel (0.28), flagellum (2.0). Frons with anterior margin more or less straight (arrow in Fig. 14J), with a small blunt lateral projection (“a” in Fig. 14I), without median projection. Clypeus and labrum small, membranous, with many setae on dorsum of labrum. Mandibular tusks robust, relatively stout, left tusk (Fig. 14A–C, E) with 3 apical teeth, increasing in size from the median (smallest), inner and outer; inner tooth slightly directed medially, others directed distally; right tusk (Fig. 14F–G) with 2 teeth, the inner shorter. Inner margin of both tusks with a rounded small tubercle near subapex and a larger and pointed subbasal tubercle (associated with a tuft of rigid setae), this large basal tubercle shows a small basal protuberance (giving the impression of a bifid tubercle but with one of the sides aborted); ventral surface and outer margin of tusks with small rounded protuberances on the extremely hard cuticle; dorsal surface of tusks with numerous setae and with a small basal tubercle; this small dorsal tubercle is easily seen without dissecting the mandible and gives an additional point of articulation between the mandible and the head capsule (“a” and “b” in Fig. 14I). Incisors and prostheca of both mandibles very reduced in size, molae relatively well developed. Maxillae with a small subtriangular basal membranous “gill” (membranous outgrouth). Thorax. Anterior ring of pronotum (or collar sensu Kluge 2004) short (ca. 1/4 the length of posterior ring), anteriorly projecting as spines on lateral corners; posterior ring longer, ring-like. Legs (Fig. 15A–D, F–G). Leg I (Fig. 15A–B): femora very wide, well developed, with a double ventro-basal row of long filtering setae; tibio-tarsus (fused, but fusion line distinguishable) with 3 rows of filtering setae (2 on dorsal “face” and 1 on inner margin), tarsus slightly and bluntly projecting apically (arrow in Fig. 15B); tarsal claw relatively large and stout with a row of marginal denticles (Fig. 15G). Leg II (Fig. 15C): smaller, with thinner femora, with scattered long setae, mostly basally and along hind margin; tibia and tarsi with row of long setae on outer (dorsal) margin, ventrally with many stout spines on apical half, with a distal brush of thick setae (arrow in Fig. 15C); tarsal claw relatively small, without denticles. Leg III (Fig. 15D, F): as leg II except larger and with anterior margin of femur densely covered with thick setae, and posterior margin roundly expanded at apex bearing a group of stout acute spines (Fig. 15F); tibia without distal brush. Coxae I and II directed ventrally, coxae III directed laterally. Abdomen. Gill I reduced in size, double, both portions subequal in length and width. Gills II–VII well developed, ventral portion smaller than dorsal portion; tergum X with well developed ventral spine on posterior margin (not visible dorsally, Fig. 15E). Caudal filaments short (curved in mature nymphs) with whorls of stout spines and simple setae at joinings.

**Figure 19.**
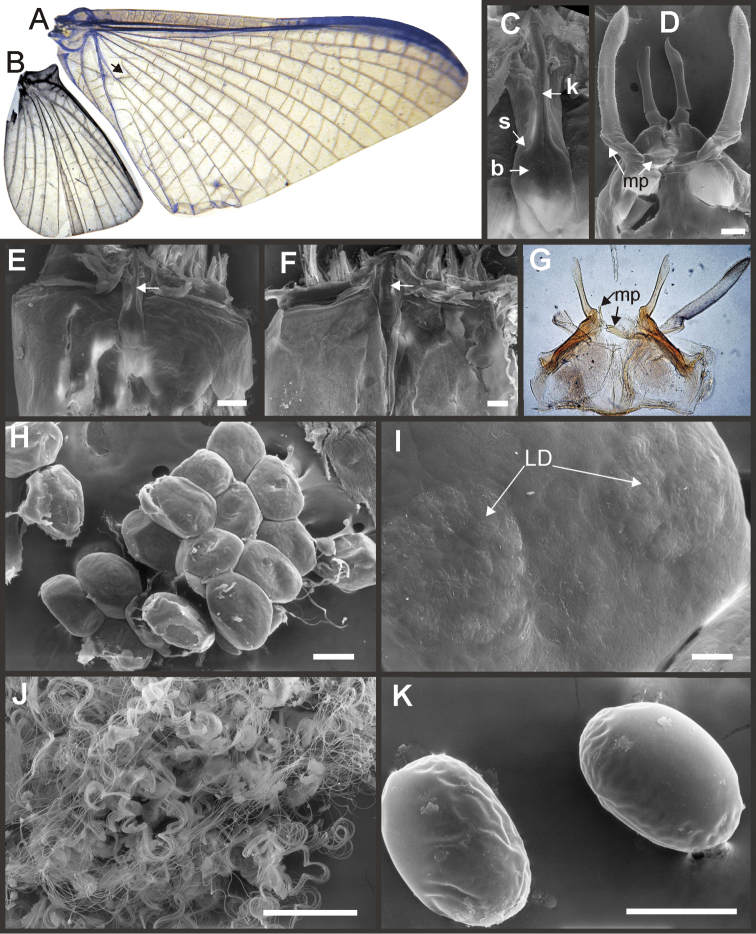
*Povilla* spp, SEM and light microscope photographs. *Povilla adusta*: **A** male forewing **B** male hind wing **C** detail of keel on female abdominal sternum 8 (b = base, k = keel, s = socket) **D** male genitalia (mp = median remnant of styliger plate) **E–F** female abdominal sternum 8 (general view) **G** male genitalia (median remnant of styliger plate partially broken and detached from right pedestal) **H–I** eggs and detail of chorion (LD = large disks). Povilla cf. heardi: **F** female abdominal sternum VIII **J** filaments surrounding the eggs inside female abdomen **K** eggs. Scale bar = 100 µ, except Figure 1 (10 µ).

**Figure 20.**
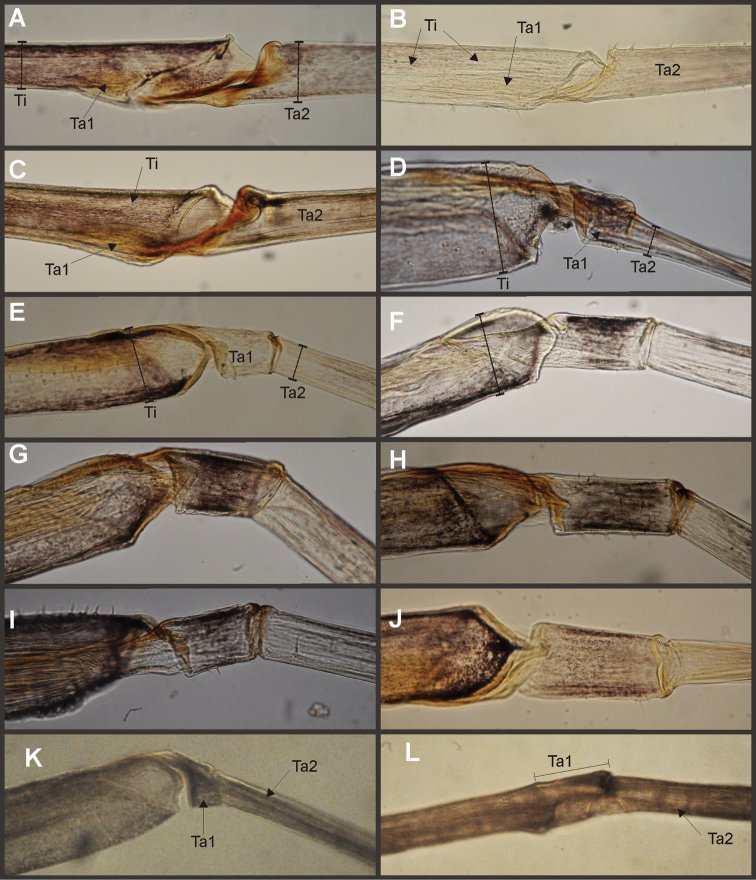
Foreleg of male imago, detail of articulation between tibia and tarsus: **A** *Asthenopodes picteti* **B** *Asthenopodes chumuco* **C** *Asthenopodes traverae* **D** *Hubbardipes crenulatus* **E** *Priasthenopus gilliesi* **F** *Asthenopus angelae* **G** *Asthenopus curtus* **H** *Asthenopus guarani* **I** *Asthenopus hubbardi* **J** *Asthenopus magnus*; **K** *Povilla adusta* **L** *Ephoron* sp. Abbreviations: Ti = tibia; Ta1 = tarsal segment 1; Ta2 = tarsal segment 2.

#### Distribution.

Amazonas and Parana biogeographic subregions (Argentina, Bolivia, Brazil, Colombia, Ecuador, Peru).

#### Discussion.

The genus *Asthenopus* has been distinguished by means of the following characters (Domínguez 1988): 1) ratio foreleg/FW male: 3/5–4/5; 2) male foretarsus 2.5 times longer than foretibia; 3) foretarsal segment 2 similar to the others, and 2/3 the length of tibia); 4) ratio length of Rs stem/fork to margin 1/4 (or fork Rs at 2/10 from base to margin); 5) cubital intercalaries slightly diverging toward hind margin, ICu_2_ and ICu_1_ basally fused to CuA by cross veins; 6) ICu_2_ ending at anal margin or in the tornus, 7) marginal intercalary veins absent; 8) forceps ratio width/length: 1/7; 8) penes robust on basal 2/3; 9) MA fork base to margin 10/100; 11) IMP–MP_1_ not fused basally; 12) MP_2_-IMP similar in length, not fused; 13) foretarsal claws of male not so expanded distally (as in *Asthenopodes*). Our phylogenetic analyses only recovered some of these character states as synapomorphies of this genus (see diagnosis and Appendix 2). The proposal of Domínguez (1988) and Hubbard & Domínguez (1988) concerning the intermediacy of *Priasthenopus gilliesi* with respect to *Asthenopus curtus* and *Asthenopodes picteti* is in concordance with our results. *Priasthenopus gilliesi* resulted sister to the *Povilla*-*Asthenopus* clade, presenting some plesiomorphic character states shared with *Asthenopodes*.

#### Key to the species of *Asthenopus*

Male

**Table:** 

1	Penile lobe (distad to basal thumb) with a similar width along its length, basal thumb separated by a wide furrow (Figs 17A–C, E–F); forceps very stout (ratio length/ basal width = 4.7–6.0)	**2**
–	Penile lobe (distad to basal thumb) wider basally, basal thumb fused to penile lobe (Fig. 17D,G); forceps relatively slender (ratio length/ basal width = 6.2–7.0)	**4**
2	Apical spine of penes long and acute (Fig. 17A–B)	***Asthenopus curtus***
–	Apical spine of penes short (Fig. 17C, E–F)	**3**
3	Penes long, apical spine slightly marked, median remnant of styliger plate projecting laterally (Fig. 17E–F)	***Asthenopus guarani***
–	Penes short, apical spine well marked, median remnant of styliger plate normal (Fig. 17C)	***Asthenopus hubbardi***
4	FW 9.5–10.1; penile lobe strongly widened basally (ratio length / basal width = 2.9, Fig. 17G) and with a small distal indentation near apical spine (arrow in Fig. 17G)	***Asthenopus magnus***
–	FW 7.0–9.5 mm; penile lobe not so wide at the base (ratio length / basal width = 4.0–5.0, Fig. 17D); without apical indentation as above	***Asthenopus angelae***

Female and eggs of *Asthenopus* species are strongly similar. They may be identified by comparison with co-occurring males. Nevertheless the eggs extracted from female adults or mature nymphs may be keyed as follows:

**Table:** 

1	Disk like structures on the equatorial area relatively well separated from each other, separation about 0.6 or more of maximum width of a disk (Fig. 18B, C, E)	**2**
–	Disk like structures on the equatorial area almost touching each other, maximum separation about 0.3 or less of maximum width of a disk (Fig. 18A, D)	***Asthenopus curtus* / *Asthenopus hubbardi***
2	With a group of 2–3 very small disks beneath each disk like structure (Fig. 18C)	***Asthenopus guarani***
–	Only smooth chorion below the disk like structures (Fig. 18B, E)	***Asthenopus angelae* / *Asthenopus magnus***

Nymphs (only 3 species known, almost undistinguishable, the characters below should be confirmed with the study of more material)

**Table:** 

1	On the inner margin of left mandibular tusk, the space between the subbasal and the submedian tubercles is short and strongly concave (Fig. 14A); right mandible with distal corner of mola strongly protruding	***Asthenopus curtus***
–	On the inner margin of left mandibular tusk, the space between the subbasal and the submedian tubercles is longer and straighter (Figs 14B–C); right mandible with distal corner of mola not strongly protruding	**2**
2	Ratio total length of mandible/mandibular tusk length: 1.59–1.62 (Fig. 14B, F)	***Asthenopus angelae***
–	Ratio total length of mandible/mandibular tusk length < 1.5 (Fig. 14C, E, G)	***Asthenopus magnus*** (only known from Napo, Ecuador) / ***Asthenopus guarani*** (Paraná and Uruguay basins)

### Asthenopus curtus

Taxon classificationAnimaliaEphemeropteraPolymitarcyidae

(Hagen)

Figs 4E , 14A , 16A–B , 17A–B , 18A, G , 20G

Palingenia albifilum var.; Walker 1853: 554.
Palingenia curta Hagen 1861: 304.
Campsurus curtus ; Eaton 1868: 84; Eaton 1883: 40; Ulmer 1921: 240.
Asthenopus curtus ; Eaton 1871: 59; Ulmer 1920c: 107; Ulmer 1921: 240; Lestage 1922b: 142; Ulmer 1942: 105; Traver 1956b: 7; Kimmins 1960: 312; Sattler 1967: 104; Berner 1978: 103; Hubbard 1982a: 270; Domínguez 1988a: 24; Hubbard and Domínguez 1988: 207; Domínguez 1989a: 173 (described as *Asthenopus magnus* sp. n. below); Domínguez et al. 2006: 561.
Campsurus amazonicus Hagen 1888: 230.
Asthenopus amazonicus ; Ulmer 1920c: 107; Lestage 1923: 124; Ulmer 1942: 106; Traver 1950: 606; Traver 1956b: 7; Sattler 1967: 104; Berner 1978: 103.

#### Type material.

Photographs of the type at the British Museum were studied.

**Additional material.** Two male imagos (IBN, slide 480) from COLOMBIA, Amazonas, Leticia, caño km 15, S 4°5'41" − W 69°59'1", 93 m, 11.ii.1999, light trap 4−6 h, E. Domínguez, M.C. Zúñiga & C. Molineri cols.; male imaginal slides (FAMU) from BRAZIL, Amazonas, Careiro Island, Divinopolis, SE of Manaus, 29.vii.1961, E.J. Fittkau; 2 male and 1 female pharate subimagos (IBN642CM-eggs, 643-female, 644-male) from BRAZIL, Amazonas, São Paulo de Olivença, Bom Sucesso, 4.ix.2003, (aprox. S 3°28' − W 68°59').

#### Diagnosis.

*Asthenopus curtus* is the type species of the genus, and is known from adults of both sexes, nymphs and eggs. Nine autapomorphies were recovered in the cladistic analysis, and are useful to diagnose the species (see Appendix 2). The following combination of characters is useful to distinguish *Asthenopus curtus* from the other species of the genus: 1) male FW 10.0, female FW 14.0–18.5; 2) male foreleg length 0.69–0.74 times the length of FW; 3) pronotum width/length ratio: 2.0–2.3 (male), 2.7–3.0 (female); 4) 18–25 marginal intercalary veins present on the entire margin of forewings (Fig. 16A–B), 2–3 times shorter than distance between longitudinal veins in male (not anastomosed), hind wings with marginal intercalaries in at least 4 spaces between main veins; 5) male FW with 0 to 1 crossveins between Rs and MA basal to Rs fork; 6) ratio total length/basal width of forceps 5.4 (Fig. 17A–B); 7) penes very sclerotized, contrasting strongly with the remaining genital parts, apex projecting acutely; a deep furrow separates penis lobe from thumb, median remnant of styliger plate subrectangular without marked projections, pedestals subrectangular and large, outer margin projecting posteriorly on outer margin along forceps base; 8) female sternum VIII with anteromedian keel and reduced sockets as in Fig. 18G; 9) egg ratio maximum width of egg/maximum width of PC 1.1–1.3, cap formed by 3–5 filaments, chorionic plates separated by smooth chorion (Fig. 18A); 10) nymphal mandible: ratio total length of mandible/mandibular tusk length 1.6–1.7; 11) space between the subbasal and the submedian tubercles in inner margin of left mandibular tusks is short and concave (Fig. 14A).

Male imago. Length: body, 8.0–8.7; FW, 10.0; HW, 4.4; foreleg, 7.5; cerci, 33.0–35.0. General coloration yellowish light brown. Head whitish, heavily shaded black dorsally, paler on posteromedian zone of occiput, black shading extending anteriorly on frons as two parallel lines surrounding median ocellus. Antennae pale, slightly shaded gray on dorsum. Thorax. Pronotum yellowish translucent completely shaded gray, darker on anterior ring; paler on two transverse lines, one separating anterior and posterior rings and another more posterior and obliquely transverse; pleurae shaded with black, sternum with a median gray macula. Pronotum width/length ratio: 2.0–2.3. Mesonotum whitish yellow (or brownish in some males) with a black median triangle between posteroscutal protuberances, metanotum similar in color, also shaded black posteromedially; mesopleurae and sterna paler, shaded with black along anterior margin of katepisternum. Legs yellowish white shaded with gray dorsally on all coxae, femora and tibiae; foretarsal segment 1 blackish (Fig. 20G), remaining tarsal segments paler shaded with gray distally, claws grayish thin troughout. Wings (Fig. 16A–B). Membrane hyaline shaded very slightly with brownish near anterior margin and turning whitish translucent towards apical zone of C–Sc areas; veins translucent shaded with brown. Abdomen yellowish white shaded extensively with grayish brown dorsally, darkening very slightly towards rear segments. Sterna pale very slightly shaded gray, shaded stronger on mediolongitudinal line near anterior margin of sterna VIII–IX, this line is blurred posteriorly; a grayish black triangular mark is present at each side of this line, on anterior margin of sterna VIII–IX; sternum X shaded black except medially. Genitalia (Fig. 17A–B): median remnant of styliger plate and pedestals yellowish, forceps whitish translucent shaded gray along outer margin, penes dark orange with whitish base. Caudal filaments whitish, shaded gray at base of terminal filament.

Female adult. Length: body, 10.5–13.2; FW, 14.0–18.5; HW, 5.7; cerci, 5.8–7.0. Morphologically very similar to female adults of *Asthenopus angelae* described in detail in de Souza and Molineri (2012). Here only those characters that differ from the cited description are mentioned. Pronotum almost 3 times wider than long, width/length ratio = 2.7–3.0 (see continuous characters in phylogenetic matrix, Appendix 3). Mesonotum uniformely brownish (cuticular pigmentation), almost without gray markings (dermic pigments). Female sternum VIII with anteromedian keel and reduced sockets as in Fig. 18G. Cercus about half the length of FW, cercus length/FW length: 0.4–0.5.

Eggs (Fig. 18A). Length, 200–220 µ; width, 130–155 µ. Two polar caps (maximum width, 110–120 µ), formed by 3–5 very long coiled threads. Chorionic surface smooth with relatively large subcircular chorionic plates, the plates are regularly spaced and some of them are divided in two or three subequal parts.

Mature nymph. Length of male: body, 9.5–9.7; cercus, 7.0; terminal filament, 5.0. Length of female: body, 17.0; cercus, 8.0; terminal filament, 7.0. Only characters that differ from *Asthenopus angelae* are given here, refer to that description for more detailed information. Head (occipital area) dorsally brownish uniformly shaded with gray. Mouthparts. Left mandibular tusks with a relatively shorter space between the large subbasal tubercle and the smaller subdmedian one, this space is somewhat C-shaped (Fig. 14A). Right mandible with distal corner of mola strongly protruding. Thorax. Mesonotum uniformly brownish (cuticular) without strongly gray-shading on carinae (Fig. 4E). Legs and paraprocts identical to those on Fig. 15.

#### Distribution.

Amazonas River from Leticia (Colombia) to Manaus (Brazil).

#### Discussion.

Much confusion exists in the literature concerning this species. Many authors mention *Asthenopus curtus* but from missidentified material. For example Ulmer (1942) described and illustrated (as *Asthenopus curtus*) a pair of males of *Asthenopus angelae*. The material from Ecuador studied by Domínguez (1988) proved to be a different but related species (*Asthenopus magnus* sp. n.). Berner (1978) synonymized *Asthenopus curtus* with *Asthenopus amazonicus*, showing that the differences between both species were only attributable to sexual dimorphism, but he was working with *Asthenopus angelae* males (de Souza and Molineri 2012). Nevertheless, Berner conclusions were correct given that sexual dimorphism in FW venation is present in both species. As it is impossible to assign any specimen to *Asthenopus amazonicus*, we prefer to treat it as synonym of *Asthenopus curtus*, as Berner proposed. Actually, only one specimen from previous works is positively determined as *Asthenopus curtus*: the type, studied by Eaton (1883) and illustrated by Kimmins (1966). We add here some other records from the Amazonas River: a pair of males from Colombia, some reared nymphs from Brazil and Fittkau’s slides at FAMU. These male imagos show the characteristic genitalia of the holotype of *Asthenopus curtus*, with extremely wide forceps, long penis lobes and slender and very acute apical spines, and the more or less uniform brownish mesothoracic coloration (an exception of this last character are the males from Colombia-Leticia, much paler). The egg of *Asthenopus curtus* (Fig. 18A) is similar to that of *Asthenopus hubbardi* (Fig. 18D), in the shape and relative large size of the disk-like structures that leaves exposed only a reduced surface of smooth chorion. On the contrary the egg of *Asthenopus angelae* presents smaller disk-like structures with a larger surface of smooth chorion among them (Fig. 18B).

As the result of the present study, the female adult, egg, and nymphal stages are described here for the first time. Previous descriptions of female and nymphs in the literature were done from specimens of *Asthenopus angelae* or other species but are not useful to clearly distinguish the species.

### Asthenopus magnus sp. n.

Taxon classificationAnimaliaEphemeropteraPolymitarcyidae

http://zoobank.org/70A79C87-DD63-4371-8085-7BD7B5E55C26

Figs 4C , 14C, E, G, I–J , 15A–D , 16C–D , 17G , 18E–F

Asthenopus curtus , Domínguez 1989: 173; Domínguez et al. 2006: 561 (missidentification).

#### Material.

Holotype (IBN) male imago from Ecuador, Napo Province, Laguna Limon Cocha, 250 m, 6.iv.1984, E. Domínguez col.(aprox. S 0°24' − W 76°38'). Paratypes, same data as holotype, separated in 16 vials including: 1 nymph dissected (parts in alcohol), 2 male imagos (parts on slides: IBN-2–64ED, IBN-2–70ED), 1 male imago (IBN-2–67ED), 2 nymphs/3 exuviae/1 pharate male, 1 pharate male subimago and nymphal cuticle (IBN483CM), 1 male imago (IBN481CM), 3 nymphal exuviae (IBN640CM, IBN641CM), 7 nymphal exuviae (1 at FAMU, 1 at CZNC), 5 female adults (used for SEM); 7 male imagos (used for the description; 1 at FAMU, 1 at CZNC), 20 male subimagos, 10 female adults, 1 male and 8 female subimagos, 10 female adults, 9 female adults (1 at FAMU, 1 at CZNC), 1 female adult (IBN-2–71ED). All the material is deposited in IBN except otherwise indicated.

#### Diagnosis.

*Asthenopus magnus*, known from all the stages, can be distinguished from other species in the genus by the following combination of characters (also see the six autapomorphies in Appendix 2): 1) male FW 9.0–10.1, female FW 16.0–17.5; 2) forelegs of male 0.69–0.73 × the length of FW; 3) pronotum width/length ratio: 1.7–2.4 (male), 2.0–2.2 (female); 4) FW (Fig. 16C) with 6–14 relatively short marginal intercalaries (21–28 in female), hind wings with 2–4 (5–7 in female) marginal intercalaries; 5) male FW with 0 to 2 (2–3 in female) cross veins between Rs and MA basal to Rs fork; 6) forceps relatively slender, ratio length/basal width 6.1–6.2 (Fig. 17G); 7) penes tubular and robust, with well developed thumb, curved ventro-medially, with apex projecting medially as a distal spine, furrow separating penis lobe from thumb reduced; median remnant of styliger plate subrectangular, pedestals subrectangular to subovate, relatively large (Fig. 17G); 8) female sternum VIII with reduced, not distinguishable female sockets, but with a long anteromedian keel (Fig. 18F); 9) eggs (Fig. 18E) ratio maximum width of egg/maximum width of PC 1.2–1.3, cap formed by 4–5 filaments, chorionic plates separated by smooth chorion; 10) nymph, ratio total length of mandible/mandibular tusk length 1.4–1.5; 11) inner margin of left mandibular tusk with subbasal and submedian tubercles well separated (Fig. 14C, E).

Male imago. Length (mm): body, 9.0–10.5; FW, 9.0–10.1; HW, 4.0–4.8; leg I, 6.5–7.4; cerci, 37.0. General coloration yellowish light brown. Head whitish shaded black dorsally on pale median mark on hind margin and along inner margin of eyes; frons pale except paired submedian black lines. Antennae: whitish shaded diffusely with gray on scape and apex of pedicel; length (mm): scape 2.25, pedicel 1.5, flagellum 7.25. Thorax. Pronotum yellowish translucent shaded with black dorsally except at pale median membrane between both pronotal rings, on mediolongitudinal line and along margins; the black shading presents many scattered and small pale spots. Pronotum width/length: 1.7–2.4. Meso- and metanotum yellowish shaded with grayish on carinae, posteromedian triangular mark (on mesonotum), and scutellum (both). Thoracic pleurae and sterna paler, shaded gray on pleural sclerites. Legs yellowish white, shaded gray on all coxae. Leg I shaded gray almost completely, stronger on femur and tibia, paler on tarsal segments (Fig. 20J). Legs II–III shaded with black dorsally on apex of femora and entire dorsum of tibiae, rest pale. Wings (Fig. 16C–D). Membrane hyaline shaded grayish near costal margin on basal half, more whitish apically; all veins translucent completely shaded gray; 1–3 cross veins between MA and R, basad to R stem. Abdomen yellowish white shaded with gray and black dorsally except on median and lateral zones and intersegmental membranes. Median pale areas on terga II–IX are oval and are surrounded by darker pigments, a delicate mediolongitudinal black line is present but sometimes is only visible on tergum IX. Sterna whitish turning yellowish laterally and on sternum IX, shaded gray on paraproct and basally to terminal filament. Genitalia (Fig. 17G): forceps whitish, penes yellowish white. Cerci whitish very slightly shaded with gray.

Female imago. Length (mm): body, 15.5–19.5; FW, 16.0–17.5; HW, 5.2–7.8; cerci, 3.5–5.0. Pronotum width/length: 2. Morphologically very similar to *Asthenopus curtus* and *Asthenopus angelae*, the last is described elsewhere (de Souza and Molineri 2012). Color pattern similar to male but more strongly marked, exceptions follows: pale median mark on occiput longer, reaching median ocellus; wing membrane tinged with yellowish near costal margin and base, all veins shaded brown; shading on abdominal terga more extended, shaded widely gray except medially, tergum VIII–IX (sometimes also II–VII) with a black line in the pale median zone; medial margins of gill sclerites on abdominal sterna II-VII and lateral margins of sterna VIII–IX grayish; Sternum VIII with keel as in Fig. 18F. Cerci yellowish, 0.3–0.4 the length of FW.

Eggs (Fig. 18E). Length, 250–285 µ; width, 145–160 µ. Two polar caps (maximum width, 115–135 µ), formed by 4–5 very long coiled threads. Chorionic surface smooth with relatively large subcircular chorionic plates, the plates are regularly spaced and some of them are divided in two or three subequal parts.

Nymphs. Length of male (mm): body, 10.0–11.0 mm; cerci, 7.0–8.0; terminal filament, 5.0–5.5. Length of female (mm): body, 17.0–20.0 mm; cerci, 4.0–5.0; terminal filament, 5.0. General coloration brownish. Head (Fig. 14I–J) yellowish brown extensively shaded with grayish brown, darker on a band between ocelli, occiput with a profuse netted grayish pattern, except on paler median zone and along inner margin of eyes; paler areas also present basally to antennae and around median ocellus. Antennae yellowish white, length (mm): scape (0.5), pedicel (0.28), flagellum (2.0). Mandibular tusks with relatively large space between large basal tubercle and smaller subdistal tubercle, not C-shaped as in *Asthenopus curtus* but in the form of broad “C” or bracket-shaped (Fig. 14C, E, G). Thorax. Anterior ring of pronotum (collar) blackish; posterior ring shaded gray except on mediolonitudinal line and a pair of sublateral pale marks; pronotal membranes whitish. Mesonotum brownish, lighter toward apex of wingpads. Thorax ventrally whitish. Legs (Fig. 15A–D) whitish yellow shaded with gray on coxae, apex of femora and along tibiae; foretarsal claw with 20 (male) to 31 (female) denticles in a marginal row, increasing in size distally; distal region of hind femur with a group of ca. 60 (male) to ca. 100 (female) stout spines. Abdomen. Terga shaded gray dorsally, except on paler median band, a thin black median line is present inside this pale area on tergum IX–X; lateral zones of terga below gills, pale. Gills whitish almost completely shaded with gray, darker on outer (exposed) zones. Sterna whitish shaded with gray on lateral margins of sterna VIII–IX, and with grayish black on paraprocts (Fig. 15E). Caudal filaments yellowish.

#### Etymology.

From Latin “magnus” meaning “large”, noun in apposition. The name alludes to the general size of the individuals, mainly the female adults.

#### Distribution.

Only known from the type locality in Napo (Ecuador).

#### Discussion.

The type series described here as *Asthenopus magnus* were previously treated as *Asthenopus curtus* (Domínguez 1989: 173; Domínguez et al. 2006: 561), and used to record the latter species in Ecuador. As a result of our study we found some characters distinguishing these specimens as a new species, and thus *Asthenopus curtus* is no more considered to be present in that country. Nymphs and adults of both sexes were associated by nymphal exuviae and adults caught at the moment of emergence.

### Asthenopus hubbardi sp. n.

Taxon classificationAnimaliaEphemeropteraPolymitarcyidae

http://zoobank.org/1904C509-C6F5-45F3-9623-F7256CA7D022

Figs 16E–F , 17C , 18D , 18H , 20I

#### Material.

holotype male imago (slide IBN479CM) from Colombia, Amazonas, Puerto Nariño, Loreto Yacu, S 3°44'26" − W 70°27'19", 5.ii.1999, luz 6−8 h, M.C. Zúñiga, E. Domínguez and C. Molineri cols.; and paratypes: 1 female imago (slide IBN574CM) same data as holotype; and 1 male imago (slide IBN605CM) from Colombia, Amazonas, Puerto Nariño, Lago Tarapoto, S 3°47'47" − W 70°25'17", 4.ii.1999, light trap 18–20 hs, M.C. Zúñiga, E. Domínguez and C. Molineri cols. Holotype deposited in MUSENUV, paratypes in IBN.

#### Diagnosis.

*Asthenopus hubbardi*, known from adults of both sexes, can be distinguished from the other species in the genus by the following combination of characters (one autapomorphy is listed in Appendix 2): 1) male FW 7.8–9.2 (Fig. 16E–F), female FW 13.0; 2) forelegs of male 0.57–0.65 × the length of FW, apex of foretibia with stout spines (Fig. 20I); 3) pronotum width/length ratio: 2.1 (male), 2.7 (female); 4) FW with 4–14 relatively short marginal intercalaries (ca. 20 in female), hind wings without marginal intercalaries (present in all spaces in female); 5) FW with 2–3 cross veins between Rs and MA basal to Rs fork, in both sexes; 6) forceps relatively slender, ratio length/basal width 4.7–6.0 (Fig. 17C); 7) penes tubular and robust, furrow separating penis lobe from thumb well marked, median remnant of styliger plate subrectangular, pedestals subrectangular to subovate, relatively large (Fig. 17C); 8) female sternum VIII with reduced female sockets, anteromedian keel present (Fig. 18H); 9) eggs ratio maximum width of egg/maximum width of PC 1.1–1.3, cap formed by 3–8 filaments, chorionic plates almost contiguous (Fig. 18D).

Male imago. Length (mm): body, 7.0–7.1; FW, 7.8–9.2; HW, 3.3–4.0; leg I, 5.1–5.2; cerci, 22.0–23.0. General color whitish brown. Head whitish shaded black dorsally except thin line along hind margin, with a pair of blackish short lines anteriorly to median ocellus. Antennae whitish shaded gray at margins of scape. Thorax. Pronotum ratio width/length: 2.1. Pronotum yellowish translucent widely shaded black, except on pale transversal line between anterior and posterior rings, and on mediolongitudinal line of posterior ring, and posterolateral oblique dashes. Meso- and metanotum whitish yellow shaded black on carinae and margins, also shaded on black on posteromedian triangular zone. Legs whitish shaded gray on dorsum of foreleg, all coxae, and on legs II–III on apex of tibiae and dorsum of tarsi; foretarsal segment 1 is shown in Fig. 20I. Wings (Fig. 16E–F) membrane hyaline except apically whitish on C and Sc areas, veins translucent yellow turning hyaline distally, except basal 2/3 of veins Sc and R_1_ yellowish; 2 cross veins between R and M basally to R stem. Abdomen whitish shaded almost completely with brownish gray except at pale intersegmental membranes, oblique dashes on lateral zones of terga I–VIII, submedian pale spots on anterior margin of terga II–IX, and single posteromedian pale spot on terga III–IX. Genitalia (Fig. 17C) whitish except penes yellowish. Caudal filaments whitish, slightly shaded gray on basal segments of cerci.

Female imago. Length (mm): body, 10.5; FW 13.0; HW, 5.0, cerci 3.8. Similar to male, shaded dorsally more uniformly and markedly. Pronotum ratio width/length: 2.7. FW with 4 cross veins between R and M basal to R stem (none of them just below fork). Abdominal sternum VIII with keel as in Fig. 18H. Cerci yellowish turning whitish apically; ratio length cercus /FW = 0.3.

Eggs (Fig. 18D). Length, 210–245 µ; width, 135–150 µ. Polar caps (maximum width, 110–135 µ) formed by 3–8 long coiled threads. The chorionic plates are almost contiguous, leaving a reduced smooth chorionic surface among them (Fig. 18D).

#### Etymology.

The species is named for Mike Hubbard who has contributed significantly to the understanding of mayflies throughout the world.

#### Distribution.

Two near localities in the Amazonas River from Colombia.

#### Discussion.

This species is very similar to *Asthenopus angelae*, and both were collected in the same lightraps in Colombia, nevertheless they can be separated because *Asthenopus hubbardi* shows translucent veins in the wings (brownish in *Asthenopus angelae*); in most specimens a cross vein is present just below R fork in *Asthenopus angelae* (more basal or distal in *Asthenopus hubbardi*), and the penes are shorter and well separated from the basal thumb in *Asthenopus hubbardi* (similar to *Asthenopus curtus*). *Asthenopus hubbardi* is further characterized because foretibia (Fig. 20I) presents strong marginal spines (weak or absent in *Asthenopus curtus*).

### Asthenopus guarani sp. n.

Taxon classificationAnimaliaEphemeropteraPolymitarcyidae

http://zoobank.org/E2D5EC68-E2C3-4894-B4FC-452D3320F105

Fig. 16G–I , 17E–F , 18C

#### Type material.

Holotype male imago (slide IBN473CM) from Argentina, Corrientes, Parque Nacional Mburucuya, Selva Misionera (sector 6), luz, 29.iii.2001, F. Navarro col.

**Additional, non-type material.** One reared female subimago (IBN524CM) and nymphal cuticle (IBN639CM) from Argentina, Corrientes, Laguna Brava, 27.i.1977, Poi de Neiff col. (egg in Fig. 18C extracted from this female); and 1 male imago (IBN638CM) from Uruguay, Salto, near Salto Grande, frente a Isla del Paredón, 20−21.I.1975, luz 22hs; 4 male and 10 female imagos from Brazil, São Paulo, Luiz Antonio, 10.iv.1991, C.G. Froelich col. (MZSP); 1 male and 4 female imagos from Brazil, São Paulo, Luiz Antonio, Reserva Jatai, 9.iv.1990, C.G. Froelich col. (MZSP).

#### Diagnosis.

*Asthenopus guarani*, known from all stages, can be distinguished from the other species in the genus by the following combination of characters (seven autapomorphies are detailed in Appendix 2): 1) male FW 8.0–9.0 mm (Fig. 16G–H), female FW 16.0 mm; 2) Ratio FW/foreleg length 1.4–1.8; 3) pronotum width/length ratio: 2.15 (male), 3.1 (female); 4) male FW with 4–6 marginal intercalaries (24–26 in female), slightly shorter than the separation of main veins, HW (Fig. 16I) with 2–4 marginal intercalaries (8–9 in female); 5) male FW with 0–2 cross veins between Rs and MA basal to Rs fork (3 in female); 6) forceps relatively slender, ratio length/basal width 4.8–5.8 (Fig. 17E–F); 7) penes tubular and slender, furrow separating penis lobe from thumb well marked; median remnant of styliger plate with lateral rounded lobes as in Fig. 17E). 8) female sternum VIII with reduced, not distinguishable female sockets, but with a long anteromedian keel; 9) eggs (Fig. 18C) ratio maximum width of egg/maximum width of PC 1.2, cap formed by 3–8 filaments, disk-like structures well separated by smooth chorion, with 2–3 small disks beneath each larger disk (Fig. 18C); 10) nymph, ratio total length of mandible/mandibular tusk length 1.4; 11) inner margin of left mandibular tusk with subbasal and submedian tubercles well separated (similar to Fig. 14C, E).

Male imago. Length (mm): body, 6.5–9.0; FW, 8.0–9.0; HW, 3.6–4.0; foreleg, 5.1–5.4; cerci, 25.4–28.0. General coloration yellowish white. Head whitish shaded with black dorsally except on hind margin and posteromedian pale mark; frons pale shaded with a pair of black submedian longitudinal lines; venter of head pale. Antennae whitish shaded with gray on scape; length (mm): scape 1.75, pedicel 1.25, flagellum 7.5. Thorax. Pronotum whitish translucent, shaded black on anterior ring and lateral margins; posterior rings shaded gray, with darker mediolongitudinal line. Meso- and metanotum yellowish white, shaded gray on scutellum; sterna pale shaded gray only on mesokatepisternum. Legs whitish shaded gray on coxae. Foreleg completely shaded gray, paler on base of tarsal segments 2–5 (Fig. 20H). Middle and hind legs shaded gray on apical half of femora and apical 2/3 of tibiae, tarsi and claws translucent. Wings (Fig. 16G–I). Membrane of both wings hyaline, except shaded with light gray at base of costal margin in both wings and whitish on the apex of costal margin of FW; veins translucent, except Sc and R_1_ whitish, all veins shaded slightly gray but more diffusely toward apex. Abdomen whitish, terga uniformly shaded gray, paler laterally and anteriorly to each tergum; sterna whitish. Genitalia (Fig. 17E–F) whitish, penes yellowish white. Cerci whitish translucent.

Female subimago. Length (mm): body, 12.5; FW, 16.0; HW, 6.5; cerci broken off and lost. General coloration orangish yellow shaded widely black. Head dorsally blackish except medial line on occiput, and anteriorly to median ocellus. Thorax. Pronotum width 2.5 mm, total length 0.8 mm; cream shaded black on thin anterior ring, with gray on posterior ring, membranes whitish. Mesothorax orangish yellow with gray markings. Wings translucent whitish, veins whitish except basal half of C, Sc and R yellowish. Abdomen uniformely shaded gray dorsally, except on pale medial line. Sternum VIII with long and thin anteromedian keel. Base of caudal filaments whitish (rest broken off and lost).

Eggs (Figs 18C). Length, 160–200 µ; width, 100–160 µ. Polar caps (max. width 130 µ) formed by 3–8 long coiled threads. The disk-like structures are circular and entire (not partitioned as other species), very well separated by smooth chorion, and when removed, a group of 2–3 very small disks are visible beneath (Fig. 18C).

Nymph (cuticle from reared female described above). Length (mm): body, 16.5 mm; cerci and terminal filament, 7.0 (both broken at apex). Antennae broken off and lost. Mouthparts. Mandibular tusks with relatively large space between the large basal tubercle and smaller subdistal tubercle, not C-shaped (similar to Fig. 14C, E, G). Legs. Foretarsal claw with a row of 26–29 denticles. Apex of hind femur with a group of 90–100 stout acute spines.

#### Etymology.

The name refers to one of the etnic groups inhabiting the area where the specimens were collected.

#### Distribution.

Argentina (Corrientes), Brazil (Sao Paulo), Uruguay (Salto).

#### Discussion.

This species is very distinctive, not only by the long and slender penis lobe, but also because of the presence of relatively long marginal intercalary veins (in male FW and HW). The reared female subimago is tentatively associated with the male, because of similarity in coloration and shared distributional range. Eggs extracted from this female show also some differences from the other known in the genus, mainly the larger extent of smooth chorion around the plates and the presence of small disks below the larger ones (Fig. 18C).

### Asthenopus angelae

Taxon classificationAnimaliaEphemeropteraPolymitarcyidae

de Souza & Molineri

Figs 4D , 14B, F , 15F–G , 16J–K , 17D , 18B

#### Material

(see de Souza and Molineri 2012). Additional records: 13 nymphs (including 3 male and 1 female pharate subimago) from BRAZIL, Amazonas, Codajás, Urucurizinho, Lago Cuxuará (A10-lago Aquapi 14), 15.ix.2003 (slides IBN645CM and IBN646CM) (aprox. S 3°55' − W 62°3'); and 1 pharate male subimago from BRAZIL, Amazonas, São Paulo de Olivença, Bom Sucesso (A02-agapito), 9.iv.2003 (slide IBN606CM) (aprox. S 3°28' − W 68°59'). Deposited in INPA (4 nymphs), CZNC (6 nymphs), IBN (3 nymphs).

#### Diagnosis.

Only one autapomorphy was recovered in our analysis for *Asthenopus angelae*, a change in the ratio A (total length forceps)/E(basal width) from 6.2 to 6.5–7.1 (i.e., forceps become slightly slender). This species can be recognized by the following combination of characters: 1) FW size male 7.0–10.0 mm (Fig. 16J–K), female 12.0–17.0 mm; 2) ratio FW/foreleg length 1.4–1.6; 3) pronotum width/length ratio: 2.0–2.2 (male), 2.2–2.9 (female); 4) 5–11 imv present in male FW, relatively short and poorly anastomosed; 5) male FW with 1–4 cross veins between Rs and MA basal to Rs fork; 6) ratio total length/basal width of forceps 6.5–7.1 (Fig. 17D); 7) penes tubular, with well developed thumb, curved ventro-medially, with apex projecting acutely, furrow separating penial lobe from thumb reduced or absent (Fig. 17D), pedestals subrectangular to subovate, relatively large; 8) female sternum VIII with anteromedian keel and reduced sockets similar to other species; 9) eggs (Fig. 18B) with 3–5 threads on polar caps, ratio maximum width of egg/maximum width of PC 1.1–1.3, chorion with smooth area around rounded disks; 10) nymph, ratio total length of mandible / mandibular tusk length 1.6; 11) space between the subbasal and the submedian tubercles relatively long and straight (Fig. 14B).

#### Distribution.

Argentina, Bolivia, Brazil, Colombia and Peru.

#### Discussion.

This species was recently described from all the stages (de Souza and Molineri 2012), and in our analysis it appears as sister to *Asthenopus magnus*, but with relatively low support. In the original description, nymphs and female adults were not distinguished from *Asthenopus curtus*, because of lack of characters. We proposed (above in diagnosis) some characters that should be checked and confirmed with the study of more material. We suggest that specific identification of this and the other species of the genus should be confirmed when possible with the study of male genitalia. Peru was mentioned by de Souza and Molineri (2012) in the list of material but not in the distribution, so here it is added in that section.

**Figure 21.**
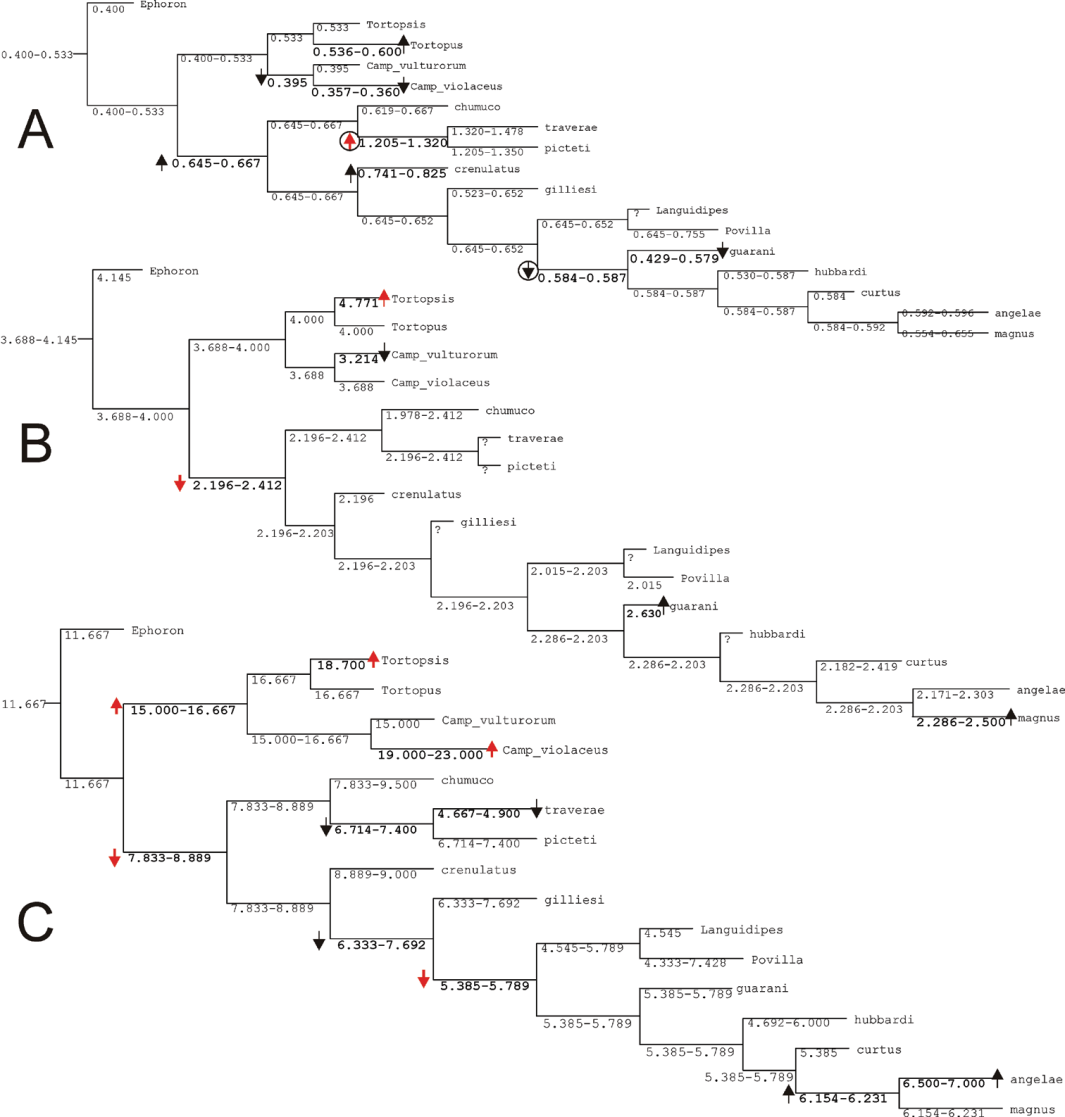
Optimization of selected continuous characters. **A** Char. 1 (1.416 steps) Ratio length second foretarsal segment/foretibia **B** Char. 27 (3.791 steps) Nymph, width of tusk as ratio between length of tusk (T) / width of tusk at the base (W) **C** Char. 13 (23.811 steps) Ratio A(total length forceps)/E(basal width). Arrows indicate increments or decrease in the characters (up and bottom directed arrows, respectively); red arrows indicate a marked change for the node.

**Figure 22.**
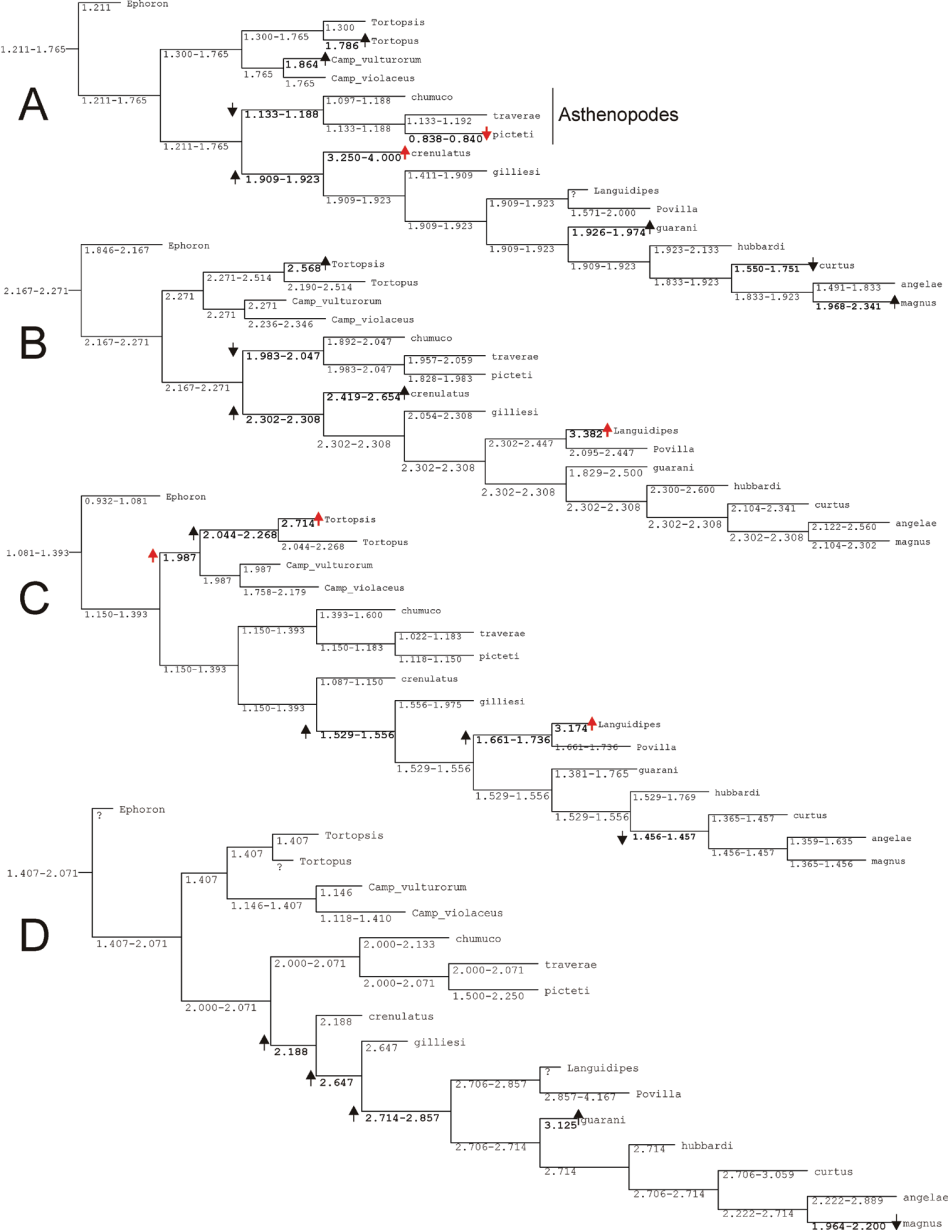
Optimization of selected continuous characters. **A** Char. 0 (3.873 steps) Ratio subapical width of foretibia/subbasal width of tarsal segment 2 **B** Char. 5 (2.344 steps) Ratio length FW/HW **C** Char. 2 (4.148 steps) Ratio FW/foreleg length **D** Char. 18 (2.225 steps) female prothorax, width/length. Arrows indicate increments or decrease in the characters (up and bottom directed arrows, respectively); red arrows indicate a marked change for the node.

**Figure 23.**
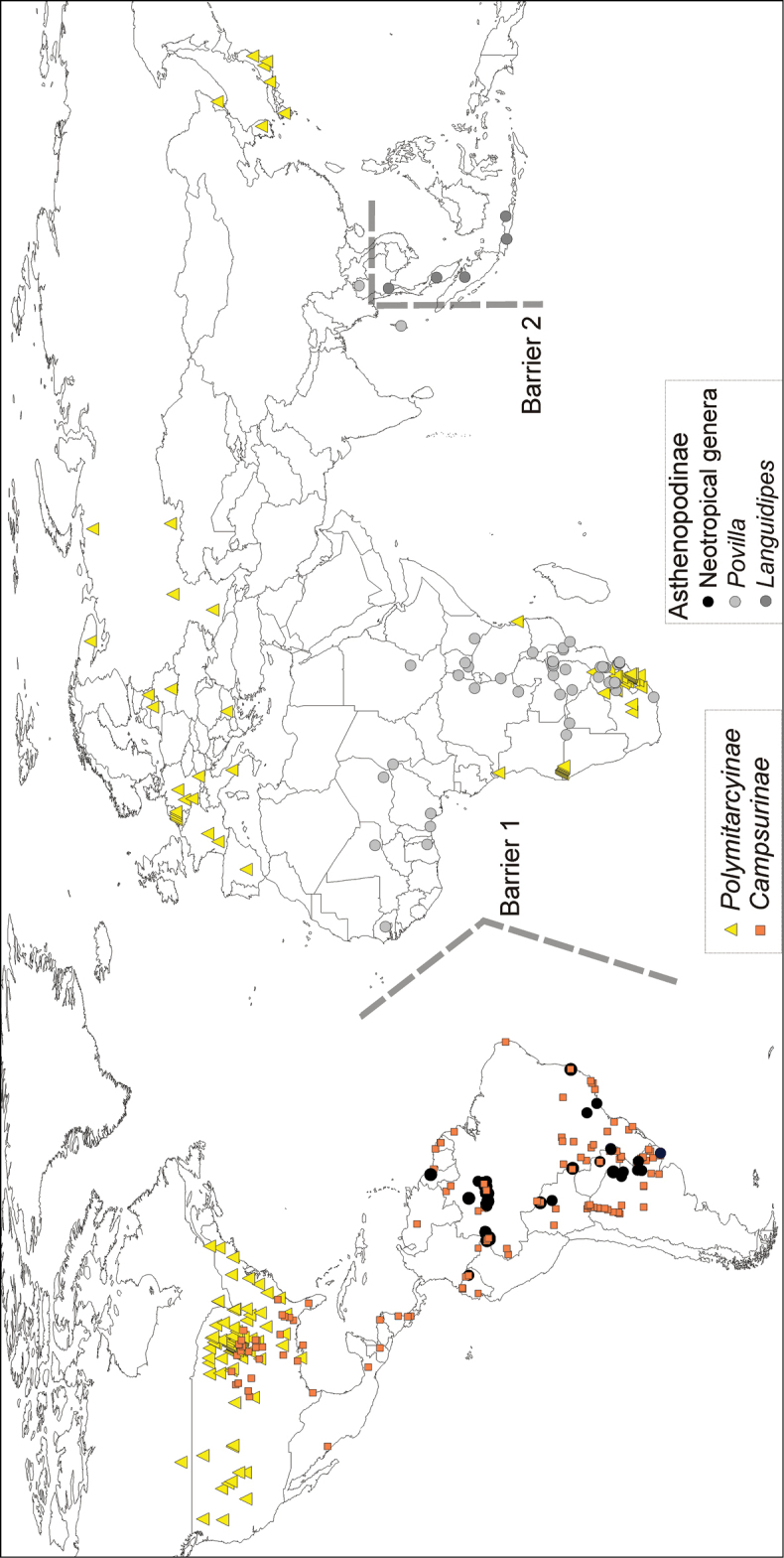
World map indicating the distribution of the three subfamilies in Polymitarcyidae. Additionally, barriers 1 (separating the genus *Asthenopus* vs *Povilla* + *Languidipes*) and 2 (separating *Povilla* from *Languidipes*) found in the biogeographical analysis are marked.

**Figure 24.**
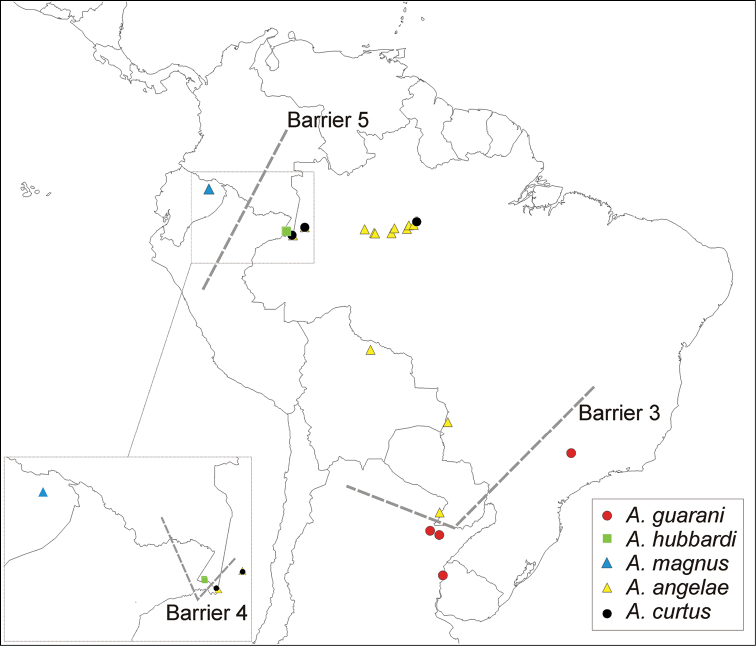
Map of South America (in part) showing the distribution of *Asthenopus* species. Barrier 3 found in the biogeographical analysis separates *Asthenopus guarani* from the remaining species in the genus. Barrier 4 (see detail at left bottom) separates *Asthenopus hubbardi* from the rest; and Barrier 5 separates *Asthenopus magnus* from its sister *Asthenopus angelae*.

**Figure 25.**
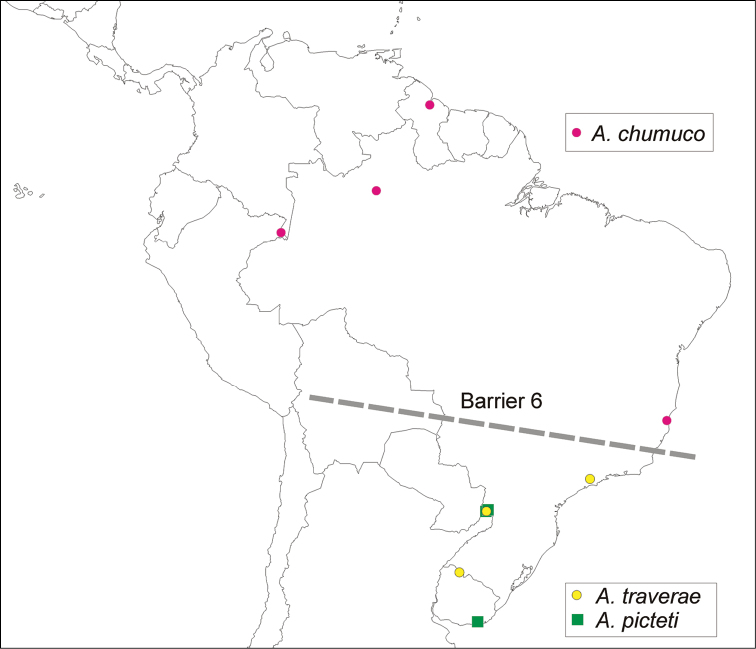
Map of South America (in part) showing the distribution of *Asthenopodes* species. Barrier 6 found in the biogeographical analysis separates *Asthenopodes chumuco* from the remaining species in the genus.

## Supplementary Material

XML Treatment for Hubbardipes

XML Treatment for Hubbardipes crenulatus

XML Treatment for Priasthenopus

XML Treatment for Priasthenopus gilliesi

XML Treatment for Campsurus paraquarius

XML Treatment for Asthenopodes

XML Treatment for Asthenopodes picteti

XML Treatment for Asthenopodes traverae

XML Treatment for Asthenopodes chumuco

XML Treatment for Asthenopus

XML Treatment for Asthenopus curtus

XML Treatment for Asthenopus magnus

XML Treatment for Asthenopus hubbardi

XML Treatment for Asthenopus guarani

XML Treatment for Asthenopus angelae

## Appendix 1

List of continuous characters and their definitions. Characters were scored from male imagos unless otherwise indicated.

0}Ratio subapical width of foretibia/subbasal width of tarsal segment 2. The width of the tibia was measured before the first tarsal segment if fused, as in *Asthenopodes* species (Figure 20).1}Ratio length foretarsal segment 2/foretibia. Length of foretibia includes the reduced first tarsal segment if fused (in *Asthenopodes* species).2}Ratio FW/foreleg length. Fore and hind wings length was taken from the base of the costal brace to the apex. The length of male foreleg does not include the coxa; it was taken from the base of the trochanter to the apex of the claws.3}Ratio FW/cercus length. Cerci were measured only when complete (i.e., with a thinner and setose short portion in the apex).4}FW ratio length/width. Width of FW refers to the maximum width (located near the Cu sector).5}Ratio length FW/HW6}FW, number of imv (intercalary marginal veinlets) from ICuA_1_ to R_1_. The variation in wing venation (number, length and degree of anastomosis) shown by these veinlets is difficult to formalize. For the “number of imv” (intercalary marginal veinlets), only those reaching the hind margin (attached and dettached) were counted, from ICuA_1_ to R_1_.7}FW, marginal connectivity (n°of connections among imv/n°of imv). The anastomosis or “marginal connectivity” of these veinlets was calculated as a ratio between the total number of connections among the imv and the total number of imv counted for previous character.8}Number of crossveins between R and M (e.g., arrows in Figure 8H), basal to R fork9}Ratio marginal length between main longitudinal veins/imv length (mean of all values in a wing). The length of the imv in relation to the distance between the apices of main longitudinal veins is represented as the mean value of all the ratios measurable in the FW (i.e., the number of marginal interspaces between main veins, from ICuA_1_ to R_1_, frequently about 14 interspaces).10}FW male, Rs stem length/Rs from fork to margin. The measures were taken as straight lines; in the case of the distal measure, from fork of Rs to apex of IRs.11}Prothorax width/length. Prothorax width and length were taken in dorsal view, and are maximum values (i.e., widest and longest parts were measured respectively, including membranes).12}Ratio median remnant of styliger plate, maximum width/length at middle13}Ratio A (total length forceps)/E (basal width). A = total length of forceps, also as a straight line (e.g., line “g” in Figure 7A). E = basal width of forceps, taken as indicated by line “e” in Figure 7A.14}Ratio L (length of penile lobe)/B (length of inner thumb). L = total length of penes, taken as a straight line from the base to the apex of the lobe (e.g., line “a” in Figs 7A, 8A, 12B). B = length of thumb of penes, measured as a straight line in the medial margin (line “b” in the same figures)15}Ratio L /C. L as defined above; C = basal width of penis lobe (line “d” in Figs 7A, 8A, 12B and others)16}Ratio L /Z (curvature in v.v.). L as defined before; Z = a measure intended to quantify the curvature of the penis lobe, it was taken in v.v. as the distance between two parallel lines as indicated by line “z” in Figure 12B.17}Penes, ratio C (basal width)/D (subapical width). C as defined before; D = subapical width of penis lobe (line “c” in Figs 7A, 8A, 12B)18}Prothorax width/length (female adult)19}Cercus/FW length (female adult)20}Egg, ratio maximum length (including polar caps)/maximum width21}Egg, ratio maximum with/maximum width of polar cap (uncoiled)22}Egg, polar cap, ratio maximum width/maximum length23}Egg, polar cap, number of threads forming each cap24}Nymph, total length of mandible/mandibular tusk length. Asthenopodinae is defined by stout tusks, and Campsurinae by slender ones, as this is subjective and difficult to score in a matrix we use continuous variation in the length of the tusk (T in Figure 5B) with respect to the total length of the mandible (L in Figure 5B).25}Nymph, position of the large inner tubercle along the longitudinal axis of the mandible. This is formalized as the ratio: total length of mandible (L in Figure 14A) / length from base of the mandible to the transverse line bisecting the tubercle (t1 in Figure 14A).26}Nymph, position of the small and subdistal inner tubercle along the longitudinal axis of the mandible. This is formalized as above, ratio: total length of mandible (L in Figure 14A) / length from base of the mandible to the transverse line bisecting the tubercle (t2 in Figure 14A).27}Nymph, width of tusk as ratio between length of tusk (T) / width of tusk at the base (W). In those cases where a large tubercle is present, the width does not include it (see Figure 14A).

Discrete characters

28}Male foretarsal segment 1: 0 = distinct (Fig. 20E–K); 1 = fused with tibia (Fig. 20A–C, L); 2 = fused with second tarsal segment (Fig. 20D). Most species present a distinct tarsal segment 1 in male foreleg, a strong articulation is located between the apex of the tibia and this first tarsal segment (Fig. 20E–K). In *Asthenopodes* the first tarsal segment is completely fused with the tibia, and thus the articulation is located between the fused tibiotarsus and the second tarsal segment (Fig. 20A–C). In the unique *Hubbardipes crenulatus* the first tarsal segment, while distinct, shows an incipient fusion with the second tarsal segment (Fig. 20D).29}Male foretarsal segment 1, form: 0 = subquadrate; 1 = subrectangular; 2 = subovate.30}Foretarsal segment 5, apex 0 = blunt or with a transverse ridge; 1 = trilobed.31}Apex of male foretarsal claws: 0 = not or slightly expanded (Figure 7C); 1 = strongly expanded (apex 3 times wider than stalk, Fig. 13E–F).32}Legs II and III (male): 0 = complete, functional at least in subimago; 1 = complete but weaker, non functional; 2 = tarsal segments not discernible, distorted; 3 = last segment present is trochanter.33}FW veins: 0 = IMP connected to MP_1_ (e.g., Fig. 16E, G); 1 = free (e.g., Figs 8D–E, 16A, C).34}FW veins: 0 = MP_2_ much shorter than IMP; 1 = subequal to slightly shorter.35}FW Cu sector, ICus join hind margin on: 0 = both near or anteriad to tornus; 1 = ICu1 close to tornus, ICu2 on basitornal margin.36}FW, max number of cells closed by imv (intercalaries marginal veins): 0 = 3 or more cells; 1 = one or two; 2 = none.37}HW, imv: 0 = absent; 1 = 1 to 4 short; 2 = along the entire margin (or almost), long.38}HW, anal area: 0 = with any or few crossveins; 1 = with many crossveins forming a network.39}Short distal segment on forceps: 0 = present; 1 = absent.40}Pedestal, form: 0 = similar width along its length; 1 = much narrower basally.41}Median remnant of styliger plate: 0 = present; 1 = absent.42}Median remnant of styliger plate, shape of hind margin: 0 = straight or convex; 1 = markedly concave (lateral margins projecting posteriorly, Fig. 12A–D).43}Penial arm articulation 0 = tergite IX; 1 = sternite IX and base of pedestal.44}Penes form: 0 = apices diverging or straight, blade-like; 1 = apices converging medially or curved ventrally.45}Penes, apical spine: 0 = absent or slightly marked as a continuation of the penes; 1 = present, distinctly protruding from the penes (Figure 17).46}Additional penis lobe (thumb): 0 = absent; 1 = present.47}Cleft between large and small penile lobes: 0 = absent; 1 = short, closed (Figure 17D); 2 = long, opened (Fig. 17B, C, F).48}Female sternum VIII, anteromedian keel: 0 = absent; 1 = present.49}Female sternum VIII, anteromedian keel: 0 = short and blunt (Figure 13D); 1 = long and slender (Figs 18F–H, 19E–F).50}Female sternum VIII, sockets: 0 = absent; 1 = present.51}Female sternum VIII, position of sockets: 0 = anterior; 1 = submedian.52}Female sternum VIII, position of sockets: 0 = contiguous; 1 = separated.53}Female sternum VIII, anteromedian sockets: 0 = fused; 1 = small almost indistinct at each side of the keel; 2 = larger extending somewhat posteriorly from keel’s base.54}Eggs, chorionic plates (large disk-like structures, LD in Figure 7E): 0 = absent; 1 = present.55}Eggs, chorionic plates (if present): 0 = seems aggregated small disks (Figure 13A); 1 = entire (e.g., Figure 7E).56}Eggs, chorionic plates (if present and entire): 0 = one piece (Figs 13B–C, 18C); 1 = 2 or more pieces (Fig. 18A–B, D–E).57}Eggs, chorionic plates (small disk-like structures, SD in Figure 7E): 0 = absent; 1 = present.58}Eggs, chorionic plates, location of small disks: 0 = among large disks (e.g., Figure 7E); 1 = below large disks (Figure 18C).59}Eggs, polar caps: 0 = none; 1 = one; 2 = two.60}Clypeus, median proyection: 0 = absent; 1 = present.61}Nymphal left mandibular tusk, number of apical denticles: 0 = four; 1 = three; 2 = one.62}Nymphal right mandibular tusk, number of apical denticles: 0 = two; 1 = one.63}Nymphal mandibular tusks, large basal tubercle on inner margin: 0 = absent; 1 = present. Double-pointed in *Povilla* and *Asthenopus*, but less marked in the last genus (pair of black arrows in Fig. 14H, G, respectively).64}Nymphal mandibular tusks, smaller submedian tubercle on inner margin (e.g., white arrow in Figure 14H): 0 = absent; 1 = present.65}Nymphal mandibular tusks, inner margin distally to smaller submedian tubercle (mentioned above): 0 = smooth; 1 = with many (>5) small tubercles: 2 = with 1 pointed tubercle.66}Nymphal mandibular tusks, small basal tubercle on outer dorsal surface: 0 = absent; 1 = present (Figure 14H).67}Gill on abdominal segment I: 0 = bilamellate 1 = single.68}Nymph, dorsum of head with pilose area on frons: 0 = absent; 1 = present.69}Nymph, occipital region: 0 = strongly expanded, convex; 1 = not strongly developed, flat.70}Nymphs, denticles on foretarsal claws: 0 = absent; 1 = present. When present generally ther is a single row of many marginal denticles, except in *Asthenopodes* where a double row is present.71}Posterolateral spine on tergum X: 0 = absent (Figure 10E); 1 = present (Figure 15E). A small spine may be present, sometimes seeming to arise from paraproct but it actually belongs to the tergum. In *Hubbardipes crenulatus* is very short and blunt. In *Povilla* and *Asthenopus* it is acute, strong and visible dorsally. In Campsurinae it is shorter, blunt and only distinguishable when seen from below. Differing from these groups, in the nymphs of *Asthenopodes* and *Ephoron* it is not expressed.

## Appendix 2

List of apomorphies from terminals and nodes. Arrows separate plesiomorphic from apomorphic states.

*Ephoron*:

No autapomorphies

*Campsurus violaceus*:

Char. 1: 0.395 → 0.357–0.360

Char. 13: 15.000–16.667 → 19.000–23.000

Char. 15: 5.417–5.769 → 2.727–2.846

Char. 17: 1.077–1.083 → 2.600

Char. 21: 1.833 → 2.625–3.333

Cleft between large and small penile lobes (47): absent → long, opened

Female sternum VIII, anteromedian sockets (53): larger extending somewhat posteriorly from keel`s base → fused

*Povilla*:

Char. 3: 0.339–0.347 → 0.316

FW vein IMP (33): free → IMP connected to MP1

*Languidipes*:

Char. 2: 1.661–1.736 → 3.174

Char. 3: 0.339–0.347 → 0.375–0.464

Char. 4: 2.000–2.214 → 2.447

Char. 5: 2.302–2.447 → 3.382

Char. 7: 0.700–1.167 → 1.667

Char. 15: 5.417–6.800 → 9.333

FW Cu sector, ICus join hind margin on (35): both near or anteriad to tornus → ICu1 close to tornus, ICu2 on basitornal margin

Styliger plate, median plate (41): present → absent

Gill on abdominal segment I (67): bilamellate → single

*picteti*:

Char. 0: 1.133–1.188 → 0.838–0.840

Char. 7: 1.400–1.950 → 2.000–3.353

Char. 14: 2.600–2.667 → 2.737–2.895

Char. 15: 5.818–5.909 → 8.000–8.696

Char. 16: 2.065–2.321 → 3.056–3.059

Char. 21: 1.625–2.364 → 1.222–1.516

Eggs, chorionic plates(large disk-like present) (55): entire → seems aggregated small disks

*traverae*:

Char. 12: 8.100 → 11.800

Char. 13: 6.714–7.400 → 4.667–4.900

Char. 17: 1.077–1.083 → 1.000

Char. 22: 3.750–3.778 → 2.143–3.667

*chumuco*:

Char. 9: 0.733–0.838 → 0.560–0.710

Char. 10: 0.337–0.366 → 0.389

Char. 15: 5.417–5.769 → 2.625–3.143

Char. 17: 1.077–1.083 → 2.333–2.667

Char. 19: 0.169–0.184 → 0.089–0.093

Char. 21: 1.625–2.364 → 2.688–3.067

Char. 23: 7.000–13.000 → 14.000–16.000

*crenulatus*:

Char. 0: 1.909–1.923 → 3.250–4.000

Char. 1: 0.645–0.667 → 0.741–0.825

Char. 5: 2.302–2.308 → 2.419–2.654

Char. 12: 4.500–5.200 → 4.286

Char. 14: 2.308–2.500 → 5.909–6.500

Char. 16: 2.000–2.174 → 3.250

Char. 17: 1.077–1.083 → 0.600–0.684

FW vein IMP (33): free → IMP connected to MP1

Nymphal mandibular tusks, smaller submedian tubercle (64): present → absent

*gilliesi*:

Char. 14: 2.077–2.500 → 1.905–2.067

Char. 15: 5.417–5.769 → 3.647–4.706

Char. 16: 2.000–2.174 → 1.177–1.905

Char. 21: 1.231 → 0.892–1.071

Char. 23: 7.000–13.000 → 14.000–16.000

Cleft between large and small penile lobes (47): absent → short, closed

*magnus*:

Char. 0: 1.833–1.923 → 1.968–2.341

Char. 3: 0.286–0.298 → 0.248–0.253

Char. 15: 3.846–4.000 → 2.857–2.933

Char. 16: 2.118–2.174 → 1.905–2.000

Char. 18: 2.222–2.714 → 1.964–2.200

Char. 24: 1.511–1.555 → 1.429–1.489

*angelae*:

Char. 13: 6.154–6.231 → 6.500–7.100

*hubbardi*:

Char. 10: 0.235–0.241 → 0.262–0.283

*guarani*:

Char. 0: 1.909–1.923 → 1.926–1.974

Char. 1: 0.584–0.587 → 0.429–0.579

Char. 7: 0.222–0.300 → 0.000

Char. 18: 2.714–2.913 → 3.125

Char. 24: 1.511–1.555 → 1.408

Char. 25: 1.787–1.811 → 1.818

Char. 27: 2.286–2.303 → 2.630

*curtus*:

Char. 0: 1.833–1.923 → 1.550–1.751

Char. 6: 10.000–11.000 → 18.000–25.000

Char. 10: 0.218–0.241 → 0.171

Char. 12: 3.545–4.400 → 3.222

Char. 14: 1.955–2.000 → 1.786

Char. 16: 2.118–2.174 → 2.273

Char. 17: 1.600–1.667 → 2.167

Char. 19: 0.323–0.353 → 0.378–0.500

Char. 24: 1.511–1.555 → 1.573–1.729

*Campsurus vulturorum*:

Char. 0: 1.765 → 1.864

Char. 10: 0.205–0.222 → 0.168

Char. 11: 1.273–1.333 → 1.000

Char. 14: 2.308–2.500 → 1.415

Char. 17: 1.077–1.083 → 1.071

Char. 24: 1.712 → 1.825

Char. 25: 1.494–1.542 → 1.369

Char. 27: 3.688 → 3.214

*Tortopus*:

Char. 0: 1.300–1.765 → 1.786

Char. 1: 0.533 → 0.536–0.600

Char. 3: 0.370–0.371 → 0.352

Char. 10: 0.205–0.222 → 0.194

Char. 17: 1.077 → 1.000

Char. 19: 0.287–0.319 → 0.277

Char. 26: 1.239–1.258 → 1.283

*Tortopsis*:

Char. 2: 2.044–2.268 → 2.714

Char. 3: 0.370–0.371 → 0.375–0.417

Char. 5: 2.271–2.514 → 2.568

Char. 13: 16.667 → 18.700

Char. 15: 6.364 → 11.923

Char. 19: 0.287–0.319 → 0.321–0.350

Char. 20: 1.226–1.294 → 1.147–1.171

Char. 24: 1.625–1.639 → 1.562

Char. 27: 4.000 → 4.771

Penes, apical spine (45): absent or slightly marked as a continuation of the penes → present, distinctly protruding from the penes

Female sternum VIII, sockets position (52): contiguous → separated

Nymphal mandibular tusks, smaller submedian tubercle (64): present → absent

Node 18 (*Campsurus*):

Char. 1: 0.400–0.533 → 0.395

Char. 9: 0.926–1.500 → 1.580–1.825

Char. 24: 1.625–1.639 → 1.712

Additional penis lobe (46): absent → present

Clypeus, median proyection (60): present → absent

Gill on abdominal segment I (67): single → bilamellate

Node 19 (Campsurinae):

Char. 2: 1.150–1.393 → 1.987

Char. 6: 10.000–12.000 → 6.000–9.000

Char. 7: 1.400–1.950 → 1.000

Char. 10: 0.241–0.250 → 0.205–0.222

Char. 13: 11.667 → 15.000–16.667

Char. 20: 1.322–1.476 → 1.226–1.294

Male foretarsite 1(form) (29): subquadrate → subovate

FW vein IMP (33): free → IMP connected to MP1

FW Cu sector, ICus join hind margin on (35): both near or anteriad to tornus → ICu1 close to tornus, ICu2 on basitornal margin

FW, max number of cells closed by imv (36): >3 → 0

Styliger plate, median plate (41): present → absent

Penial arm articulation (43): tergite IX → S IX and base of pedestal

Nymph, dorsum of head with velvet zone (68): absent → present

Node 20 (Campsurinae + Asthenopodinae):

No synapomorphies in the present analysis but if the root is duplicated, then the following changes define this grouping:

Char. 2: 1.081 → 1.150–1.393

Char. 3: 1.008 → 0.370–0.371

Char. 4: 1.912 → 1.983–2.027

Char. 6: 16.000 → 10.000–12.000

Char. 7: 4.188 → 1.400–1.950

Char. 9: 0.650 → 0.733–1.164

Char. 19: 0.778 → 0.287–0.319

Char. 27: 4.145 → 3.688–4.000

Short distal segments on forceps (39): present → absent

Penes form (44): fused basal 2/3 diverging → apices converging medially or curved ventrally

Female sternum VIII, sockets (50): absent → present

Nymphal mandibular tusks, smaller submedian tubercle (64): absent → present

Node 21 (*Povilla* + *Languidipes*):

Char. 2: 1.529–1.556 → 1.661–1.736

Char. 16: 2.118–2.174 → 4.300–7.000

FW veins MP2 and IMP (34): subequal → MP2 shorter than IMP

Pedestal, form (40): similar width along its length → much narrower basally

Penes form (44): apices converging medially or curved ventrally → apices diverging or straight, blade-like

Nymphal left mandibular tusk, apical denticles (61): 3 → 4

Node 22 (*Asthenopus* + *Povilla* + *Lanquidipes*):

Char. 9: 1.098–1.164 → 1.369–1.653

Char. 13: 6.333–7.692 → 5.385–5.789

Char. 18: 2.647 → 2.714–2.913

Female sternum VIII, anteromedian keel (49): short and blunt → long and slender

Female sternum VIII, anteromedian sockets (53): larger extending somewhat posteriorly from keel’s base → small almost indistinct at each side of the keel

Node 23 (gilliesi + node 22)

Char. 2: 1.150–1.393 → 1.529–1.556

Char. 7: 1.400–1.429 → 0.700–1.167

Char. 13: 7.833–8.889 → 6.333–7.692

Char. 18: 2.188 → 2.647

FW, max number of cells closed by imv (36): >3 → 1–2

Node 24 (Asthenopodinae except *Asthenopodes*):

Char. 0: 1.211–1.765 → 1.909–1.923

Char. 3: 0.364–0.371 → 0.339–0.347

Char. 5: 2.167–2.271 → 2.302–2.308

Char. 12: 5.455 → 4.500–5.200

Char. 18: 2.000–2.071 → 2.188

Char. 21: 1.625–1.833 → 1.231–1.375

Char. 22: 3.750–4.000 → 4.083–4.333

Char. 24: 1.625–1.639 → 1.577

Gill on abdominal segment I (67): single → bilamellate

Node 25 (Asthenopodinae):

Char. 1: 0.400–0.533 → 0.645–0.667

Char. 13: 11.667 → 7.833–8.889

Char. 19: 0.287–0.319 → 0.169–0.184

Char. 27: 3.688–4.000 → 2.196–2.412

Female sternum VIII, anteromedian keel (48): absent → present

Eggs, chorionic plates(large disk-like) (54): absent → present

Eggs, chorionic plates(small disk-like) (57): absent → present

Nymphal left mandibular tusk, apical denticles (61): 1 → 3

Nymphal right mandibular tusk, apical denticles (62): 1 → 2

Nymphal mandibular tusks, small basal tubercle on outer dorsal surface (66): absent → present

Nymph, occipital region (69): not strongly developed, flat → strongly expanded, convex

Nymphs, denticles on fore tarsal claws (70): absent → present

Node 26 (*Asthenopodes traverae* + *Asthenopus picteti*):

Char. 1: 0.645–0.667 → 1.205–1.320

Char. 6: 10.000–12.000 → 14.000–16.000

Char. 12: 6.333–6.667 → 8.100

Char. 13: 7.833–8.889 → 6.714–7.400

Char. 14: 2.380–2.500 → 2.600–2.667

Char. 15: 5.417–5.769 → 5.818–5.909

Char. 23: 7.000–13.000 → 6.000

Styliger plate, median plate, shape of hind margin (42): straight or convex → markedly concave (lateral margins projected posteriorly)

Node 27 (*Asthenopodes*):

Char. 0: 1.211–1.765 → 1.133–1.188

Char. 5: 2.167–2.271 → 1.983–2.047

Char. 10: 0.241–0.250 → 0.337–0.366

Char. 12: 5.455 → 6.333–6.667

Apex of male foretarsal claw (31): not or slightly expanded → strongly expanded (apex 3 times wider than stalk)

Pedestal, form (40): similar width along its length → much narrower basally

Node 28 (*Asthenopus angelae* + *Asthenopus magnus*):

Char. 13: 5.385–5.789 → 6.154–6.231

Cleft between large and small penile lobes (47): long, opened → short, closed

Node 29 (*Asthenopus curtus* + *Asthenopus angelae* + *Asthenopus magnus*):

Char. 2: 1.529–1.556 → 1.456–1.457

Char. 3: 0.339–0.347 → 0.286–0.298

Char. 19: 0.292 → 0.323–0.353

Node 30 (*Asthenopus hubbardi* + *Asthenopus curtus* + *Asthenopus angelae* + *Asthenopus magnus*):

Char. 9: 1.369–1.653 → 1.730–1.850

Char. 14: 2.077–2.476 → 1.955–2.000

Char. 15: 5.417–5.778 → 3.846–4.500

Char. 17: 1.125–1.300 → 1.600–1.667

Node 31 (*Asthenopus* s.s.):

Char. 1: 0.645–0.652 → 0.584–0.587

Char. 7: 0.700–1.167 → 0.222–0.300

Male foretarsite 1(form) (29): subquadrate → subrectangular

Penes, apical spine (45): absent or slightly marked as a continuation of the penes → present, distinctly protruding from the penes

Cleft between large and small penile lobes (47): absent → long, opened

Clypeus, median proyection (60): present → absent

Node 32 (*Tortopsis* + *Tortopus*):

Char. 2: 1.987 → 2.044–2.268

Char. 6: 6.000–9.000 → 1.000

Char. 7: 1.000 → 0.000

Char. 15: 5.417–5.769 → 6.364

Fore tarsite 5 apex (30): blunt or transv ridge → trilobed

HW, anal area (38): with any or few crossveins → with many crossveins forming a network

Female sternum VIII, sockets position (51): anterior → submedian

## Appendix 3

Matrix of characters and states. Ready to use in TNT.

nstates cont;

xread

‘Asthenopodinae’

72 17

&[continuous]

Ephoron 1.211 0.400 0.932-1.081 1.008-1.123 1.857-1.912 1.846-2.167 16.000 4.188 3.000 0.650 0.250 1.273 5.455 11.667 ? ? ? ? ? 0.778-0.992 1.322-1.551 0.727-1.625 2.000-5.000 ? 1.625 ? ? 4.145

Camp_violaceus 1.765 0.357-0.360 1.758-2.179 0.371-0.436 2.082-2.273 2.236-2.346 2.000-6.000 1.000-1.667 1.000-3.000 1.580-1.830 0.205-0.261 1.333-1.440 ? 19.000-23.000 2.308-2.552 2.727-2.846 1.304-1.500 2.600 1.118-1.410 0.287-0.375 1.091-1.300 2.625-3.333 3.000-4.000 ? 1.712 1.542 1.148-1.443 3.688

Povilla 1.571-2.000 0.645-0.755 1.661-1.736 0.316 1.831-2.214 2.095-2.447 1.000-15.000 0.467-0.700 1.000-2.000 1.324-1.801 0.100-0.235 3.333-3.928 3.375-5.429 4.333-7.428 4.400-6.800 4.667-6.800 4.300-7.000 1.300 2.857-4.167 0.068-0.260 1.457-1.750 ? ? 13.000 1.555 1.787 1.439 2.015

Languidipes ? ? 3.174 0.375-0.464 2.447 3.382 5.000 1.667 1.000 1.653 ? ? ? 4.545 ? 9.333 7.000 ? 2.913 0.100 ? ? ? ? ? ? ? ?

picteti 0.838-0.840 1.205-1.350 1.118-1.150 0.355-0.382 1.828-1.983 1.828-1.983 14.000-17.000 2.000-3.353 4.000-6.000 0.595-0.733 0.337-0.373 1.550-1.875 8.100 6.714-7.400 2.737-2.895 8.000-8.696 3.056-3.059 1.083-1.182 1.500-2.250 0.184-0.206 1.383-1.591 1.222-1.516 3.778-5.167 6.000 ? ? ? ?

traverae 1.133-1.192 1.320-1.478 1.022-1.183 0.364-0.406 1.895-1.986 1.957-2.059 16.000-22.000 0.750-1.950 4.000-5.000 0.838-0.890 0.223-0.366 1.310-1.880 11.800 4.667-4.900 2.600-2.667 5.818-5.909 2.065-2.321 1.000 2.000-2.071 0.180-0.203 1.476-1.605 2.364-3.000 2.143-3.667 5.000-6.000 ? ? ? ?

chumuco 1.097-1.188 0.619-0.667 1.393-1.600 0.318-0.440 1.870-2.000 1.892-2.047 9.000-12.000 1.111-2.833 3.000-4.000 0.560-0.710 0.389 1.243-1.286 6.333-6.667 7.833-9.500 2.380-2.500 2.625-3.143 1.833-2.000 2.333-2.667 2.000-2.133 0.089-0.093 1.313-1.413 2.688-3.067 2.500-3.750 14.000-16.000 1.829-2.055 ? 1.258-1.226 1.978-2.412

crenulatus 3.250-4.000 0.741-0.825 1.087-1.150 0.329-0.347 2.027-2.091 2.419-2.654 10.000-14.000 1.400-1.429 2.000-4.000 1.164-1.795 0.241 1.550-2.462 4.286 8.889-9.000 5.909-6.500 5.000-5.417 3.250 0.600-0.684 2.188 ? 1.462-1.677 1.192-1.375 4.083-6.750 4.000-7.000 1.577 ? ? 2.196

gilliesi 1.411-1.909 0.523-0.652 1.556-1.975 0.300-0.350 1.781-2.079 2.054-2.308 4.000-10.000 0.250-1.167 1.000-2.000 0.780-1.098 0.191-0.308 2.143-2.500 4.500-6.143 6.333-7.692 1.905-2.067 3.647-4.706 1.177-1.905 0.944-1.5 2.647 0.169 1.455-1.833 0.892-1.071 2.800-6.167 14.000-16.000 ? ? ? ?

magnus 1.968-2.341 0.554-0.655 1.365-1.456 0.248-0.253 1.870-2.022 2.104-2.302 10.000-22.000 0.091-0.318 0.000-2.000 1.690-3.370 0.207-0.257 1.719-2.400 3.545-6.167 6.154-6.231 2.000-2.222 2.857-2.933 1.905-2.000 1.556-1.667 1.964-2.200 0.323-0.389 1.594-1.781 1.176-1.261 3.286-4.333 4.000-5.000 1.429-1.489 1.714-1.811 1.319-1.396 2.286-2.500

angelae 1.491-1.833 0.592-0.596 1.359-1.635 0.298-0.309 1.889-2.201 2.122-2.560 5.000-11.000 0.000-0.300 1.000-4.000 1.375-2.890 0.218-0.275 2.083-2.190 3.000-4.400 6.500-7.100 1.958-2.282 4.000-4.909 2.174-3.067 1.429-2.000 2.222-2.889 0.250-0.353 1.533-1.667 1.111-1.320 3.125-4.500 3.000-5.000 1.511-1.624 1.741-1.950 1.389-1.427 2.171-2.303

hubbardi 1.923-2.133 0.530-0.587 1.529-1.769 0.339-0.355 2.000-2.091 2.300-2.600 4.000-14.000 0.143-0.500 2.000-3.000 1.730-1.850 0.262-0.283 2.100 3.571-5.125 4.692-6.000 1.714-1.955 3.583-4.500 1.870-2.118 1.600-1.800 2.714 0.292 1.448-1.741 1.111-1.318 3.143-4.500 3.000-8.000 ? ? ? ?

guarani 1.926-1.974 0.429-0.579 1.381-1.765 0.268-0.354 1.744-2.000 1.829-2.500 4.000-6.000 0.000 1.000-2.000 1.170-1.369 ? 2.150 5.200 4.833-5.789 2.077-2.476 5.778-6.000 2.080-2.600 1.125 3.125 ? 1.250-1.600 1.231 4.333 3.000-8.000 1.408 1.818 1.351 2.630

curtus 1.550-1.751 0.584 1.365-1.457 0.248-0.286 1.870-2.022 2.104-2.341 18.000-25.000 0.222-0.316 0.000-1.000 2.100-2.950 0.171 1.964-2.320 3.222 5.385 1.786 3.846 2.273 2.167 2.706-3.059 0.378-0.500 1.419-1.545 1.128-1.341 4.000-6.286 3.000-5.000 1.573-1.729 1.714-2.371 1.395-1.618 2.182-2.419

Camp_vulturorum 1.864 0.395 1.987 0.370 1.983 2.271 9.000 1.000 1.000 1.825 0.168 1.000 ? 15.000 1.415 5.769 ? 1.071 1.146 0.257-0.319 1.224-1.294 1.833 ? ? 1.825 1.369 1.117-1.237 3.214

Tortopus 1.786 0.536-0.600 2.044-2.268 0.352 ? 2.190-2.514 1.000 0.000 2.000 1.500 0.194 ? ? 16.667 ? 6.364 ? 1.000 ? 0.277 1.226-1.333 ? ? ? 1.639 1.494 1.283 4.000

Tortopsis 1.300 0.533 2.714 0.375-0.417 2.169 2.568 0.000-1.000 0.000 1.000-3.000 0.926 0.222 ? ? 18.700 ? 11.923 ? 1.077 1.407 0.321-0.350 1.147-1.171 ? ? ? 1.562 ? 1.239 4.771

&[numeric]Ephoron100001200200000020000-0---0--0-1121000010100Camp_violaceus02003001200201?110120-10000--0-0021011001101Povilla0000100011011000000011100110-0-0100110100011Languidipes00-01000110111?-0000-----------?10011011001-picteti1-01111002021010100010100210-102------------traverae1-011110020210101000101002110102------------chumuco1-011110020210001000101002110102010010110010crenulatus20001010020200001000101002111102110000100011gilliesi0000111010020000101110100210-102------------magnus010011101102000011111110011110-2010110100011angelae010011101102000011111110011110-2010110100011hubbardi010011101102000011121110011110-2------------guarani01001110110200001112111001110112010110100011curtus010011101102000011121110011110-2010110100011Camp_vulturorum02003001200201-1-0100-10020--0-0021011001101Tortopus02102001211101-110000-11020--0-0121012011101Tortopsis02102001211101-111000-11120--0-0121002011101; cc -.;proc/;
